# Electronic structure studies reveal 4f/5d mixing and its effect on bonding characteristics in Ce-imido and -oxo complexes†

**DOI:** 10.1039/d1sc06623d

**Published:** 2022-01-24

**Authors:** Liane M. Moreau, Ekaterina Lapsheva, Jorge I. Amaro-Estrada, Michael R. Gau, Patrick J. Carroll, Brian C. Manor, Yusen Qiao, Qiaomu Yang, Wayne W. Lukens, Dimosthenis Sokaras, Eric J. Schelter, Laurent Maron, Corwin H. Booth

**Affiliations:** Chemical Sciences Division, Lawrence Berkeley National Laboratory Berkeley CA 94720 USA; Department of Chemistry, University of Pennsylvania Philadelphia PA 19104 USA; LPCNO, UMR 5215, CNRS, INSA, UPS, Université de Toulouse 31000 Toulouse France; Stanford Synchrotron Radiation Lightsource, SLAC National Accelerator Laboratory Menlo Park CA 94025 USA

## Abstract

This study presents the role of 5d orbitals in the bonding, and electronic and magnetic structure of Ce imido and oxo complexes synthesized with a tris(hydroxylaminato) [((2-^*t*^BuNO)C_6_H_4_CH_2_)_3_N]^3−^ (TriNO_*x*_^3−^) ligand framework, including the reported synthesis and characterization of two new alkali metal-capped Ce oxo species. X-ray spectroscopy measurements reveal that the imido and oxo materials exhibit an intermediate valent ground state of the Ce, displaying hallmark features in the Ce L_III_ absorption of partial f-orbital occupancy that are relatively constant for all measured compounds. These spectra feature a double peak consistent with other formal Ce(iv) compounds. Magnetic susceptibility measurements reveal enhanced levels of temperature-independent paramagnetism (TIP). In contrast to systems with direct bonding to an aromatic ligand, no clear correlation between the level of TIP and f-orbital occupancy is observed. CASSCF calculations defy a conventional van Vleck explanation of the TIP, indicating a single-reference ground state with no low-lying triplet excited state, despite accurately predicting the measured values of f-orbital occupancy. The calculations do, however, predict strong 4f/5d hybridization. In fact, within these complexes, despite having similar f-orbital occupancies and therefore levels of 4f/5d hybridization, the d-state distributions vary depending on the bonding motif (Ce=O *vs.* Ce=N) of the complex, and can also be fine-tuned based on varying alkali metal cation capping species. This system therefore provides a platform for understanding the characteristic nature of Ce multiple bonds and potential impact that the associated d-state distribution may have on resulting reactivity.

## Abstract 2

Ce(iv) complexes with multiple bonds display similar f^0^ fractions, but different f/d hybridization, 5d-orbital energies, and TIP levels.

## Graphical abstract

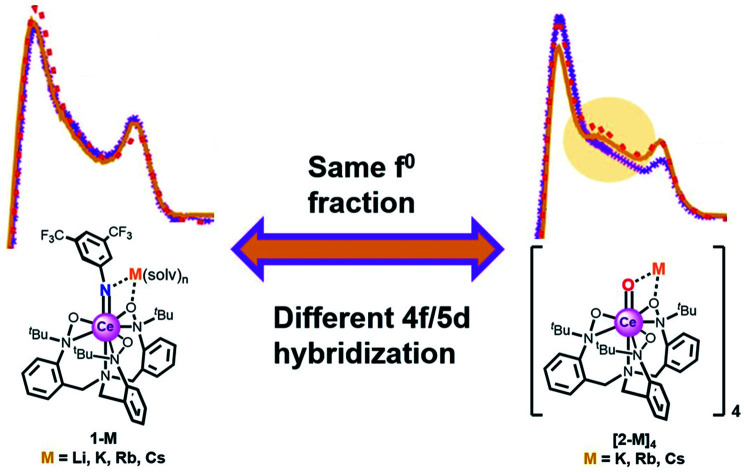

## Introduction

The canonical view of lanthanide 4f-orbitals is that they are localized enough that they don't participate in bonding, or, in metals, in conductivity. In addition, their localization imparts local moment magnetism, whereby the orbital angular momentum is not quenched in contrast to typical transition metal d-orbital systems. This view, however, has been shown to be incorrect in that within certain lanthanide intermetallic materials, mixed valence is implicated in low-temperature temperature-independent paramagnetism (TIP) and in heavy-fermion conductivity.^1^ In such materials, the many–body interactions between the f-orbital and the conduction band (for example, the 5d orbital in elemental Ce metal^2^ or the Cu 3d orbital in YbIn_1−*x*_Ag_*x*_Cu_4_^3^) are an important feature in determining the lanthanide (Ln) 4f orbital occupancy and magnetic properties. More recently, various Ce and Yb organometallic molecules, including Ce(C_8_H_8_)_2_ (cerocene)^4–10^ and Cp*_2_Yb(bipy) (bispentamethylcyclopentadienyl ytterbium bipyridyl),^8,11^ among others,^12–17^ have been shown to exhibit similar f-orbital mixing, not with a fully metallic band, but rather with delocalized orbitals from aromatic rings proximal to the metal center. In these materials, single-electron theories such as DFT typically indicate no mixing of the 4f/ligand orbitals, while theories that account for configuration interactions between various magnetic singlets show a mixed ground state with non-integral occupancy of the 4f orbital, allowing for enhanced TIP behavior from a van Vleck mixing of an excited state triplet. In cerocene,^4–10^ for example, the ground state is primarily a quantum mechanical mixture of f^0^ and f^1^ configurations (closed- and open-shell singlet configurations, respectively), with a fractional f-occupancy of about 0.89 despite its formal oxidation state assignment as Ce(iv) based on a typical (C_8_H_8_)^2−^, and a van Vleck-induced TIP susceptibility of *χ*_0_ = 1.4 × 10^−4^ emu mol^−1^. Other formal Ce(iv) molecules have recently been shown to display similar behavior.^12^ More recent advances by Sergentu *et al.*^18^ have importantly confirmed that the doublet peak observed in Ce(iii)/Ce(iv) systems is due to differences in the core hole interactions between f^1^ and f^0^ configurations. Additionally, they emphasized the f^2^ contribution to what had been assigned as a pure f^1^ spectral peak (although less than 10%), and therefore what has been traditionally referred to as the f^1^ configuration is more accurately described as f^1,2^.

**Scheme 1:**
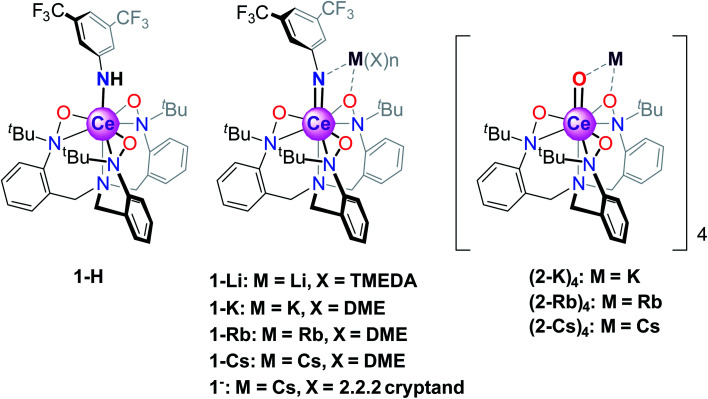
Structures of the Ce(iv) anilide 1-H, imido 1-M, and oxo (2-M)_4_ compounds.

In the aforementioned systems, the Ln 5d orbital does not play a crucial role. This is in contrast to what is believed to be true for inorganic compounds such as CeO_2_ and Ce_2_O_3_, where magnetism has no clearly measurable contribution from the f-orbitals,^12^ likely due to the stronger delocalization of the f-electrons from hybridization with the ligand 2p and metal 5d orbitals. Unfortunately, CAS methods used to elucidate electronic characteristics in organometallic molecules are not currently applicable to extended solids, such as CeO_2_. It would therefore be of interest to consider the behavior of formal Ce(iv) organometallics where Ce forms metal–ligand multiple bonds, which could serve as molecular analogs to CeO_2_. In addition, the notable reactivity patterns observed for transition metal oxo and imido compounds, which stem from the iminyl/oxyl character,^19–33^ prompt the investigation of electronic structures and spectroscopic properties of lanthanide systems with metal–ligand multiple bonds. Towards this end, we consider a class of Ce imido and oxo complexes which are designed to enhance 5d participation while de-emphasizing ligand 2p orbital mixing. Recently, several alkali metal-capped Ce(iv) imido compounds [M(solv)_*n*_][Ce=NAr^F^(TriNO_*x*_)] (solv = DME or TMEDA, M = Li^+^, K^+,^ Rb^+^, Cs^+^, Ar^F^ = 3,5-(CF_3_)_2_–C_6_H_3_, TriNO_*x*_^3−^ = (*t*BuN(O))C_6_H_4_CH_2_}_3_N]^3−^, 1-M) and the uncapped congener [Cs(2.2.2 cryptand)][Ce=NAr^F^(TriNO_*x*_)] (1^−^), as well as the parent anilide compound Ce–NHAr^F^(TriNO_*x*_) (1-H) have been reported (Scheme 1).^34^ These compounds feature the TriNO_*x*_^3−^ ligand, which affords stabilization of the Ce(iv) oxidation state, protecting these compounds against reduction. Following isolation of the Ce(iv)–imidos and the uncapped congener, we isolated the cerium oxo complex Rb_4_ [Ce=O (TriNO_*x*_)]_4_ (2-Rb)_4_ through an aza-Wittig reaction.^34^

With this synthetic foothold established, in the current work, we present a detailed examination of the electronic structures of the cerium(iv)–anilide complex (1-H), select alkali metal-capped cerium imidos, 1-M, complexes and their uncapped congener, 1^−^, as well the alkali metal-capped oxo, (2-K)_4_, M = K, Rb, Cs, compounds outlined in Scheme 1. A chief challenge in the study of the cerium congeners is the participation of both the 4f- and 5d-orbitals in the valence electronic structure, and as will be shown below, considering both sets of orbitals is necessary to begin to understand these systems, and, in turn, to gain insight into Ce–ligand multiple bonding characteristics. Here, the 4f- and 5d-orbitals are probed through a union of synthesis, structural characterization, magnetometry, and Ce L_III_-edge X-ray absorption spectroscopy (XAS) techniques, combined with complete active space self-consistent field (CASSCF) calculations. The results of this combined investigation into electronic structure reveal that fractional f-occupancy is less impacted by structural differences between the imido and oxo constructs than d-state distributions controlled by bonding characteristics and alkali metal capping cation species. In contrast to cerocene and previously studied systems that involve direct bonding to an aromatic ligand, these structures present a single-reference ground state with strong 4f/5d hybridization. Ultimately, the insight gained from this study can be applied towards understanding how bonding characteristics can affect the electronic properties of Ln organometallic complexes.

## Results

### Synthesis and X-ray

**Fig. 1:**
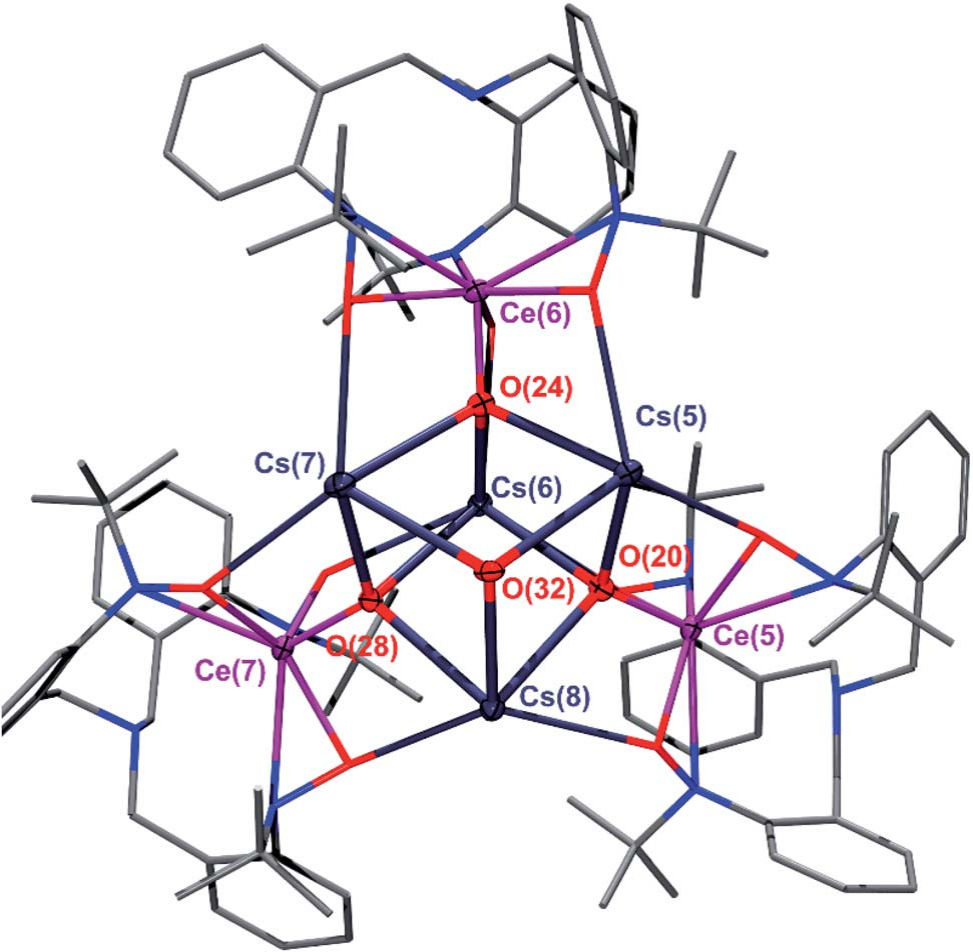
The tetrameric core of the (2-Cs)_4_ cluster. Thermal ellipsoids are shown at 30% probability. Hydrogen atoms have been omitted for clarity. TriNO_*x*_^3−^ ligand framework depicted using a wireframe model.

For our expanded electronic structure studies of Ce(iv)–oxo compounds, we report the synthesis of the tetramer oxo complexes K_4_[Ce=O(TriNO_*x*_)]_4_(2-K)_4_ and Cs_4_[Ce=O(TriNO_*x*_)]_4_(2-Cs)_4_. These compounds were prepared through an aza-Wittig-type reaction by reacting the corresponding imido compounds 1-K and 1-Cs with benzophenone and isolated in 59% and 39% yield, respectively. The relatively low yield of (2-Cs)_4_ was attributed to its increased solubility compared to (2-K)_4_. Compounds (2-K)_4_ and (2-Cs)_4_ were crystallized from hot, concentrated THF solutions set undisturbed at RT. Complexes (2-K)_4_ and (2-Cs)_4_ are poorly soluble in most organic solvents and exhibit limited solubility in THF. In both cases, ^1^H NMR spectroscopy of THF-*d*_8_ solutions revealed the formation of a *C*_3_-symmetric, cerium-containing, product (Fig. S2 and S5†). Complex (2-K)_4_ indicated a solution magnetic susceptibility of *χ*_m_ = 3.3 × 10^−3^ emu mol^−1^ following measurement by Evans method, a value that was largely consistent with the solid state magnetic susceptibility, *vide infra*. Evans method measurement of (2-Cs)_4_ indicated a diamagnetic complex in solution. The characteristic doublets of diastereotopic protons of the CH_2_ groups originating from the TriNO_*x*_-ligand were observed in the ^1^H NMR spectra at 4.17 and 2.26 ppm for (2-Cs)_4_, and 4.16 and 2.25 ppm for (2-K)_4_. An X-ray structural study of (2-K)_4_ and (2-Cs)_4_ revealed the tetrameric structural motif that was essentially identical to the one reported for (2-Rb)_4_.^34^ In all cases, the alkali metal cations aggregated with [(TriNO_*x*_)Ce=O]^−^ units to form the tetramer. The TriNO_*x*_^3−^ ligands provided steric protection for the M_4_Ce_4_O_4_ core, where M = K, Cs, Rb. The structural motif inside the core was a cube-like M_4_O_4_ arrangement, where each oxygen atom was located at four vertices of the cube, with three adjacent vertices occupied by alkali metal cations, resulting in the Ce=O moiety being capped by three alkali metal cations (Fig. 1 and S1†). The distances between the alkali metal cation and the oxygen atom of the Ce=O moieties ranged from 2.638–2.671 Å for (2-K)_4_ and from 2.893–2.966 Å for (2-Cs)_4_. As with (2-Rb)_4_, the structural arrangement of (2-K)_4_ and (2-Cs)_4_ evidently stabilizes the Ce=O moiety.^34^ The Ce=O bond lengths ranged from 1.878(4)–1.885(4) Å for (2-K)_4_ and from 1.876(4)–1.887(4) Å for (2-Cs)_4_, similarly to the Rb congener.^34^ This range of bond distances is somewhat longer than the 1.857(3) Å Ce=O distance in acetamide-capped terminal oxo compound reported by the Leung group.^35^ However, the Ce=O bond distances in (2-K)_4_ and (2-Cs)_4_ were shorter than in the monomeric, previously reported cerium oxo complex capped with a [Li-12-crown-4]^+^ cation reported by Hayton, where this distance was 1.902(2) Å.^36^

#### X-ray absorption near-edge structure (XANES)

Before continuing to present the results of this study, we review the salient points of recent results concerning both single *versus* multiconfiguration terminology and using Ln L_3_-edge XANES in the context of determining f-orbital occupancy in hybridized systems, including those governed by conventional covalency and multiconfigurational systems. It is important to recognize that a system that is describable with a single configuration that is strongly hybridized, *i.e.* covalently bonded to the lanthanide center, can also be described as multiconfigurational through a rotation of the active space, although the reverse is not always true (*e.g.* cerocene).^37^ With this fact in mind, although the Ln L_3_-edge XANES technique has been shown to be accurate in a variety of solid-state contexts (as mentioned above and many others), theoretical determinations have necessarily included assumptions to make these large-system calculations.^38,39^ Consequently, concerns about the role of the 2p_3/2_ core hole in the final (excited) state of the XANES measurement have persisted. Recently, Sergentu *et al.*^18^ have made detailed CAS calculations both of the ground state and of the excited state including the 2p_3/2_ core hole on both cerocene and CeO_2_. Instructively, they have shown that, as expected, the two-peak feature observed in mixed Ce(iii)/Ce(iv) systems (*e.g.* CeO_2_ has an f-occupancy near ½ due to covalent mixing of the otherwise-empty 4f orbital and the oxygen 2p orbital) is indeed due to the difference in the core hole excited-state interaction between the f^0^ and the f^1^ configurations. Both the presence and the relative weight of the spectroscopic features are reflective of the ground state properties and the XANES technique is, therefore, a quantitative way of extracting relative orbital occupancies of the ground state. However, while already understood, the work emphasized that such multiple-peak structure can exist either due to conventional covalent (single configurational) mixing or by multiconfigurational mixing, and is therefore not, by itself, an indication of multiconfigurational character. Another important point is that any f^2^-orbital contribution (which must be present in mixed valent Ce systems as a consequence of the Brillouin theorem), while typically <10%, is explicitly shown by Sergentu *et al.* to contribute to the weight of what has typically been considered as “the f^1^ peak” and has likely contributed to small errors in f-orbital occupancy values reported in the literature.^18^ We therefore have instead chosen to report the f^0^-orbital fraction, *n*(f^0^), here to avoid this potential systematic error. These points are important to the presentation of the results, and so will be repeated and expanded below.

**Fig. 2:**
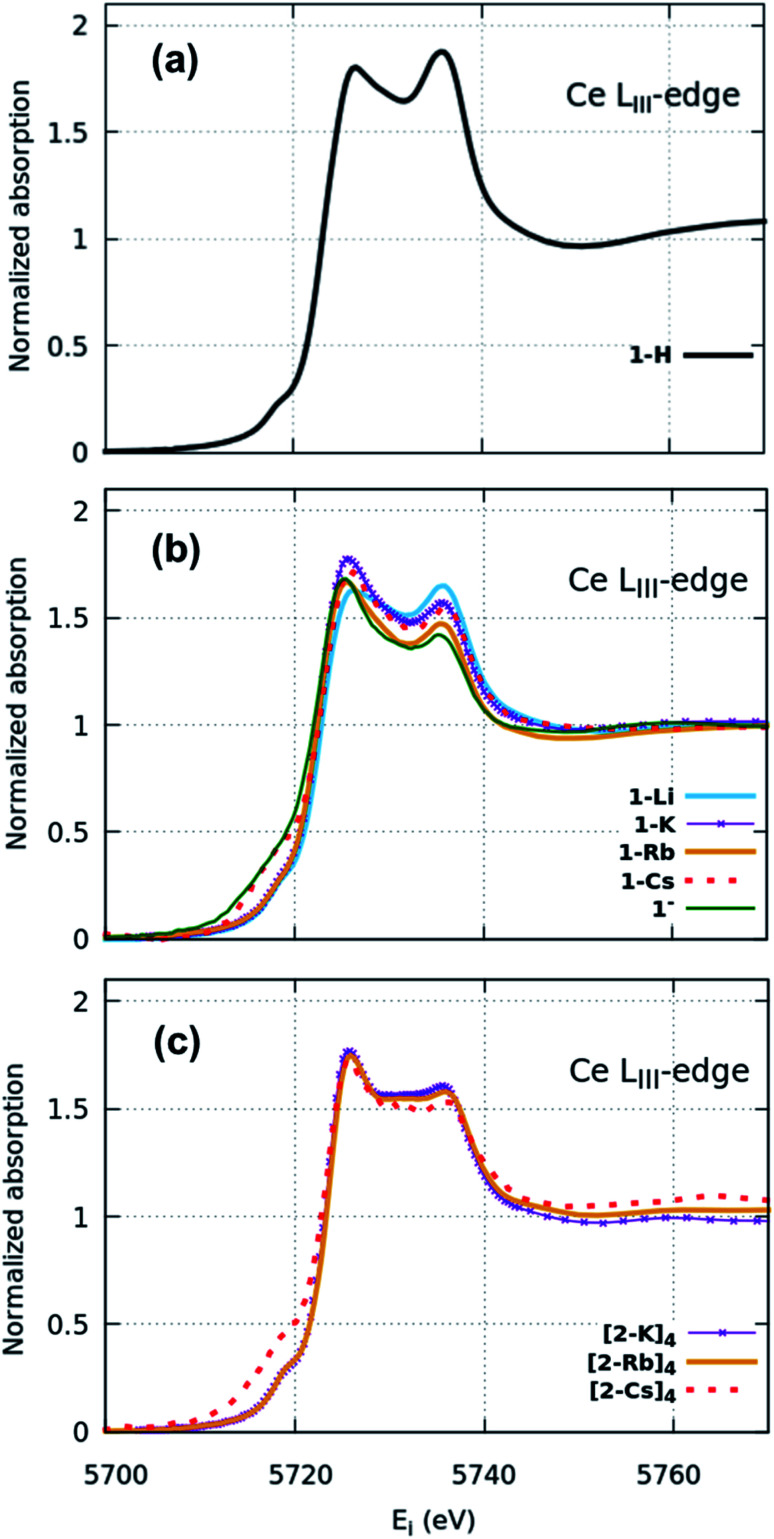
Ce L_III_ edge XANES spectra of the (a) anilide 1-H, (b) imido 1-M and (c) oxo (2-M)_4_ samples. Each sample features the doublet peak characteristic to formal Ce(iv) but with differences in line shape. Increased amplitude below 5720 eV in the 1-Cs, 1^−^, and [2-Cs]_4_ data is due to the Cs L_I_ edge.

XANES spectra (Fig. 2) indicate that both the 1-H starting material as well as 1-M, 1^−^ and (2-M)_4_ samples can all be considered as formal Ce(iv). Similar spectra have been widely used to determine the f-orbital occupancy, although the relationship between the ground state and the actual spectra, which represent a final state with a 2p_3/2_ core hole, has, until recently, not been well understood.^18,40^ In all measured samples here, the double peak characteristic to formal Ce(iv) is observed and confirmed by comparisons to a Ce(iii) and a Ce(iv) standard (Fig. S12†). All data were collected at multiple temperatures (50 K and 300 K) and exhibit no temperature-dependent changes in the spectra (Fig. S13†). Note that in Ce(iv) XANES, the leading-edge peak near 5726 eV indicates primarily the degree of f^1^ character due to covalence with the ligand,^18,40,41^ since this fraction will screen the 2p_3/2_ core hole and shift the 2p_3/2_ → 5d_5/2_ transition to lower energy compared to the peak near 5736 eV that corresponds to the f^0^ part of the wavefunction. Since the lower energy peak should also include a small contribution from f^2^ character,^11,18^ hereafter, we refer to these peaks as the “f^1,2^” and “f^0^” peaks, respectively, recognizing that the predominant contribution to the f^1,2^ peak is due to the f^1^ configuration. We do, however, emphasize that these peaks are not due to transitions into f states, but rather are due primarily to dipole transitions into empty 5d_5/2_ states and this notation refers to energy shifts of these states depending on the f-orbital configuration. There is also a small peak at ∼5718 eV which has been attributed to arising either from a 2p–4f quadrupole excitation^42^ or from mixed d- and f-states.^43^ This peak is notably larger in the 1-Cs, 1^−^ and [2-Cs]_4_ due to the additional contribution from the Cs L_I_ absorption edge at 5714 eV in these samples, but otherwise appears similar between the other samples. The 1-H data appear to have the greatest ratio of peak intensities between the f^0^ peak at higher energy and the f^1,2^ peak at lower energy compared with the 1-M, 1^−^ and (2-M)_4_ complexes. The imido spectra also have well-defined f^1,2^ and f^0^ peaks, but with visually higher peak intensities for the f^1,2^ feature compared with the anilide. The oxo spectra, in contrast, while featuring the double-peak characteristic to Ce(iv), also have a flat region in the middle of the two characteristic peaks indicative of other contributions. The origin of this feature was investigated using HERFD-XAS and is described below.

Surprisingly, no significant differences in the f^1,2^ and f^0^ peaks were observed in the imido or oxo spectra as a function of alkali metal capping species. The only exception to this trend is 1-Li, which appears to have a slightly lower f^1,2^ peak than the other imido counterparts. These trends were further explored through fitting the areas under the f^1,2^ and f^0^ peaks (see Methods) in order to extract the f^0^-configuration fraction, *n*(f^0^), within each complex (Table 1, Fig. S11 and Table S1†). Reporting *n*(f^0^) rather than the traditional use of the f-orbital occupation, *n*_f_, is necessary because we currently have no way of knowing the significance of any f^2^ configuration contribution to the f^1,2^ peak, which has been calculated^18^ to be separated by only about 5 eV and is therefore convolved with any 5d ligand field splitting. Previous estimates of n_f_ took the fraction of the f^1,2^-peak area compared to the total area of the f^0^ and f^1,2^ peaks with effectively *n*_f_ = 1 − *n*(f^0^), and are therefore in error insofar as the f^2^ configuration fraction exceeded ∼3% (the best estimate of the systematic error using this technique). We therefore follow ref. 18 and report the fraction of the well-resolved f^0^ peak to the total area of the f^0^ and f^1,2^ peaks, which corresponds to the f^0^ configuration fraction, *n*(f^0^).

**Table 1:** Summary of electronic and structural parameters

Sample	*n*(f^0^)^a^	*χ*_0_ (emu mol^−1^)	Calculated ground to excited state s–t gap (Δ eV)
1-H	0.45(3)	1.10(6) × 10^−4^	N/A
1-Li	0.42(3)	2.3(2) × 10^−4^	N/A
1-K	0.40(3)	2.2(2) × 10^−4^	1.51
1-Rb	0.37(3)	2.5(4) × 10^−4^	0.84
1-Cs	0.41(3)	3.1(2) × 10^−4^	0.51
1^−^	0.37(3)	3(1) × 10^−4^	N/A
(2-K)_4_	0.41 (3)	4.2(5) × 10^−4^	2.60
(2-Rb)_4_	0.39(3)	1.4(4) × 10^−4^	2.22
(2-Cs)_4_	0.41(3)	3.9(3) × 10^−4^	N/A

a Error bars on *n*(f^0^) are estimated to be ±0.03 electrons based on variability in samples across multiple data sets. Values reported represent an average *n*(f^0^) where multiple data sets were measured.

Fitting results from a two-peak model suggest a narrow range for all samples. The anilide has a slightly higher *n*(f^0^) value (0.45) than the imidos and oxos, which matches the observable difference in relative peak intensity. All imido and oxo samples have an *n*(f^0^) value of ∼0.4, without any perceivable trend based on alkali metal capping species or imido *versus* oxo bonding character. This lack of a trend is particularly interesting given the clear differences observed in the spectral features between the imido and oxo samples.

#### High energy-resolution fluorescence detection (HERFD)

**Fig. 3:**
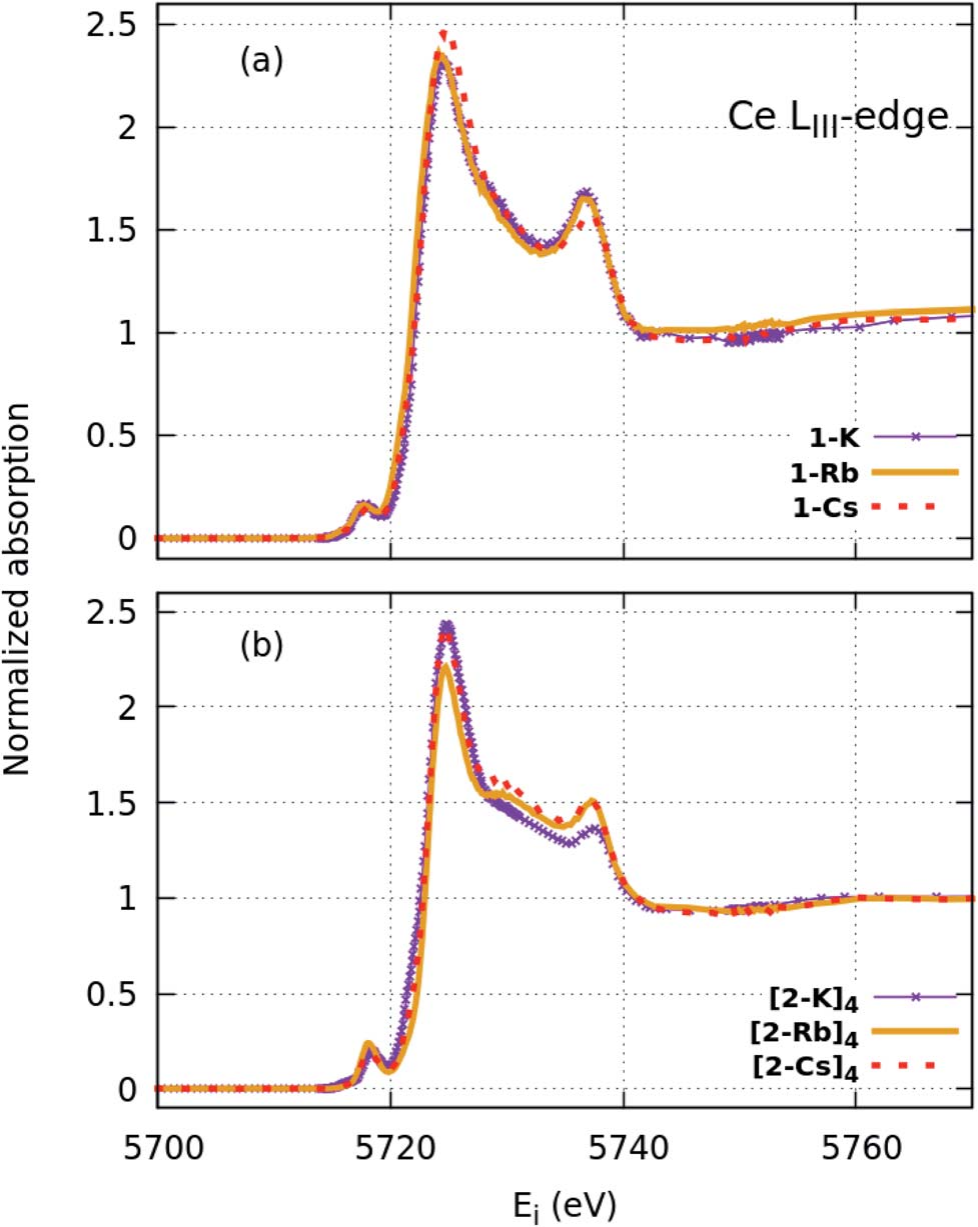
HERFD-XAS spectra collected at the Ce L_III_ edge for the (a) imido and (b) oxo complexes. Greater spectral detail can be resolved compared with XANES (Fig. 2).

HERFD spectroscopy was used for the purpose of highlighting features from Ce L_III_-edge XANES in higher spectral resolution, given that the resolution in this case is dominated by the 3d_5/2_ core-hole lifetime broadening (0.87 eV) rather than by the 2p_3/2_ core–hole lifetime broadening (3.2 eV)^44^ that dominates conventional L_III_-edge XANES. This enables observation of features that are not resolvable from XANES and provides additional insight into complex electronic structure. In all HERFD-XAS spectra (Fig. 3, S17 and S19†), three main peaks are observed. These include the f^1,2^ and f^0^ peaks observed in conventional XANES as well as the more-resolved pre-edge feature at ∼5718 eV, without interference of the Cs L_I_ absorption edge present in the conventional XANES measurement. Unfortunately, the better-resolved features require many more fit parameters when attempting to fit these data, resulting in strong correlations between the parameters that render the fit results unreliable. In any case, the peak area of this lower-energy peak appears to be similar between samples, consistent with the measured *n*(f^0^) values obtained within error from conventional XANES, assuming that the feature arises from mixed d- and f-states.^43^ With the improved resolution, the “middle peak” feature observed in the oxo XANES spectra is also better resolved. The ability for FDMNES^45^ simulations to reproduce this feature (discussed below) provides support that this feature is not due to a separate configuration but rather to ligand field splitting. This splitting is also observed in the imido and anilide (Fig. S17†) spectra, but to a lesser extent than with the oxos. The imido and anilide spectra appear similar to each other. The only observable difference is a slightly greater splitting of the f^1,2^ peak in 1-H compared with the imidos, in addition to a higher f^0^ peak, consistent with XANES. The trends observed in the intensity of the pre-edge peak correlate with those observed in f^1,2^ peak splitting, with the imidos having the least intense pre-edge and the oxos the most intense feature. As with XANES, alkali-metal capping of the cation species appears not to appreciably affect the imido spectra. The same is not true for the oxos. Instead, a trend can now be distinguished as a function of cation with increasing Z. The “middle peak” feature appears most intense in (2-Cs)_4_, followed by (2-Rb)_4_, then (2-K)_4_. This trend suggests that the capping cation species can affect the electronic structure of the complexes. This hypothesis was further explored using FDMNES simulations.

**Fig. 4:**
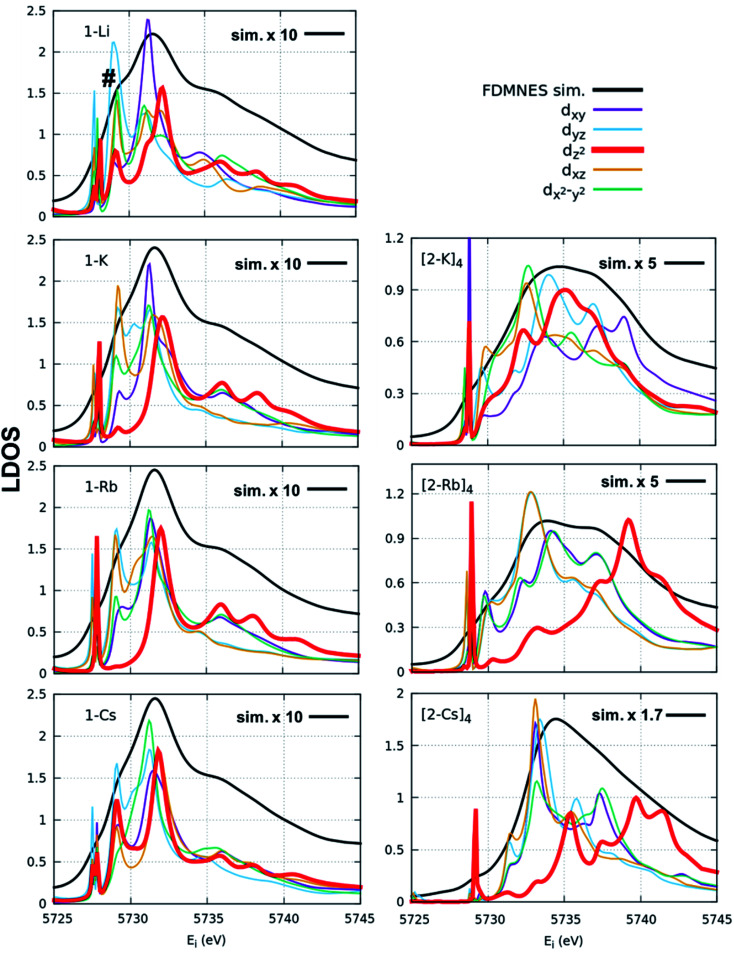
FDMNES simulations of Ce(iv) imido (left) and oxo (right) local density of states (LDOS). Note that only the f^1^ configuration is represented by the simulation. Different d-state contributions are plotted in different colors. The # symbol in the 1-Li panel designates the lower energy shoulder most pronounced in this sample. The d_*z*^2^_ contribution within the oxo samples (red) is in bold to emphasize the differences in d-state splitting depending on cation capping species.

FDMNES simulations of the local density of states (LDOS) provide insight into d-state splitting observed from HERFD-XAS. While FDMNES is limited to features due to the f^1^ configuration as only one configuration can be simulated, the individual peak shapes are well-reproduced (Fig. 4, S16–S20†). Through simulation, the separate contributions from various d orbitals to the density of states can be observed. From HERFD-XAS, a trend is visible in d-state broadening from the imidos (narrowest) to anilide to oxos (broadest). In the imidos, differences in d-state distributions are observed between varying alkali metal capping cations. Specifically, a lower energy shoulder, marked with # in Fig. 4, is more pronounced in the 1-Li simulation than for the other imidos. In the oxos, the increased d-state splitting observed in the (2-Cs)_4_ measurement compared with those from (2-K)_4_ and (2-Rb)_4_ in the HERFD-XAS can then be attributed to the d_*z*^2^_ contribution splitting off to higher energy. While in (2-K)_4_, all d orbital distributions appear broad and at similar energy, the d_*z*^2^_ contribution to the density of states splits to higher energy for (2-Rb)_4_ and even higher for (2-Cs)_4_.

#### Magnetism

**Fig. 5:**
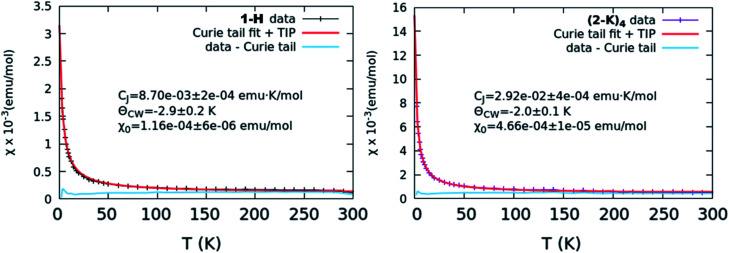
*χ vs. T* curves representative of the lowest level of TIP (left, 1-H) and greatest level of TIP (right, (2-K)_4_). In both cases, the collected curve is shown along with the results from a Curie–Weiss fit as well as the data with the Curie tail subtracted. Other collected curves and fits are shown in Fig. S17.†

**Fig. 6:**
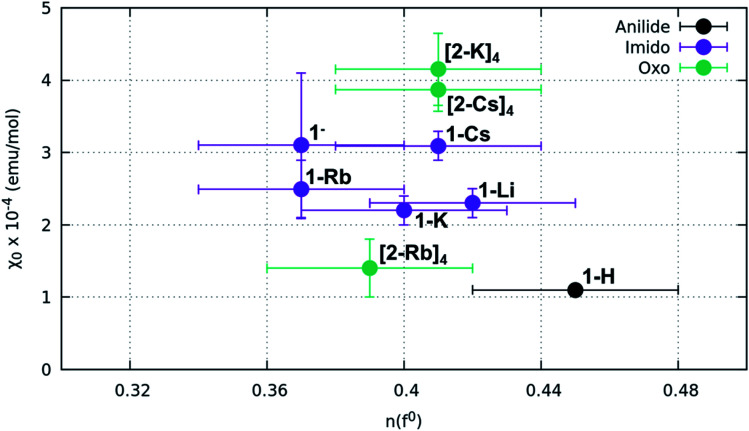
*χ*_0_ values extracted from fitting data from SQUID are plotted as a function of *n*(f^0^) extracted from XANES fitting. No correlation between *n*(f^0^) and *χ*_0_ is observed despite the wide range of *χ*_0_ values observed.

SQUID magnetometry was performed on all samples and the obtained susceptibility *versus* temperature curves were fit using a Curie–Weiss + constant model to extract values for the Curie constant (*C*_J_), the Curie–Weiss temperature (*ϴ*_CW_) and the level of TIP (*χ*_0_) (Fig. 5 and S21,†Table 1). Relatively low *C*_J_ values throughout (for comparison, *C*_5/2_ = 0.807 emu K mol^−1^ assuming unquenched spins) suggest that any paramagnetic impurity in the samples is of a low amount and the data should otherwise be representative of the sample of interest. While accounting for such impurity “Curie tails,” all the complexes exhibit a non-zero *χ*_0_, and hence TIP. Overall, the *χ*_0_ values are lowest for 1-H (1.1 ± 0.06 × 10^−4^ emu mol^−1^) and highest for the oxos (∼4 × 10^−4^ emu mol^−1^), with the exception of (2-Rb)_4_, which is an outlier in this trend. Trends in *χ*_0_ are compared with *n*(f^0^) values obtained through XANES fitting in Fig. 6. These two quantities are expected to be strongly correlated when a multiconfigurational ground state singlet mixes *via* a van Vleck mechanism to a triplet state.^26,28,46^ Results, however, show no perceivable relationship between *χ*_0_ and *n*(f^0^). The results of Evans analysis of ^1^H NMR spectra (see ESI†) confirm the paramagnetic nature of the products and are in agreement with the low TIP levels reported from the SQUID data.

#### Calculations

**Fig. 7:**
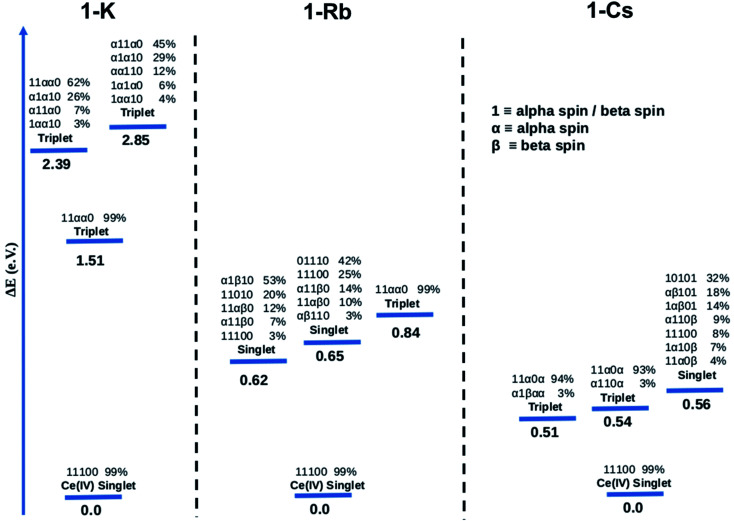
Energy levels and configurations of 1-K (left), 1-Rb (center) and 1-Cs (right) complexes obtained at the CASSCF(6,5) level. 0, α or β and 1 are used for depicting when the occupation of the MO is zero, one or two, respectively. Only configurations ≥ 3% are shown. All ground states represent closed-shell singlets.

**Fig. 8:**
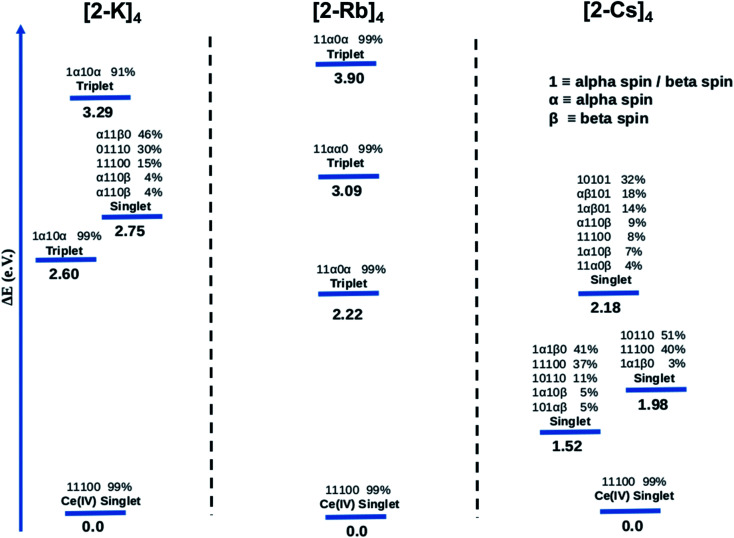
Energy levels and configurations of (2-K)_4_ (left), (2-Rb)_4_ (center) and (2-Cs)_4_ (right) complexes obtained at the CASSCF(6,5) level. 0, α or β and 1 are used for depicting when the occupation of the MO is zero, one or two, respectively. Only the configurations ≥3% are shown. All ground states represent closed-shell singlets.

Fig. 7 summarizes the results obtained from CASSCF calculations for the ground state and the three lowest excited states of the Ce imido complexes, and Fig. 8 summarizes results for the Ce oxo complexes. Within Fig. 7 and 8, 0 means no electrons and 1 means two electrons, for example, in the notation 11 100 for the singlet ground state of 1-K. When the orbital is half-filled, the spin of the electron is given (alpha or beta). Since there are 5 active orbitals, this corresponds to 5 presented digits, where the lowest energy is on the far left and the highest on the far right. The ground electronic state of 1-K is predicted to be a closed-shell singlet despite the fact that the active orbitals were taken from the restricted open shell Hartree–Fock (ROHF) calculation of the spin triplet state (*S* = 1). Interestingly, the two f-type orbitals are empty (see ESI† for more information about the MOs used in these calculations) in line with a Ce(iv)-type complex. These CASSCF calculations predict that the first excited state is a single-reference triplet located 1.51 eV above the ground state. Two multiconfigurational triplet states are also found 2.39 and 2.85 eV above the ground state.

The computed ground state for the 1-Rb is also a single-reference closed-shell spin singlet. However, in this case three excited states, two multiconfigurational singlets and one single-reference triplet are closer in energy to the ground state (0.62, 0.65 and 0.84 eV, respectively) than in 1-K. In the case of the 1-Cs ground state, the CASSCF method predicts a closed-shell spin singlet with three excited states (two nearly single-reference triplets and one multireference singlet) 0.51, 0.54 and 0.56 eV above the ground state.

CASSCF results show single-reference closed-shell singlet ground states for the three alkali metal-capped Ce imido complexes. However, the lack of 4f orbital occupancy in the CAS calculations appears inconsistent with the *n*(f^0^) values determined from XANES (∼0.4). Therefore, the bonding characteristics of the capped imido complexes were further analyzed using Natural Bonding Analysis (NBO) and Wiberg Indexes (WBI), with nearly the same results for all three of these complexes: in each case, a double bond Ce=N was found. Both σ and π bonds are strongly polarized towards nitrogen (82% for σ and 85% for π) and involve a hybrid 5d/4f orbital on Ce. Directly consistent with the XANES-derived results, the 5d/4f mixtures in these bonding orbitals are 60% 5f – 40% 5d. The associated WBI is 1.76 (close to 2.0) indicating that the bonds are significantly covalent. Therefore, in a covalent picture, a large fraction of these bonding electrons could be attributed to the Ce (and mainly on the 4f orbital), explaining the *n*(f^0^) value obtained experimentally. For the sake of completeness, CASSCF calculations were carried out using the same methodology for the uncapped imido complexes. The ground state is found to be a closed-shell singlet, as for all alkali-capped complexes. The first excited states are found to be two open-shell singlets (0.45 and 0.54 eV) and a triplet (0.74 eV).

The CASSCF results for the alkali metal-capped Ce oxo complexes (Fig. 8) are quite similar to those determined for the imido case. For all complexes, we found single-reference ground states where the f-type orbitals included in the CAS are unoccupied. For both (2-K)_4_ and (2-Rb)_4_, the first excited state is a single-reference triplet, whereas for the (2-Cs)_4_ it is a multi-reference singlet. The energy of the second singlet and second triplet of (2-K)_4_ are 2.75 eV and 3.29 eV above the ground state respectively. On the other hand, the next calculated excited electronic states for (2-Rb)_4_ (Fig. 8) are single-reference triplets in which one electron with alpha spin occupies one of the f orbitals of the Ce atom. For (2-Cs)_4_, three singlet excited states located 1.52, 1.98 and 2.18 eV above the singlet ground state have been identified. Following a similar procedure as for the imido complexes, the bonding was analyzed further. At the NBO level, only purely donor–acceptor interactions were observed, where the oxygen acts as a donor and the cerium as an acceptor. The associated WBI for each of the oxos is 1.34, indicating that the Ce=O bonds are less covalent than for the imido cases, and therefore having a stronger ionic component. This is reminiscent of the study by Barros *et al.*^47^ regarding the nature of the bonding in uranium oxo and imido complexes. The acceptor orbitals on Ce are again hybrid 5d/4f orbitals that are based on 60% 4f and 40% 5d, just as with the imido complexes. These results are in line with the experimental observation of similar *n*(f^0^) values between the imido and oxo complexes. CASSCF calculations for an uncapped oxo complex also predict the ground state to be a closed-shell singlet with the first excited states being up to 4.0 eV higher in energy.

## Discussion

The overall results described above are surprising due to (1) the feature between the f^1,2^ peak and f^0^ peak in the L_3_-edge XANES which necessitated more detailed analysis using HERFD; (2) the similarity in *n*(f^0^) between the imido and oxo compounds, irrespective of Ce = N or Ce = O motif or alkali metal capping cation species; and (3) the same lack of variability despite large changes in the degree of temperature-independent paramagnetism. In fact, our initial hypothesis was that the capping cation species might enable tunability in *n*(f^0^) based on changing the degree of covalency; however, this was not observed.

More specifically, the XAS results show that the anilide, imido and oxo samples can all be considered as formal Ce(iv), yet despite the similarities in *n*(f^0^) between the Ce imido and Ce oxo complexes, XANES and HERFD data both exhibit notable differences in the characteristics of the f^1,2^ and f^0^ peaks. HERFD results confirm that these dissimilarities are not attributable to differences in f-orbital occupancy, but rather to ligand-field splitting behavior of the 5d states. This result is interesting in that, while the electronic structure between the two classes of complexes is identical with respect to fractional f^0^ occupancy, the distribution of the d-states differs depending on the Ce=N *vs.* Ce=O bonding species, with the splitting greater in the oxo complexes (Fig. 4). In addition, the d_*z*^2^_ state is notably shifted to higher energy in comparison to the imidos. The greater ligand-field splitting in the oxos is somewhat expected, based on bond length trends, as indicated in Table 1: Ce–L bond lengths (L = ligand) are shortest for the oxos (most splitting) and longer for the imidos (least splitting). Computationally, all alkali metal-capped imido and oxo complexes are determined to have closed-shell singlet ground states with hybrid 5d/4f orbitals that are based on a 60% 4f and 40% 5d mixture, consistent with the *n*(f^0^) values extracted from XANES fitting. As a general trend, however, the energy gap between the ground and lowest-lying excited state is smaller for the imidos than for the oxos. A different degree of covalent behavior is also observed between the classes of complexes. The WBI parameter corresponds to Ce=N double-bonded character for the imidos, suggesting that the bonding is significantly covalent. In contrast, the WBI parameter for the oxo case suggests closer to single-bond character with a greater ionic component. Despite the different covalent character, similar *n*(f^0^) values result from two different mechanisms. In the imidos, a fraction of the bonding electrons can be attributed to Ce on the 4f orbital, leading to a 5d/4f mixture in the bonding orbitals of 60% 4f and 40% 5d. In the oxos, only pure donor–acceptor behavior is observed, where Ce is the acceptor, with hybrid 5d/4f orbitals also with 60% 4f and 40% 5d. This observation provides an important example of how complexes with significantly different bonding characteristics can still result in similar *n*(f^0^) values.

We consider also the effects of varying the alkali-metal capping cation species from harder (Li^+^, K^+^) to softer (Cs^+^) on the resulting electronic structure of the Ce imido and oxo complexes. Capping-cation-dependent trends observed for the Ce imido complexes are subtle, and best perceived through FDMNES simulations (Fig. 4). The d-state distribution narrows as noted from a lower-energy shoulder going from Li^+^ to Cs^+^. The opposite trend is observed in the oxo complexes, where d-state splitting is enhanced, resulting in distribution broadening going from K^+^ to Cs^+^. This trend is visible in experimental HERFD data (Fig. 3) and explained through FDMNES simulations (Fig. 4) as resulting from the out-of-plane d-states (d_*z*^2^_) shifting to higher energies. The uncapped imido 1^−^ was synthesized and exhibits very similar spectroscopic behavior to 1-Cs. Computational results provide additional information regarding the excited states of the complexes, and how they are affected by the capping alkali metal cation. As a general trend, for both the imidos and oxos, the energy gap between the ground state and lowest-lying excited state is largest for K^+^ and lowest for Cs^+^ capping species. With the exception of (2-Rb)_4_, all complexes feature multireference excited states within the three lowest energy states, but the nature of these excited states varies from complex to complex in an apparently non-systematic manner. Together, the data suggest that the alkali metal capping species, while having no perceivable effect on *n*(f^0^), have the capability to fine-tune the d-state and excited state distributions within the complexes. Moving forward, it would be interesting to consider how this might be used as a tool to provide finer control over complex reactivity.

Results from our magnetism study as well as CASSCF calculations indicate that the electronic picture within the Ce imido/oxo systems is even more complex than the systematic trends observed from XANES and HERFD suggest. All complexes exhibit enhanced levels of TIP; however, a number of anomalies suggest that TIP is not driven by a simple van Vleck mixing of the ground state singlet and excited state triplet states involving localized f- and ligand orbitals as is believed to be the case for some other lanthanide organometallic systems.^7–9,11–14^ For one, in contrast to previous work on Ln coordination complexes where multiconfigurational, open-shell singlet ground states exist, all these closed-shell Ce imido and oxo complexes exhibit a large difference in *χ*_0_ despite having similar *n*(f^0^) values. In fact, it should be noted that the *χ*_0_ values represent a wider range than has been reported previously even for more structurally diverse formal Ce(iv) complexes,^46^ although the amount of available literature data remain limited. Additionally, no observed correlation exists between the ground state and the first excited state triplet determined from CASSCF results and *χ*_0_, as would be expected for a conventional van Vleck mechanism for TIP. Moreover, while the imidos exhibit generally lower levels of TIP than the oxos, (2-Rb)_4_ is a reproducible outlier in this trend. Lastly, within the imido and oxo species with varying alkali metal capping cation, there is no systematic trend from harder to softer cation species regarding the level of TIP observed. While some form of van Vleck mixing must occur to generate TIP behavior, some additional mechanism must be at play than those discussed here to result in the high *χ*_0_ values observed. Future work is needed to rationalize the TIP behavior and the lack of energetically close magnetic excited states from calculations.

An interesting factor that should affect the overall magnetic behavior is that the 5d mixing with the 4f states changes the degree of angular momentum quenching through a combination of the degree of d character and possible variations in the ensuing degree of delocalization of the f-orbital. Measuring the degree of delocalization of Ce 4f orbitals is very challenging. It has been previously noted that a small shift in the white line of the Ce L_III_-edge between the f^1,2^ peak in CeO_2_ and Ce(iii) compounds is due to a more delocalized configuration of the 4f orbital in CeO_2_.^48^ Recent calculations suggest this shift may instead be due to increased f^2^ character when intermediate valence increases, which has a similar effect to a delocalized configuration.^18^ In any case, similar shifts are observed in other formal Ce(iv) compounds,^1^ with cerocene having the closest correspondence to the leading f^1,2^ peak of a pure Ce(iii) compound.^2^ In fact, the f^1,2^ peak energy is shifted from the position of pure Ce(iii) (Table S2†), indicative of delocalized behavior. Notably, the f^1,2^ peak is not shifted as far as in CeO_2_, suggesting that the 4f orbital in either the imido and oxo samples presented in this study is not as delocalized. However, there are no notable differences between the imido and oxo samples regarding the f^1,2^ peak from XANES, the lack of which suggests that if this effect plays a role in the variations in *χ*_0_ for the present compounds, the ensuing edge shifts are within the resolution of the data.

CASSCF calculations provide some additional insight into the non-systematic differences observed in the magnetism data for the imido and oxo complexes. We find that the distribution and nature of these excited states varies from complex to complex. These variations could, in part, explain the lack of trends between the complexes in magnetism data. Although CASSCF results show trends in ground-to-excited state gaps that reflect periodic trends in the alkali metal cations, related trends are not apparent in the measured TIP values. It is also worth considering why (2-Rb)_4_ remains an outlier with respect to its low level of TIP in comparison to the other oxo complexes. While this exception does not mimic any of the trends observed from our spectroscopic measurements, there is precedence for magnetic behavior to follow non-systematic trends based on associated alkali metal species.^3^ We can also consider anomalies in the nature of the excited states of the complexes. In particular, what differs for (2-Rb)_4_ compared with the other complexes is both the lack of multireference excited states and the large energy differences between its excited states. Each of the three lowest-lying triplets is fairly discrete in energy (Fig. 8), rather than clustering around a similar energy range as is apparent especially for the imido samples (Fig. 7). Regardless of the exact reason for the anomaly in magnetism trends, it is apparent that the electronic structure of these complexes is complex, and beyond the predictability of periodic trends.

## Conclusion

We performed electronic structure studies on previously reported Ce(iv) imido and oxo compounds, as well as two new Ce(iv) oxo species. The electronic behavior of the Ce imido and oxo complexes presented herein contrasts that of Ln organometallics in which delocalized electrons from aromatic groups are directly interacting with the Ln center, and as a result exhibit multiconfigurational ground states. Rather, by separating the Ln center from the aromatic groups and stabilizing Ce=N or Ce=O moieties through use of an alkali metal capping cation, the ground states are not multiconfigurational, but rather closed-shell singlet single-reference ground states. We observe that these closed-shell singlet systems are fundamentally different than those with open-shell singlet ground states due to the involvement of the 5d orbital, which we track in detail using XANES and HERFD-XAS. The 4f/5d hybridization within these systems is strong and is what gives rise to the double-peak feature observed in the resulting XANES spectra. Our spectroscopy results confirm that despite having *n*(f^0^) values that are essentially the same across the complexes, the energy distribution of d-states within the complexes varies substantially. This distribution is also more subtly affected by the identity of the alkali metal capping cation. While previous studies have shown that small changes in molecular perturbation can result in large f-orbital occupancy changes in lanthanide organometallics, this study shows that small changes in molecular structure can result in relatively fixed f-orbital occupancy, with different distributions of underlying states, potentially driving large differences in their paramagnetic states. Moving forward, such complexes provide a platform to consider in future studies how fine-tuning d-state distributions within Ln organometallic complexes such as those reported herein can affect reactivity in a manner that is independent of lanthanide valence.

## Experimental

### General synthesis methods

All reactions and manipulations were performed under an inert atmosphere (N_2_) using standard Schlenk techniques or in a drybox equipped with a molecular sieves 13X/Q5 Cu-0226S catalyst purifier system. Glassware was oven-dried for at least 3 h at 150 °C prior to use. ^1^H NMR spectra were obtained on a Bruker DMX-300 Fourier transform NMR spectrometer operating at 300 MHz for ^1^H, 75.48 MHz for ^13^C and 282.2 MHz for ^19^F. Chemical shifts were recorded in units of parts per million and referenced against residual proteo solvent peaks. Infrared spectra were measured on a PerkinElmer 1600 series spectrometer. UV-vis spectra were recorded on PerkinElmer Lamba 950 spectrometer. Infrared spectra were recorded in the range from 4000 to 500 cm^−1^ on Bruker Invenio R spectrometer in KBr pellets. Elemental analyses were recorded on a Costech ECS 4010 analyzer. Cyclic Voltammetry (CV) experiments were performed using a CH-Instruments 620D Electrochemical Analyzer/Workstation. Data were processed using CHI software v9.24. All experiments were performed in an N_2_ atmosphere glove-box using electrochemical cells consisting of a 4 mL vial, glassy carbon working electrode, a platinum wire counter electrode, and a silver wire plated with AgCl as a quasi-reference electrode. The working electrode surfaces were polished prior to each set of experiments. THF solutions of the analyzed compound (∼1 mM) and supporting electrolyte [^*n*^Pr_4_N][BArF_24_] (0.1 M) were used for electrochemical studies. Potentials were reported *versus* the ferrocene/ferrocenium (Fc/Fc^+^) couple. Ferrocene was added as an internal standard for calibration at the end of each run. All data were collected in a positive-feedback IR compensation mode. THF and pentane were sparged for 20 min with dry argon and dried using a commercial two-column solvent purification system comprising columns packed with Q5 reactant and neutral alumina respectively (for pentane and hexanes). Deuterated toluene and benzene (Cambridge Isotopes) were stored over molecular sieves (4 Å) overnight prior to use. THF-*d*_8_ (Cambridge Isotopes) was dried over sodium and distilled prior to use. Benzophenone (ACROS organics) was recrystallized from ethanol and vacuum-dried prior to use. Potassium bis(trimethylsilyl)amide (Sigma) was used as received. Cesium bis(trimethylsilyl)amide was prepared according to published reports.^49^

#### Synthesis of (2-K)_4_

To a solution of Ce(TriNO_*x*_)[NH(3,5-(CF_3_)_2_C_6_H_3_)] (400 mg, 0.438 mmol, 1.0 equiv.) in THF (2 mL) was added solid KN(SiMe_3_)_2_ (88 mg, 0.438 mmol, 1.0 equiv.). After stirring for 10 minutes, solid benzophenone (80 mg, 0.438 mmol, 1.0 equiv.) was added to the resulting purple solution. A yellow solid gradually precipitated from the solution upon stirring. After 2 h, the solid product was collected by decantation and subsequently washed with an additional portion of THF (2 mL). The product was then dried under vacuum to give analytically pure (2-K)_4_. Yield: 187 mg, 58%. ^1^H NMR (400 MHz, 298 K, THF-*d*_8_): δ 7.91 (d, ^2^*J*_HH_ = 7.8 Hz, 3H, Ar*H*), 7.23–7.19 (t, ^3^*J*_HH_ = 7.52 Hz, ∼3H, Ar*H*), 7.0 (m, ∼6H, Ar*H*), 4.16 (d, ^2^*J*_HH_ = 10.2 Hz, 3H, C*H*_2_), 2.23 (d, ^2^*J*_HH_ = 11.7 Hz, 3H, C*H*_2_), 1.0 (s, 27H, C(C*H*_3_)_3_). ^13^C NMR (126 MHz, THF-*d*_8_) 150.5 (s, Ar), 130.9 (s, Ar), 129.7 (s, Ar), 127.9 (s, Ar), 125.3 (s, Ar), 122.4 (s, Ar), 59.2 (s, *C*(CH_3_)_3_), 58.2 (s, *C*H_2_), 24.7 (s, C(*C*H_3_)_3_). Anal calc'd for (Ce_1_K_1_O_4_C_33_H_45_)_4_: C, 53.49; H, 6.12; N, 7.56. Found: C, 52.99; H, 5.86; N, 7.58.

#### Synthesis of (2-Cs)_4_

To a solution of Ce(TriNO_*x*_)[NH(3,5-(CF_3_)_2_C_6_H_3_)]^7^ (300 mg, 0.33 mmol, 1.0 equiv.) in THF (2 mL) was added solid CsN(SiMe_3_)_2_ (96.7 mg, 0.33 mmol, 1.0 equiv.). After stirring for 10 minutes, solid benzophenone (60 mg, 0.33 mmol, 1.0 equiv.) was added to the resulting purple solution. A yellow solid gradually precipitated from the solution upon stirring. After 2 h, the solid product was collected by decantation and subsequently washed with an additional portion of THF (2 mL). The product was then dried under vacuum to give analytically pure (2-Cs)_4_. Yield: 327 mg, 39%. ^1^H NMR (400 MHz, 298 K, THF-*d*_8_): *δ* 7.91 (d, ^2^*J*_HH_ = 8 Hz, 3H, Ar*H*), 7.27–7.24 (t, ^3^*J*_HH_ = 7.6 Hz, ∼3H, Ar*H*), 7.1–7.0 (m, ∼6H, Ar*H*), 4.17 (d, ^2^*J*_HH_ = 11 Hz, 3H, C*H*_2_), 2.26 (d, ^2^*J*_HH_ = 11.3 Hz, 3H, C*H*_2_), 1.0 (s, 27H, C(C*H*_3_)_3_). ^13^C NMR (126 MHz, THF-*d*_8_): 151.4 (s, Ar), 131.9 (s, Ar), 131.1 (s, Ar), 127.6 (s, Ar), 125.3 (s, Ar), 122.3 (s, Ar), 59.5 (s, *C*(CH_3_)_3_), 57.8 (s, *C*H_2_), 24.8 (s, C(*C*H_3_)_3_). Anal calc'd for (Ce_11_Cs_1_O_4_C_33_H_45_)_4_: C, 47.48; H, 5.43; N, 6.71. Found: C, 47.36; H, 5.28; N, 6.53.

#### X-ray crystallography

X-ray intensity data were collected on a Bruker APEX II CCD area detector employing graphite-monochromated Mo-K_α_ radiation (*λ* = 0.71073 Å) at 100(1) K. Rotation frames were integrated using SAINT,^50^ producing a list of unaveraged *F*^2^ and *σ*(*F*^2^) values which were then passed to the SHELXTL program package^51^ for further processing and structure solution. The intensity data were corrected for Lorentz and polarization effects and for absorption using SADABS^52^ or TWINABS.^53^ The structure was solved by direct methods – ShelXS-1997.^54^ Refinement was by full-matrix least squares based on *F*^2^ using SHELXL-2014.^55^ Non-hydrogen atoms were refined anisotropically and hydrogen atoms were refined using a riding model.

#### XANES

X-ray absorption near edge structure (XANES) data at the Ce L_III_ absorption edge were collected at the Stanford Synchrotron Radiation Lightsource (SSRL) beamline 11-2 using a Si(220) (*φ* = 0°) monochromator detuned to 50% and a Rh-coated harmonic rejection mirror with a cutoff energy set near 10 keV. The vertical slit height was sufficiently narrow that the energy resolution was core–hole lifetime limited. Data were collected in transmission geometry and the monochromator energy was calibrated by defining the energy of the first inflection point at the Ce L_III_ edge of the absorption from a CeO_2_ standard to be 5724.0 eV. Data were processed by subtracting a linear pre-edge background and normalizing the edge step to one.

Powder samples were prepared in an argon-filled drybox for measurement by mixing with dry boron nitride and packed into a slotted aluminum holder with aluminized mylar windows, sealed with crushed indium wire. Since the samples are air sensitive, the sealed holders were kept under argon until measurement, and exposed to air for less than one minute during transfer to vacuum. Samples were measured both at 50 K and 300 K using a liquid-helium cooled cryostat to test for temperature dependence of the resulting spectra. An easily-oxidizable sample, such as cerium tris(tetramethylcyclopentadienyl) (CeCp^tet^_3_), was measured along with the samples to ensure that no O_2_ had leaked into the sample holder during measurement.

XANES data were fit in order to extract information about the f-orbital occupancy according to previously described methods,^11,12,14^ although we report the f^0^-configuration fraction, *n*(f^0^), rather than the f-orbital occupancy for reasons that are described in the Results section. Details are provided in ESI.† In particular, the major peaks at approximately 5726 eV and at 5736 eV, which are predominantly due to 2p_3/2_ → 5d_5/2_ transitions that have been split in the presence of the core hole due to the majority f^1^ (with minority f^2^) or the f^0^ configuration fraction, respectively, have been modeled with a single Gaussian function each, even when other contributions are visible. This methodology was chosen so that the data from all the samples could be fit with the same model. Consideration of using a more a complex model is provided in the ESI.† Reported errors include uncertainty from this issue.

#### HERFD

Ce L_III_ edge high-energy-resolution fluorescence detection (HERFD) X-ray absorption spectroscopy measurements were collected at beamline 6-2 of SSRL using a 7-crystal Johann type spectrometer^56^ with Ge(331) analyzer crystals and a Si(311) monochromator. The monochromator energy was calibrated by setting the first infection point of a Ce L_III_ edge CeO_2_ spectrum equal to 5724.0 eV. The beam size was confined to a maximum vertical slit size of 200 μm and a horizontal slit size of 400 μm. HERFD spectra were collected at the maximum intensity of the Ce L_α_ emission line (4840.2 eV) at 50 K and 200 K to test for spectral temperature dependence. Sample preparation and cooling methods were identical to those used for XANES (see above). As with the XANES experiments, CeCp^tet^_3_ was measured along with the samples to confirm that O_2_ had not leaked into the sample holder. Data were processed by subtracting a constant pre-edge background and normalized.

HERFD spectra were simulated using the FDMNES code.^45^ A convolution over the density of states was calculated for a cluster with a 7 Å radius in multiple scattering mode using a Green's function method on a muffin-tin potential. The partial projection of the density of states, including specific d-state contributions, were extracted. The core level width was reduced to 1.2 eV in order to obtain simulations comparable to the experimental HERFD data. For meaningful orbital contributions, the *z*-axes of the structures were aligned to be along the Ce=N or Ce=O bond in the complex structure. Throughout, orbitals are listed according to FDMNES convention, where (l,*m*) = (2,−2) corresponds to d_*xy*_, (2,−1) to d_*yz*_, (2,0) to d_*z*^2^_, (2,1) to d_*xz*_ and (2,2) to d_*x*^2^−*y*^2^_.

#### Magnetism

Magnetic susceptibility measurements were collected using a liquid-helium cooled 7 T Quantum Design Magnetic Properties Measurement System that uses a superconducting quantum interference device (SQUID). To prepare air-sensitive samples for measurement, samples were sandwiched in a quartz tube between two pieces of quartz wool and sealed under argon. Quartz wool and tubes were baked above 200 °C before introduction into a drybox for sample loading to remove any residual organic material. Pure quartz wool was measured (*χ*_0_ = (−3.7 ± 0.5) × 10^−7^ emu g^−1^) and its contribution subtracted as background depending on the amount present in the sample by mass. Data were collected at 5 and 40 kG over a temperature range from 2–300 K and a two-field correction applied in order to remove any ferromagnetic impurity as previously described.^12^ Ferromagnetic impurities were minimal (≤1 × 10^−5^ emu) and calculated to be <0.01% the moment of magnetite. Diamagnetic corrections were applied to the data using Pascal's constants.^57^

TIP values were extracted by fitting the background-subtracted and two-field corrected data to a constant + Curie–Weiss model with *χ* = *C*_J_/(*T* − *ϴ*_CW_) + *χ*_0_, where *C*_J_ is the Curie constant, *ϴ*_CW_ is the Curie–Weiss temperature indicating the strength of magnetic interactions, and *χ*_0_ is the magnitude of the TIP component to the susceptibility. The Curie–Weiss part of the model is used primarily to subtract small paramagnetic impurity contributions which are generally present for low-susceptibility samples. Multiple measurements per sample were collected to ensure reproducibility. Error bars in the reported *χ*_0_ results were determined by averaging over multiple measurements and samples, and are typically larger than the systematic ∼10–15% error from the quartz-wool background subtraction combined with diamagnetic correction.

#### Measurement of solution magnetic properties

Solution magnetic properties were measured at ambient temperature using Evans method. The analyte solution in THF-d8 was prepared in a J-young tube. A glass capillary with the standard: 1,3,5-tris(trifluoromethyl)benzene was placed in the J-young tube. Then, 5 μL of 1,3,5-tris(trifluoromethyl)benzene was added to the analyte solution *via* micro syringe. The observed difference of the chemical shifts of the standard peaks *Δ*_ppm_ (ppm) on ^1^H NMR spectra was recorded. The solution molar magnetic susceptibility *χ*_m_ (emu mol^−1^) was calculated using the equation:
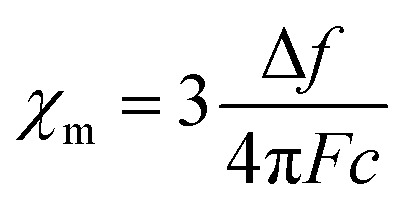
where *c* is the analyte concentration in mol ml^−1^, *F* is the measurement frequency in Hz, and Δ*f* is the difference between the standard peak shifts in solution and in the capillary in Hz. Δ*f* was calculated using the equation:
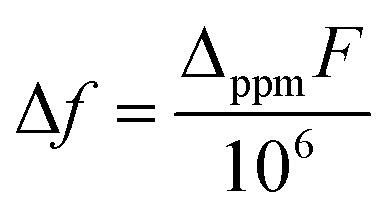


#### Computational studies

The electronic states of Ce imido and oxo complexes have been carefully analyzed through a systematic CASSCF study. We have followed the procedure reported in our previous studies.^12,58^ In order to obtain the optimal geometries of Ce imido and oxo complexes, electronic structure calculations were carried out at the DFT level using the B3PW91 hybrid functional. The optimal geometries of all compounds have been obtained without symmetry restrictions using the hybrid B3PW91 functional. Double-zeta basis set with polarization function (6-31G(d,p)) was used for the hydrogen, carbon, nitrogen and fluorine atoms, while the alkali-metals and cerium atoms were treated with Relativistic Effective Core Potentials (RECP) from the Stuttgart–Köln group.^59^ Cartesian coordinates of all complexes are reported in the ESI.† Due to the importance of covalency in lanthanide complexes, we have explored the nature of the M–Ce (where M = K, Rb, Cs) bonding through the Wiberg bond indices. Indices were computed using the natural bond orbital (NBO) scheme.^60^ CASSCF calculations have been performed distributing six electrons in five active orbitals. Note that these active orbitals were selected from the molecular orbitals (MO's) sets obtained at Restricted Open Shell (ROHF) level. In Fig. S22,† we present the molecular orbitals used to build the complete active space (CAS) for each Ce imido and oxo complex. All electronic structure calculations reported in this work were carried out using the computational chemistry software Gaussian09.^61^

## Data availability

All experimental data, procedures for data analysis and pertinent data sets are provided in the ESI.†

## Author contributions

Moreau, Lapsheva, Schelter and Booth conceived of the project and designed the experiments. The synthetic work and initial characterization by XRD was performed by Lapsheva, Gau, and Manor. Carroll also assisted with XRD data collection and analysis. Characterization using cyclic voltammetry and Evans method analysis was performed by Lapsheva and Yang. XANES data were collected by Moreau, Qiao, and Booth, with the addition of Sokaras for the HERFD data. Analysis of the XANES and HERFD data was performed by Moreau and Booth. Magnetic data were collected and analyzed by Moreau and Booth. Computational studies were performed by Amaro-Estrada and Maron. Lukens oversaw aspects of the overall interpretation. The manuscript was written by Moreau, Lapsheva, Lukens, Schelter, Maron, and Booth.

## Conflicts of interest

There are no conflicts to declare.

## Supplementary Material

SC-013-D1SC06623D-s001

SC-013-D1SC06623D-s002
