# Supplementary material for: Electronic structure studies reveal 4f/5d mixing and its effect on bonding characteristics in Ce-imido and -oxo complexes
Source: Chem Sci. 2022 Jan 24;13(6):1759–73. doi: 10.1039/d1sc06623d (PMC8827158; doi:10.1039/d1sc06623d)
Supplement: SC-013-D1SC06623D-s001 [file SC-013-D1SC06623D-s001.pdf]

Electronic Supplementary Material (ESI) for Chemical Science.
This journal is © The Royal Society of Chemistry 2022

**Supporting Information for:**

# Electronic structure studies reveal 4f/5d mixing and its effect on bonding characteristics in Ce-imido and -oxo complexes

Liane M. Moreau,[1] Ekaterina Lapsheva,[2] Jorge I. Amaro-Estrada,[3] Michael R. Gau,[2] Patrick J. Carroll,[2] Brian C. Manor,[2] Yusen Qiao,[1,2] Qiaomu Yang,[2] Wayne W. Lukens,[1] Dimosthenis Sokaras,[4] Eric J. Schelter,[2] Laurent Maron,[3] and Corwin H. Booth[1]

[1] *Chemical Sciences Division, Lawrence Berkeley National Laboratory, Berkeley, CA 94720, United States*

[2] *Department of Chemistry, University of Pennsylvania, Philadelphia, PA 19104, United States*

[3] *LPCNO, UMR 5215, CNRS, INSA, UPS, Université de Toulouse, 31000 Toulouse, France*

[4] *Stanford Synchrotron Radiation Lightsource, SLAC National Accelerator Laboratory, Menlo Park, CA 94025, United States*

---

**Table of Contents:**

**Figure S1.** Monomeric unit of **(2-Cs)$_4$** ........ S3

**Synthetic details and characterization** ........ S4

*Data for (2-K)$_4$* ........ S4

**Figure S2.** $^{1}$H NMR spectrum for **(2-K)$_4$** ........ S4

**Figure S3.** $^{13}$C NMR spectrum for **(2-K)$_4$** ........ S4

**Figure S4.** UV-Vis spectrum for **(2-K)$_4$** ........ S5

**Figure S5.** IR spectrum for **(2-K)$_4$** ........ S5

**Figure S6.** Cyclic voltammogram for **(2-K)$_4$** ........ S6

*Data for (2-Cs)$_4$* ........ S7

**Figure S7.** $^{1}$H NMR spectrum for **(2-Cs)$_4$** ........ S7

**Figure S8.** $^{13}$C NMR spectrum for **(2-Cs)$_4$** ........ S8

**Figure S9.** UV-Vis spectrum for **(2-Cs)$_4$** ........ S8

**Figure S10.** IR spectrum for **(2-Cs)$_4$** ........ S9

**Figure S11.** Cyclic voltammogram for **(2-Cs)$_4$** ........ S9

**Further details of XANES methods and results** ........ S10

***XANES sample holder integrity*** ........ S10

**Figure S12.** Standard XANES spectra ........ **S10**

***Temperature-dependent XANES spectra*** ........ **S11**

**Figure S13.** $L_{III}$ edge XANES spectra at varying temperatures ........ **S11**

**Figure S14.** HERFD spectra of **(2-Cs)$_4$** at varying temperatures ........ **S12**

***XANES fitting methods*** ........ **S12**

***Results from XANES fitting used to determine*** n($f^0$) ........ **S13**

**Figure S15a-e.** Fits of XANES data ........ **S14-S18**

**Table S1.** XANES fitting parameter results ........ **S18**

*XANES fitting using a 3-peak vs. 2-peak model* ........ **S19**

**Figure S16.** Simulation of **(2-K)$_4$** using 3-peak model vs. 2-peak model ........ **S19**

**Table S2.** $f^{1,2}$ and $f^0$ peak energies from XANES data ........ **S20**

**Raw XANES and HERFD data** ........ **S20**

**Table S3.** Anilide XANES ........ **S20**

**Table S4.** Imido XANES (**1-Li**, **1-K**, and **1-Rb**) ........ **S24**

**Table S5.** Imido XANES (**1-Cs** and **1$^-$**) ........ **S28**

**Table S6.** Oxo XANES ........ **S37**

**Table S7.** Anilide HERFD ........ **S45**

**Table S8.** Imido HERFD ........ **S52**

**Table S9.** Oxo HERFD ........ **S60**

**Additional HERFD and FDMNES results** ........ **S69**

**Figure S17. 1-H** HERFD spectrum ........ **S69**

**Figure S18. 1-H** FDMNES simulation and LDOS ........ **S69**

**Figure S19. 1$^-$** HERFD spectrum ........ **S70**

**Figure S20. 1$^-$** FDMNES simulation and LDOS ........ **S70**

**Further details of magnetism results** ........ **S71**

**Figure S21a-i.** Magnetic susceptibility curves and fitting ........ **S72-S80**

**Evans method analysis** ........ **S81**

**Further details of computational results** ........ **S81**

**Figure S22.** Restricted open-shell Hartree-Fock molecular orbitals for (CAS)........................**S81-S82**

***Cartesian coordinates of all optimized structures***...................................................................**S83-S95**

**References**… ..............................................................................................................................**S96**

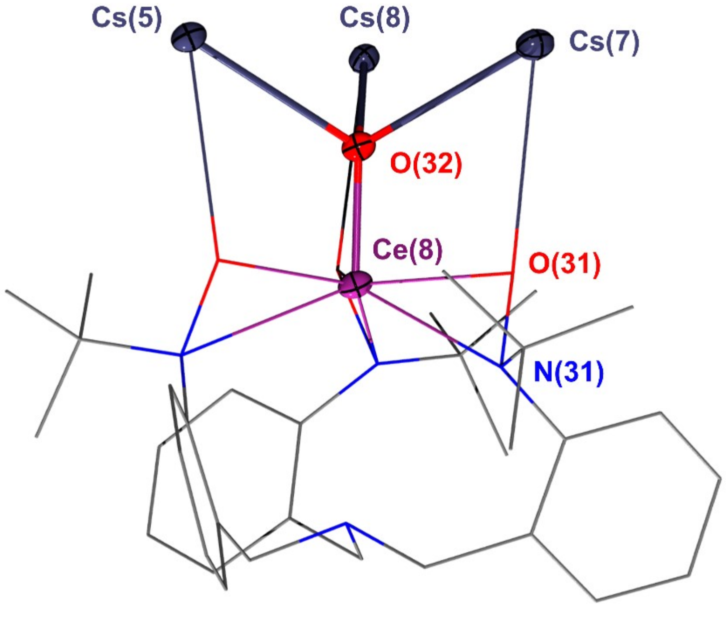


**Figure S1.** A fragment of **(2-Cs)$_4$** structure showing the monomeric [Ce(TriNOx) ═O]$^-$ unit. Thermal ellipsoids are shown at 30% probability. Hydrogen atoms have been omitted for clarity. *Tert*-butyl groups are depicted using the wireframe model.

## Synthetic details and characterization

***Data for (2-K)$_4$***

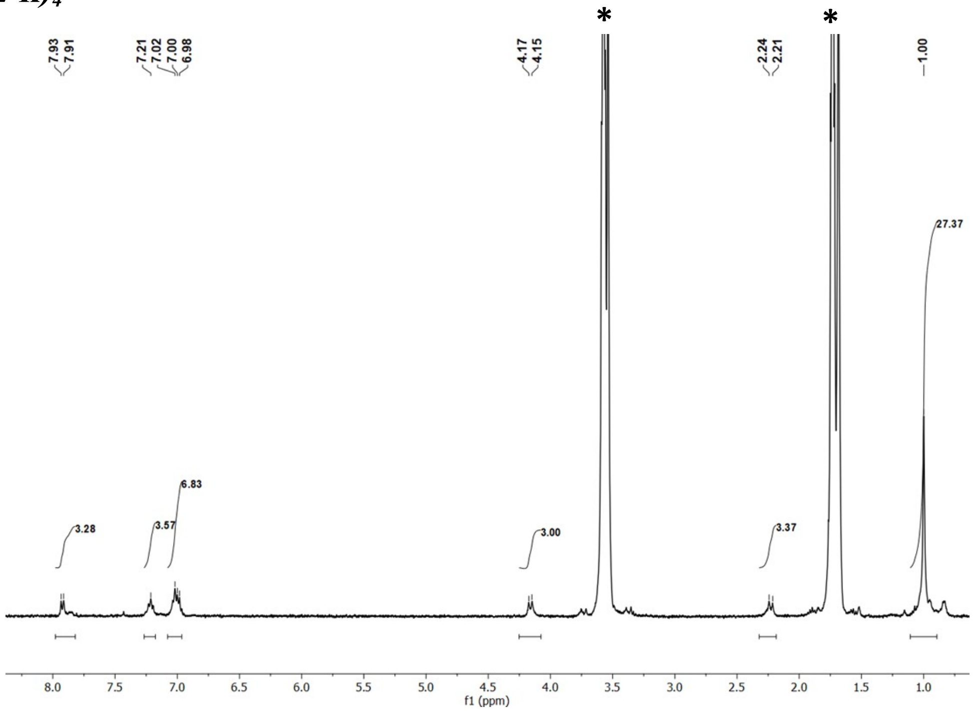


**Figure S2.** $^{1}$H NMR spectrum for **(2-K)$_4$** in THF-*d8*. * - proteo solvent peaks.

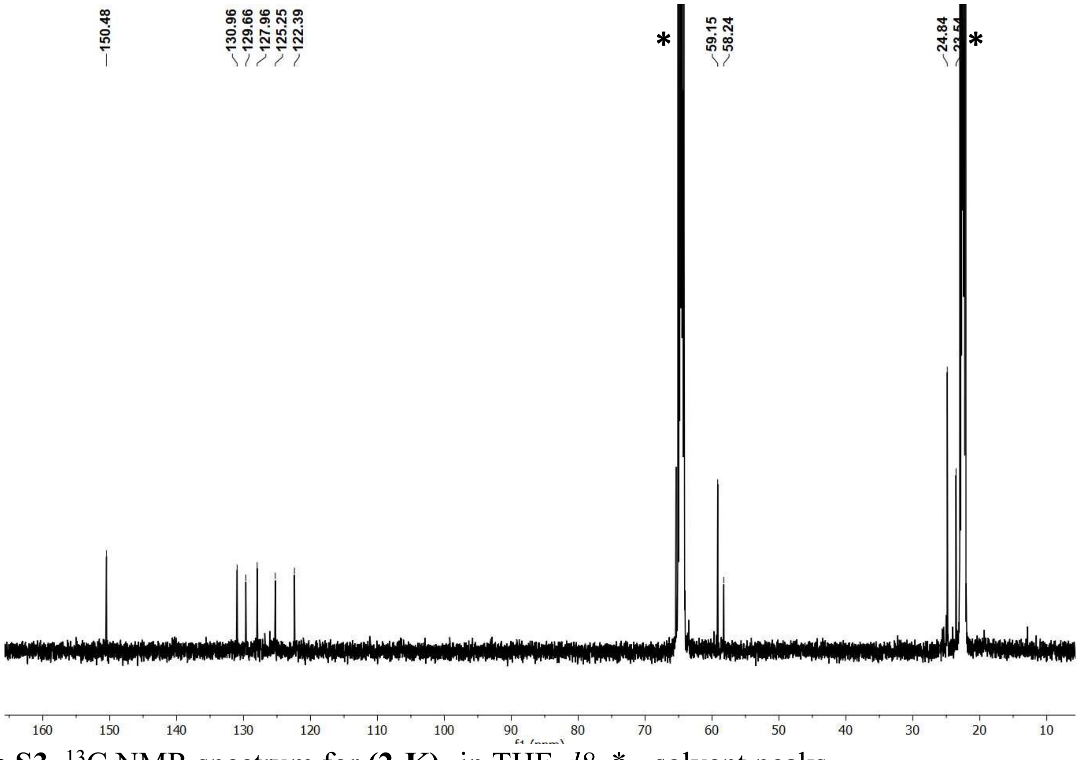


**Figure S3.** $^{13}$C NMR spectrum for **(2-K)$_4$** in THF-*d8*. * - solvent peaks.

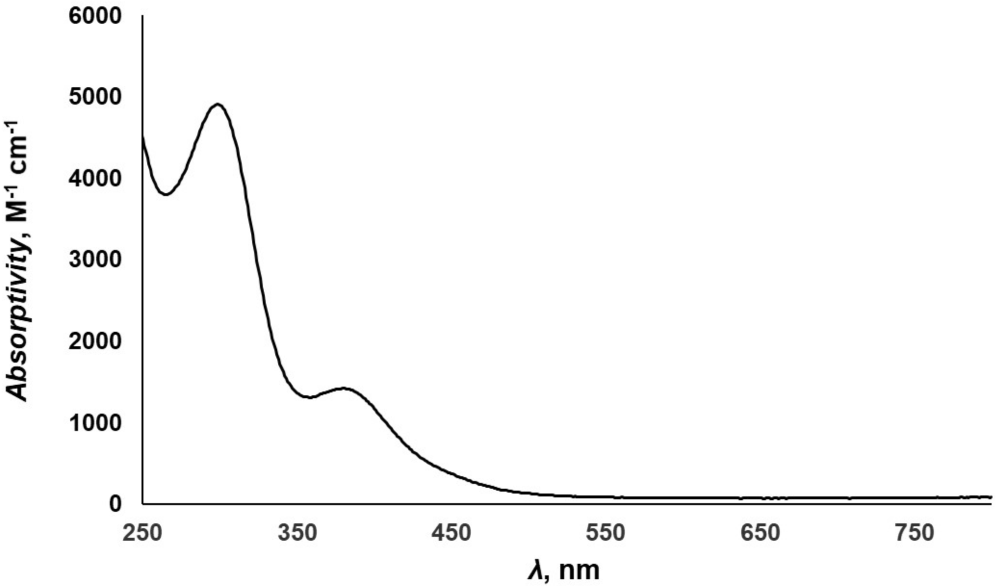


**Figure S4.** UV-Vis spectrum for **(2-K)$_4$** (in THF)**.** $\lambda_{max}$ = 299 nm, 380 nm.

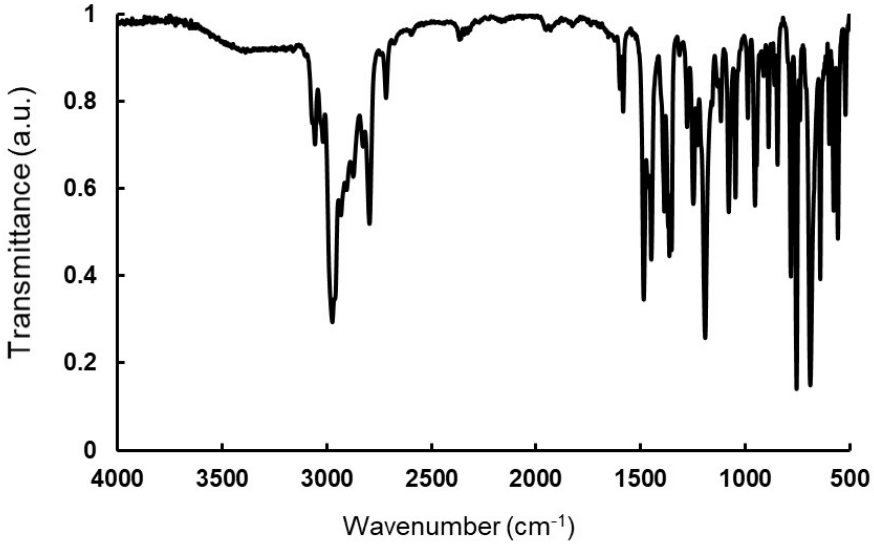


**Figure S5.** IR spectrum for **(2-K)$_4$.**

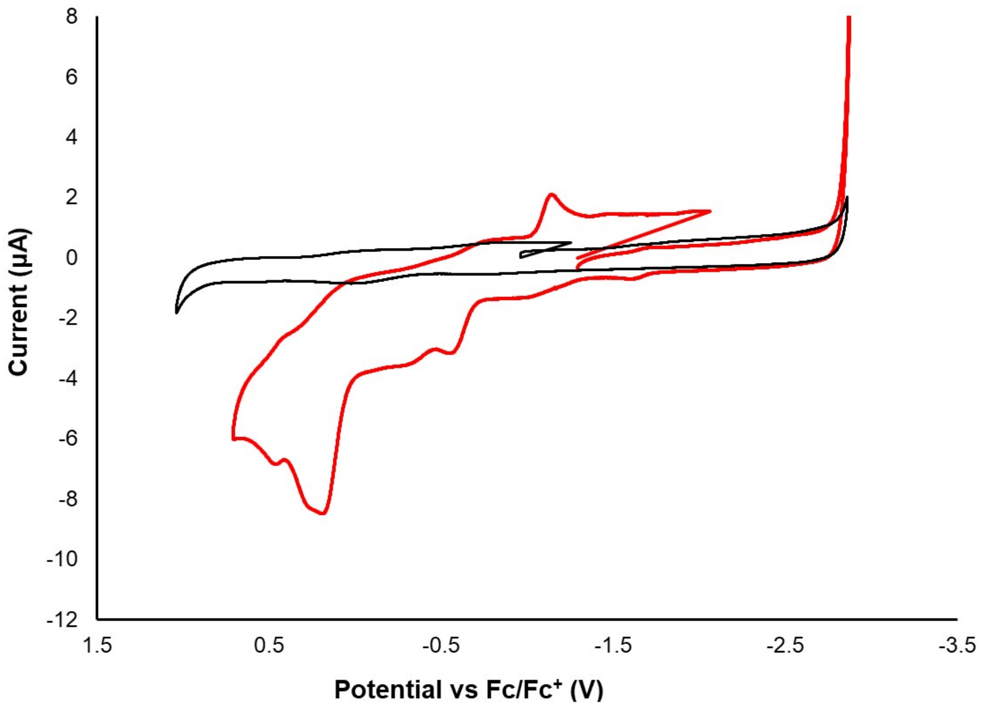


**Figure S6.** Full scan cyclic voltammogram for **(2-K)$_4$** in THF, red trace**.** Scan rate = 100 mV/s, supporting electrolyte [$^n$Pr$_4$N][BArF$_{24}$] (0.1 M). Solvent background, black trace. Experimental details can be found in the main text.

***Data for (2-Cs)$_4$***

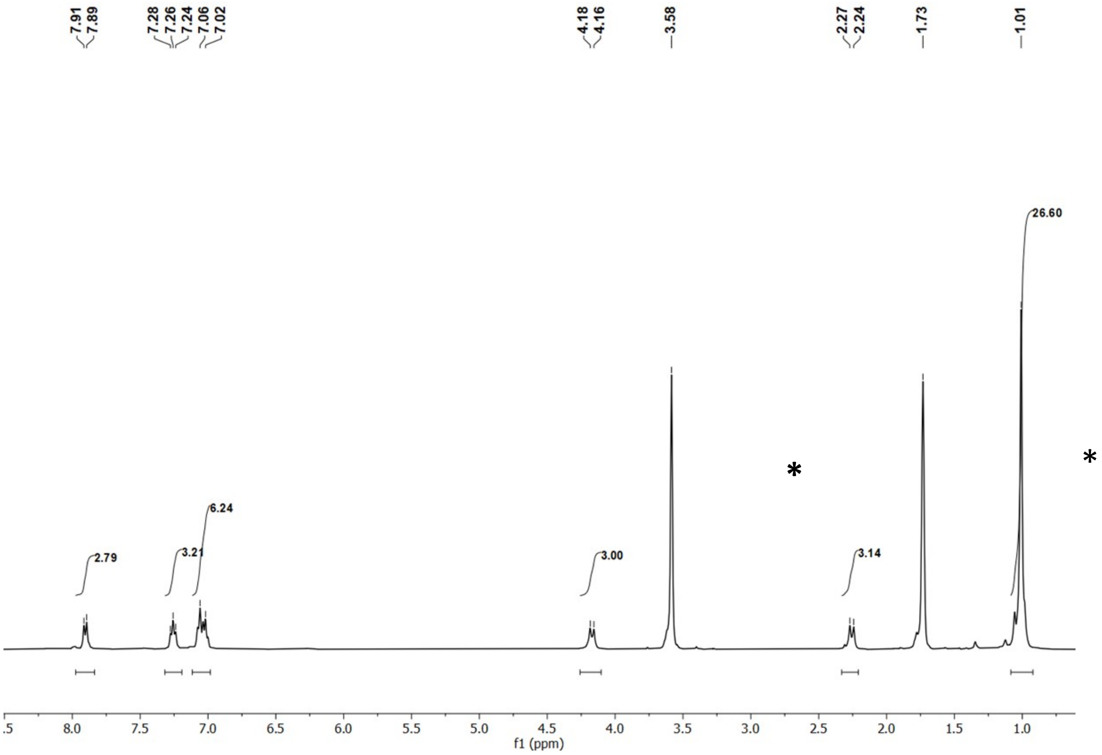


**Figure S7.** $^{1}$H NMR spectrum for **(2-Cs)$_4$** in THF-*d8*. * - proteo solvent peaks.

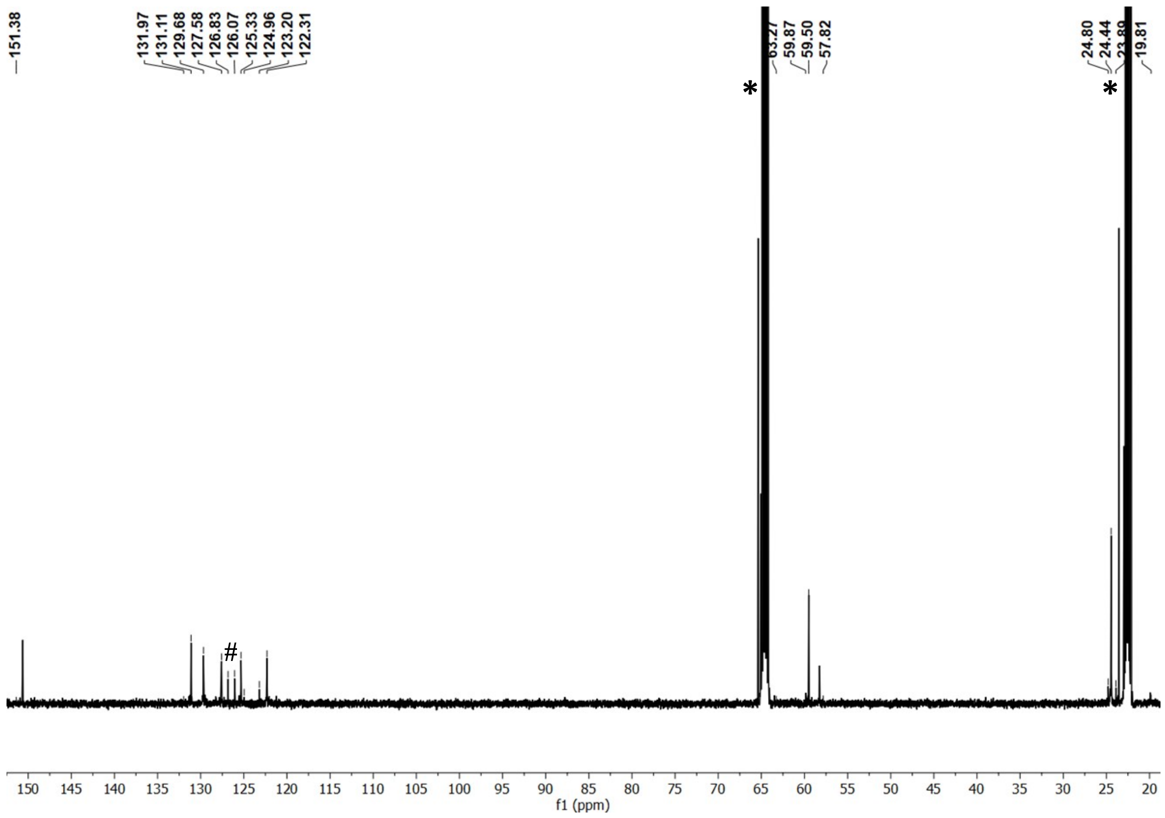


**Figure S8**. $^{13}$C NMR spectrum for **(2-Cs)$_4$** in THF-*d8*. * - solvent peaks. # - solvent impurity peaks.

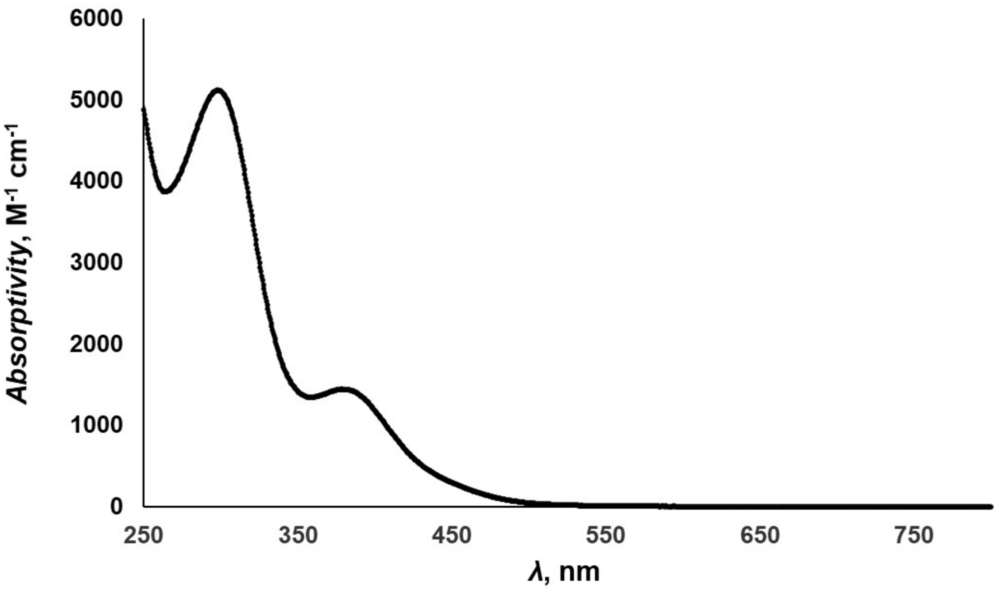


**Figure S9.** UV-Vis spectrum for **(2-Cs)$_4$** (in THF). **$\lambda_{max}$** = 298 nm, 379 nm.

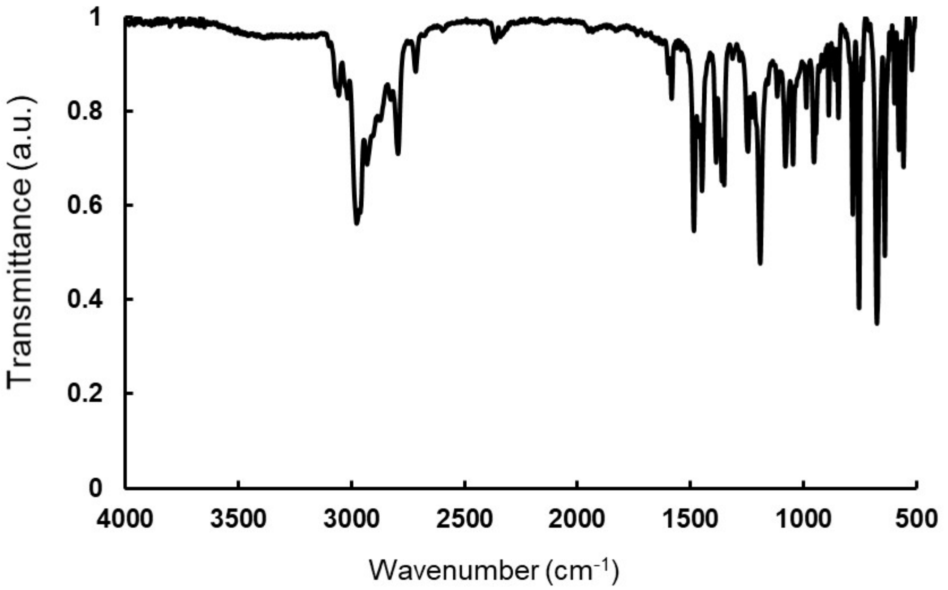


**Figure S10.** IR spectrum for **(2-Cs)$_4$.**

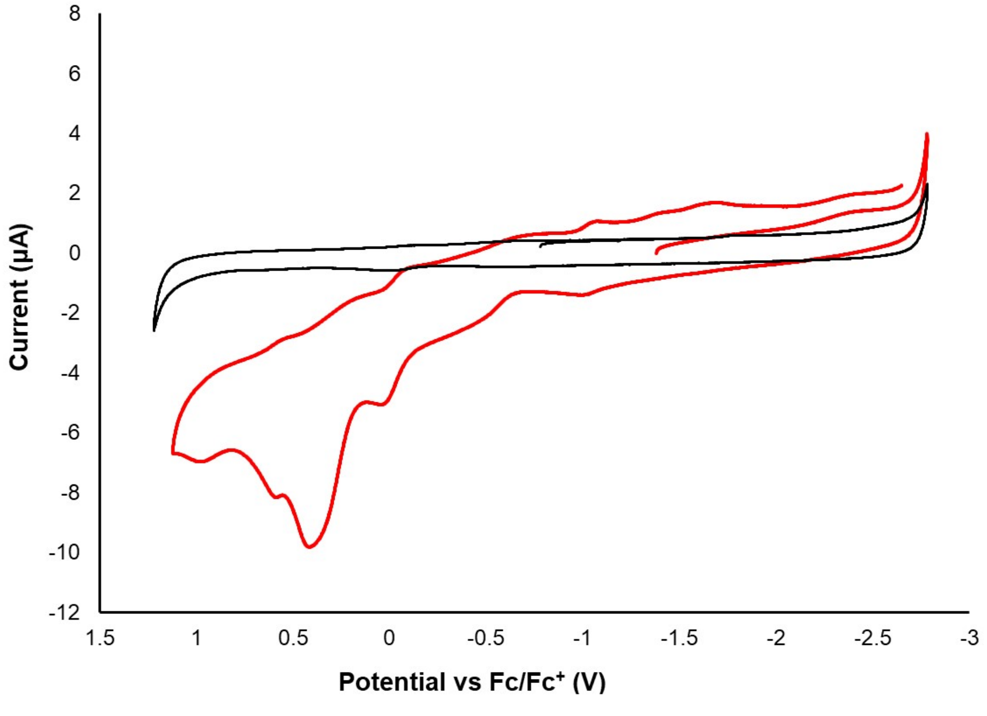


**Figure S11.** Full scan cyclic voltammogram for **(2-Cs)$_4$** in THF, red trace**.** Scan rate = 100 mV/s, supporting electrolyte [$^{n}Pr_4N$][$BArF_{24}$] (0.1 M). Solvent background, black trace. Experimental details can be found in the main text.

## Further details of XANES Methods and Results

### *XANES Sample Holder Integrity*

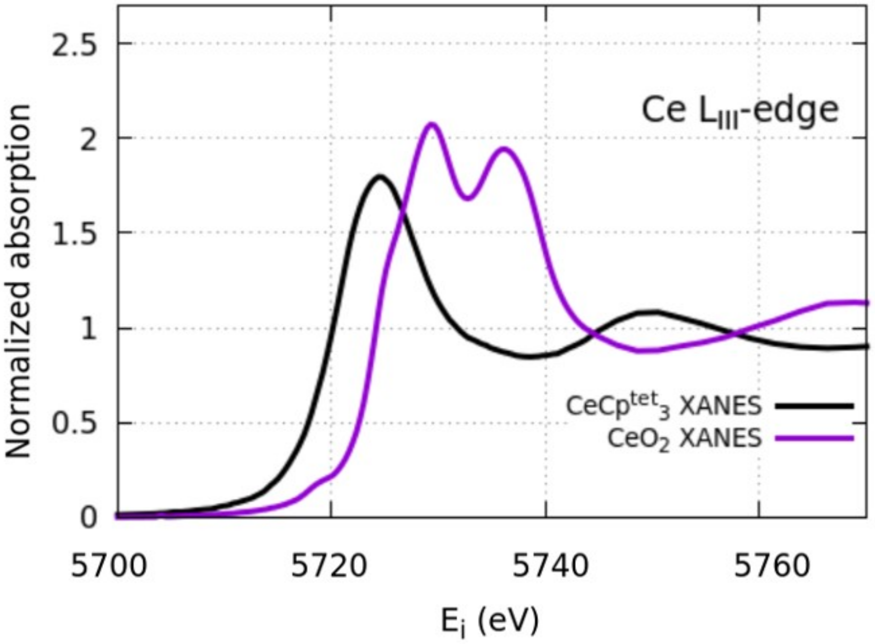


**Figure S12.** Standard XANES spectra. CeCp$^{tet}_3$ (Ce(III)) and $CeO_2$ (Ce(IV)) standards are shown to demonstrate the features expected for formal Ce(III) vs. formal Ce(IV) compounds. CeCp$^{tet}_3$ shows the typical single peak characteristic of Ce(III) and $CeO_2$ shows the double-peak signature characteristic of Ce(IV). CeCp$^{tet}_3$ was used as a canary sample, to ensure that the samples were not compromised by air or water before or during measurement. Namely, if it began to show a second peak characteristic of formal Ce(IV), the data was discarded.

*Temperature-dependent XANES spectra*

From the plots below in Figures S9 and S10, no temperature dependence in the XANES or HERFD spectra was observed, suggesting no changes in electronic structure of the Ce TriNOx complexes as a function of temperature. XANES data were available for all samples except for **(2-Cs)$_4$**, so the HERFD data are instead displayed here.

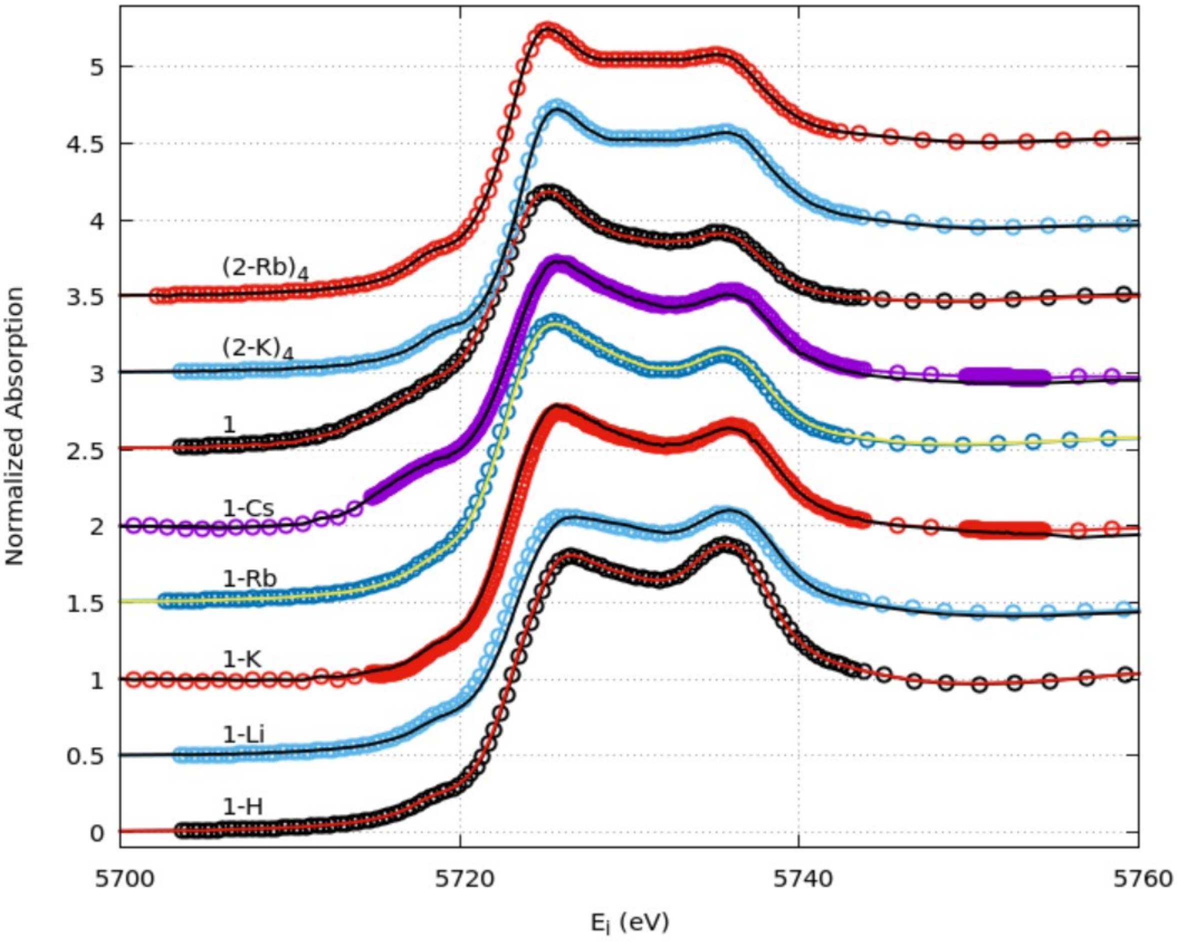


**Figure S13.** $L_{III}$ edge XANES spectra of TriNO$_x$ complexes at varying temperatures. None of the complexes exhibit temperature dependent spectral differences at 50 K (dotted lines) versus 300 K (solid lines in **1-H**, **1-Li**, **1-Rb**, **1**, **(2-K)$_4$**, and **(2-Rb)$_4$**) or 200 K (solid lines in **1-K** and **1-Cs**).

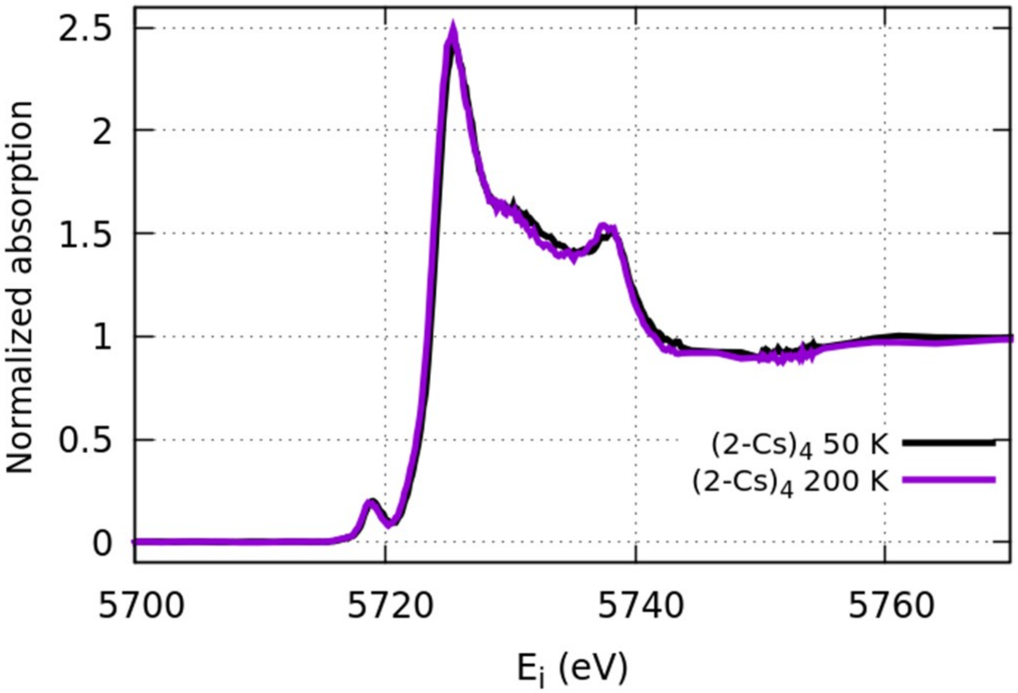


**Figure S14.** HERFD spectra of **(2-Cs)$_4$** show no temperature-dependent differences between 50 K and 200 K. HERFD data were shown instead of XANES, as temperature-dependent XANES data on this sample was not collected.

### *XANES fitting methods*

XANES data were fit in order to extract n($f^0$) according to previously described methods.[1-3] The fits consisted of a sum of a step-like function to model the absorption edge and two Gaussians to fit peaks associated with $f^{1,2}$ and $f^0$ configurations, in addition to a third Gaussian fit to a shoulder in the EXAFS region. The step-like function (integrated Gaussian) models excitations into the continuum whose position is given by a weighted average of the $f^{1,2}$ and $f^0$ peak energies, rather than using two step-like functions, in order to reduce the number of parameters in the fit and to control correlations between the fit parameters. This edge step is defined according to the expression:

$$I_{edge}(E) = \int_{-\infty}^{E} C_{edge} e^{\frac{-(E' - E_0)^2}{2\sigma^2}} dE',$$

and the Gaussians defined according to the expression:

$$I_i(E) = \frac{A_i}{\sqrt{2\pi}\sigma_i} e^{\frac{-(E - e_i)^2}{2\sigma_i^2}},$$

where E is the incident energy, $e_i$ is the peak energy, $\sigma_i$ is the half-width of the Gaussian and $I_i$ is the intensity of peak i. $E_0$ is constrained to be the average of the peak energies of $f^{1,2}$ and $f^0$ weighted by the area under each associated Gaussian, $A_i$. The Gaussian widths of the $f^{1,2}$ and $f^0$ peaks were held equal. The calculation of error bars for fitting parameters was achieved using a covariance matrix assuming normal distributions for variances in the data. Normally, the f-occupancy $n_f$ is calculated via:

$$n_f = \frac{A_{III}}{A_{III} + A_{IV}},$$

where $A_{III}$ and $A_{IV}$ are the areas for the so-called Ce(III) and Ce(IV) features in the spectra, as described below. However it is possible that any $f^2$ contribution will affect the area of the A(III) peak. This issue has been noted for Yb edges.[2] To avoid this issue, we instead report here the $f^0$ contribution:

$$n(f^0) = \frac{A_{IV}}{A_{III} + A_{IV}}.$$

Error bars on calculated $n(f^0)$ values determined from the $f^{1,2}$ and $f^0$ peak areas are estimated to be about 0.03 normalized units. Parameters reported without error bars were held fixed or constrained during the fit.

### *Results from XANES fitting used to determine n(f⁰)*

The following figures and tables show representative results from XANES fitting. In some cases, more than one data set per sample was collected, in which case the numbers in Table 1 of the manuscript represent average $n(f^0)$ values, where the error bars encompass any variation from data set to data set, which was small. Note that the fit quality, especially for the **(2-M)$_4$** samples, is not as high as has previously been reported in other formal Ce(IV) complexes.[3, 4] This is due to increased splitting of the 5d manifold. Despite this difference in the model, the 4f/5d feature was not used in determining $n(f^0)$, given the correlation with other parameters in the fitting procedure. A 3-peak fit was also attempted that uses a third peak fit to the 4f/5d feature and is shown in figure 10 and described below. We demonstrate, however, that the overall integrated intensity ratios between $f^{1,2}$ and $f^0$ are conserved regardless of using a 3 vs. 2-peak fitting model, and therefore the $n(f^0)$ results are deemed reliable to within the estimated errors. The pre-edge feature at ~5715 eV which is thought to arise from either 2p-4f quadrupole excitation[5] or from mixed d- and f-states[6] was not included in the fit due to its small contribution and high correlation with other parameters.

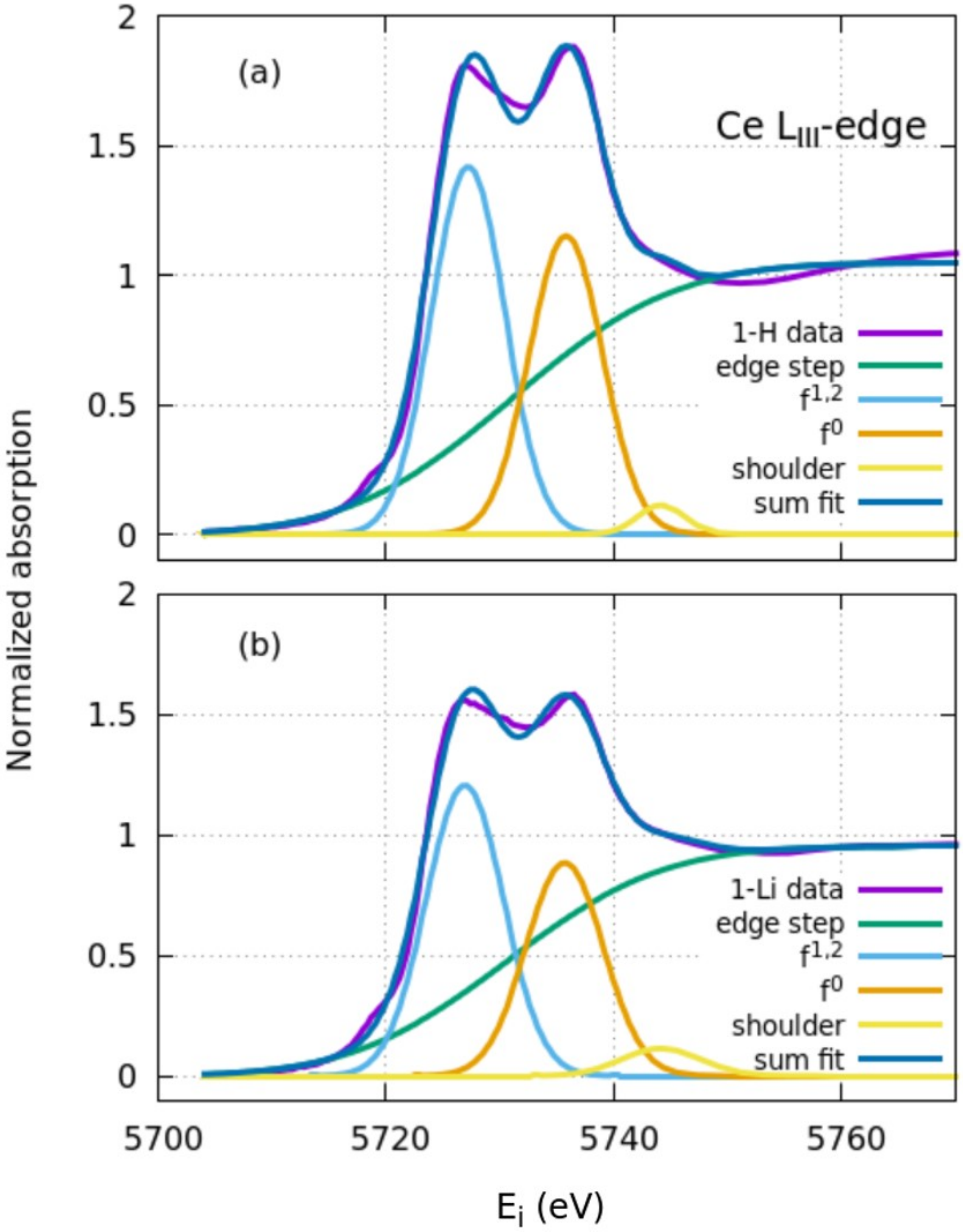


**Figure S15a.** Fit of XANES data for a) **1-H** and b) **1-Li** samples.

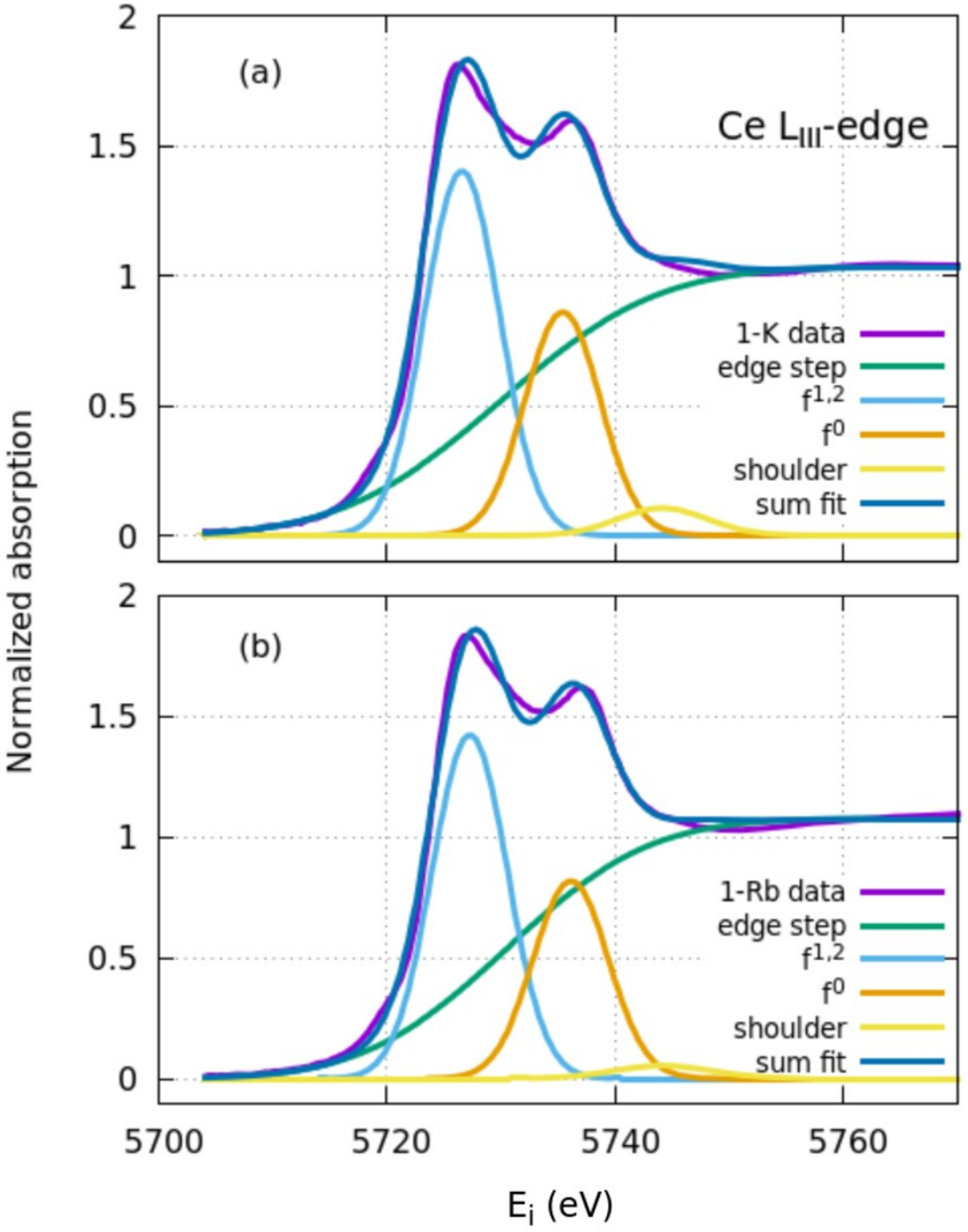


**Figure S15b.** Fit of XANES data for a) **1-K** and b) **1-Rb** samples.

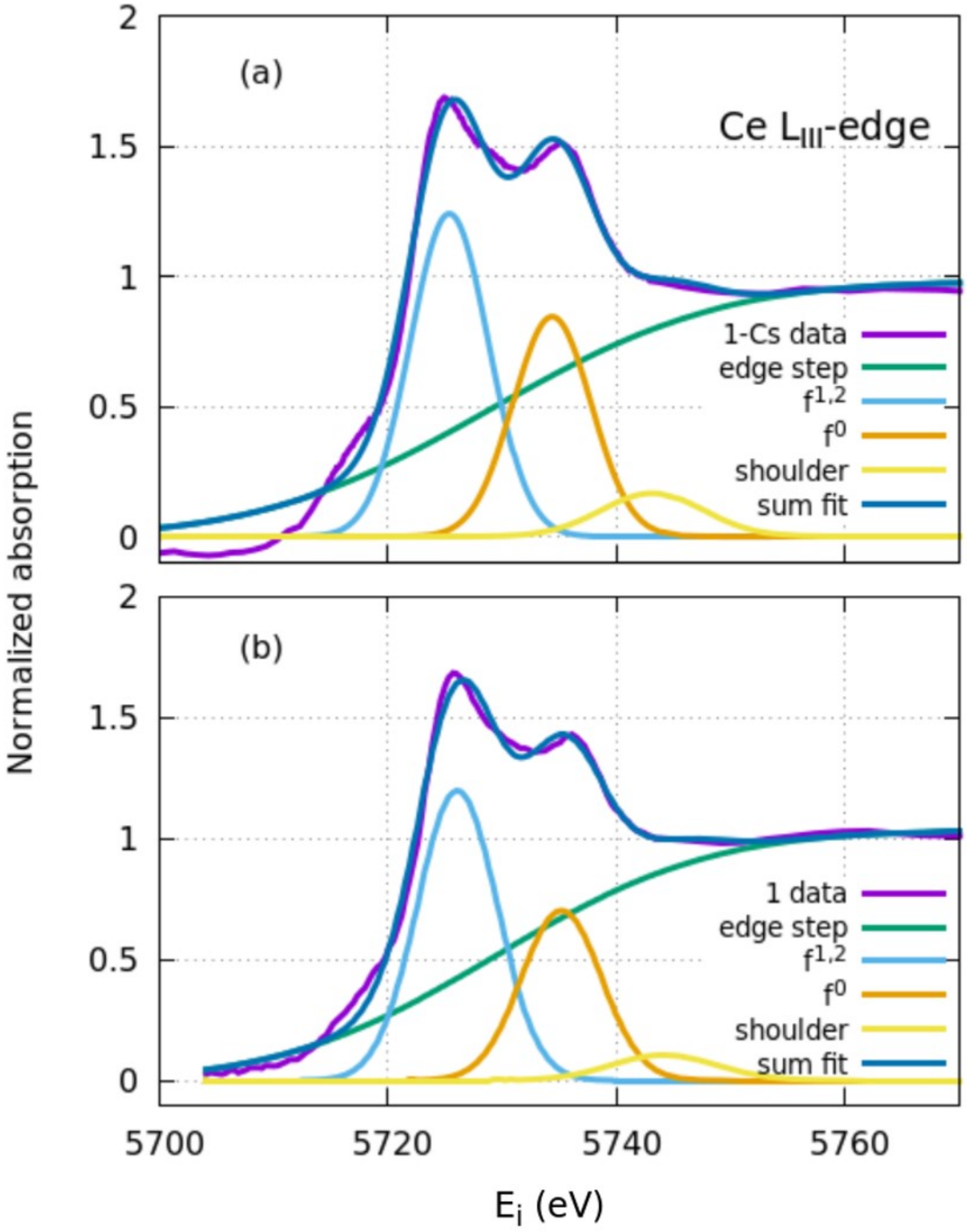


**Figure S15c.** Fit of XANES data for a) **1-Cs** and b) **1**$^-$ samples.

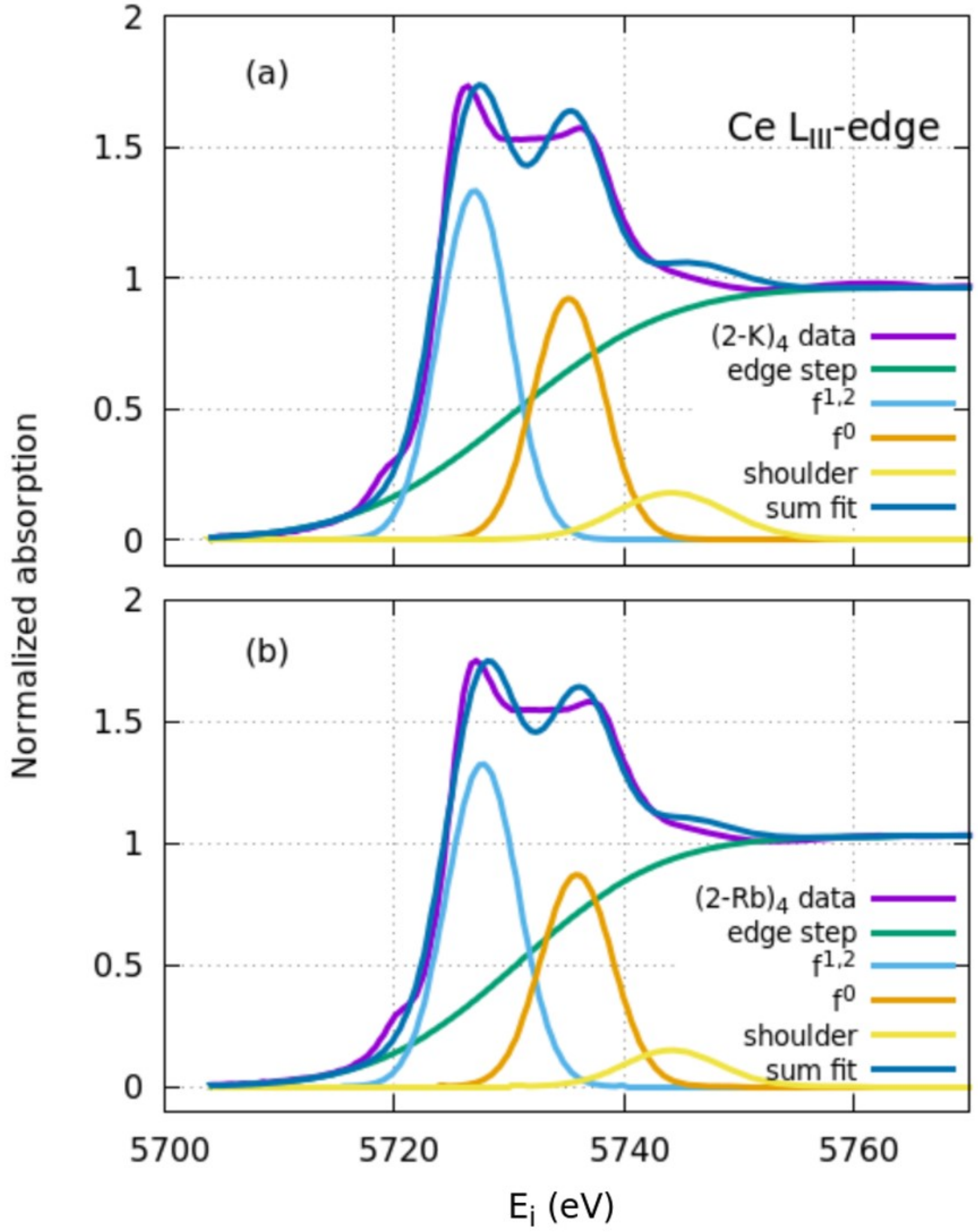


**Figure S15d.** Fit of XANES data for a) **(2-K)$_4$** and b) **(2-Rb)$_4$** samples.

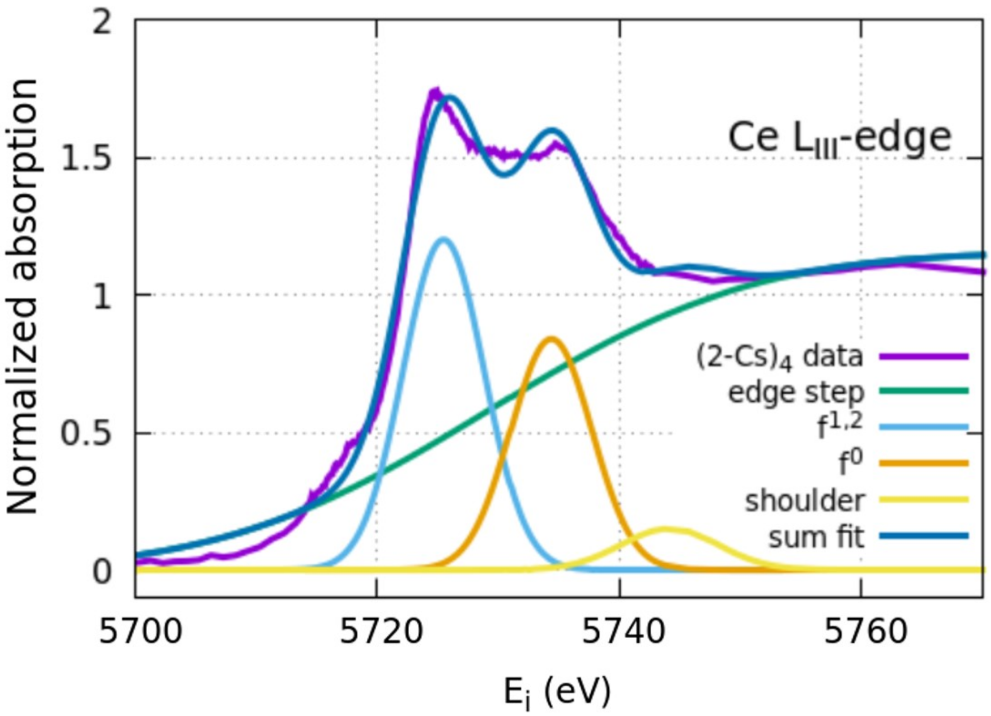


**Figure S15e.** Fit of XANES data for **(2-Cs)$_4$** sample.

**Table S1.** Fit parameter results for $f^{1,2}$ and $f^0$ integrated peak areas. Error estimates are determined from the covariance matrix and data errors determined by assuming the fitted $\chi^2$ parameter equals the degrees of freedom in the fit.

| Sample | $f^{1,2}$ peak area | $f^0$ peak area | R(%) from Fit |
|---|---|---|---|
| **1-H** | 11.4(2) | 9.3(2) | 2.6 |
| **1-Li** | 10.2(2) | 7.5(2) | 2.4 |
| **1-K** | 11.6(3) | 7.1(2) | 2.7 |
| **1-Rb** | 11.7(2) | 6.7(2) | 3.0 |
| **1-Cs** | 10.6(3) | 7.3(3) | 3.6 |
| **1$^-$** | 10.5(3) | 6.1(3) | 3.5 |
| **(2-K)$_4$** | 10.3(4) | 7.1(4) | 4.4 |
| **(2-Rb)$_4$** | 10.3(3) | 6.8(3) | 4.3 |
| **(2-Cs)$_4$** | 10.4(4) | 7.3(3) | 4.3 |

### *XANES fitting using a 3-peak vs. 2-peak model*

In order to estimate the error introduced into the n($f^0$) fits as a result of using a 2-peak model that cannot account for the "middle peak" which results from d-state broadening, a 3-peak simulation was used. Figure 10a shows a 3 peak simulation to the **(2-K)$_4$** XANES spectrum. As with the 2-peak fits, an EXAFS shoulder at higher energy is also included. The position of the middle peak was fixed to the weighted average of the energies of the $f^{1,2}$ and $f^0$ peaks. The simulation appears to match the experimental spectrum well, with an n($f^0$) value of 0.395. Through fitting the standard 2-peak model introduced earlier to this 3-peak simulation, the n($f^0$) value from the fit could be compared to a "known" n($f^0$) value from the simulation. The 2-peak model fit to the 3-peak simulation from Figure 10a is shown in Figure 10b. Interestingly, the n($f^0$) result from the 2-peak fit was nearly identical to that from the 3-peak simulation 0.4(3). Therefore, the error bar reported for the n($f^0$) values extracted from the 2-peak fits presented above should accurately encompass any error that would result from using a 2-peak vs. 3-peak model.

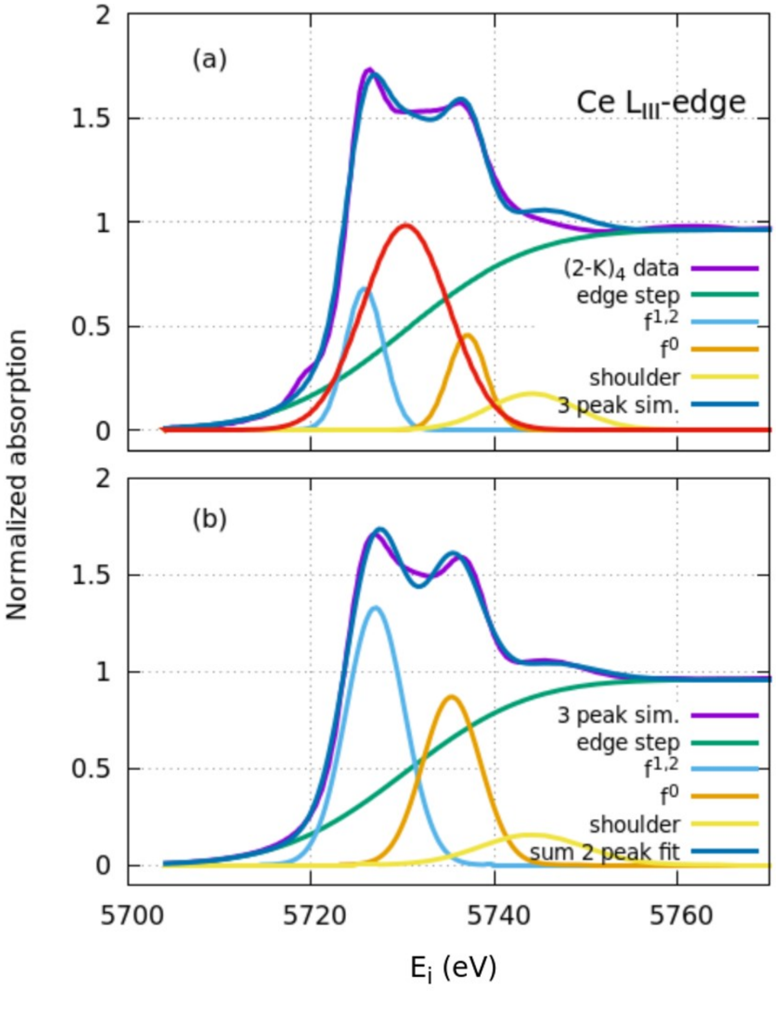


**Figure S16.** a) A simulation of **(2-K)$_4$** using 3 peaks ($f^{1,2}$, $f^0$ and an additional middle peak (red)) with a known n($f^0$) value of 0.395 matched well with the **(2-K)$_4$** experimental XANES data in comparison to the 2-peak fits to the **(2-M)$_4$** sample spectra. When the simulation was fit with a 2-

peak model (b) the resulting n($f^0$) value extracted was 0.4(3). Therefore, the 2-peak model was shown to still result in a representative value for n($f^0$) and legitimizes its use.

**Table S2. $f^{1,2}$ and $f^0$** peak energies from XANES data

| **Sample** | **$f^{1,2}$ peak energy (eV)** | **$f^0$ peak energy (eV)** |
|---|---|---|
| $CeCp^{tet}_3$ | 5724.6 | N/A |
| $CeO_2$ | 5729.4 | 5736.2 |
| **1-H** | 5726.5 | 5735.8 |
| **1-Li** | 5726.1 | 5735.7 |
| **1-K** | 5725.7 | 5735.7 |
| **1-Rb** | 5725.0 | 5735.2 |
| **1-Cs** | 5725.2 | 5736.0 |
| **1** | 5725.7 | 5735.4 |
| **$(2\text{-K})_4$** | 5725.7 | 5735.7 |
| **$(2\text{-Rb})_4$** | 5725.8 | 5735.9 |
| **$(2\text{-Cs})_4$** | 5725.9 | 5735.7 |

***Raw XANES and HERFD data***

**Table S3.** Anilide XANES

| **1-H** | |
|---|---|
| E(eV) | Normalized XANES |
| 5553.5 | 0.000481 |
| 5566.833 | 0.002966 |
| 5576.833 | 0.005604 |
| 5586.834 | 0.007039 |
| 5596.833 | 0.007 |
| 5606.833 | 0.006929 |
| 5616.833 | 0.006451 |
| 5626.834 | 0.006129 |
| 5636.834 | 0.004889 |
| 5646.835 | 0.003433 |
| 5656.834 | 0.000932 |
| 5666.833 | -0.001792 |
| 5676.833 | -0.001545 |
| 5686.833 | 0.00028 |
| 5696.834 | 0.003316 |
| 5703.617 | 0.009956 |

| | |
|---|---|
| 5703.964 | 0.011102 |
| 5704.311 | 0.010352 |
| 5704.659 | 0.010904 |
| 5705.007 | 0.012871 |
| 5705.355 | 0.013837 |
| 5705.704 | 0.014111 |
| 5706.052 | 0.015545 |
| 5706.399 | 0.01651 |
| 5706.746 | 0.018068 |
| 5707.094 | 0.018233 |
| 5707.443 | 0.019766 |
| 5707.791 | 0.020643 |
| 5708.137 | 0.021283 |
| 5708.486 | 0.022957 |
| 5708.834 | 0.025452 |
| 5709.181 | 0.026372 |
| 5709.529 | 0.027495 |
| 5709.877 | 0.029257 |
| 5710.225 | 0.031167 |
| 5710.572 | 0.032342 |
| 5710.919 | 0.035095 |
| 5711.268 | 0.037873 |
| 5711.615 | 0.040291 |
| 5711.963 | 0.042984 |
| 5712.311 | 0.046231 |
| 5712.659 | 0.050087 |
| 5713.007 | 0.053348 |
| 5713.355 | 0.058176 |
| 5713.703 | 0.062971 |
| 5714.052 | 0.067511 |
| 5714.398 | 0.073225 |
| 5714.746 | 0.07902 |
| 5715.095 | 0.087418 |
| 5715.441 | 0.09506 |
| 5715.79 | 0.10492 |
| 5716.138 | 0.11722 |
| 5716.485 | 0.131803 |
| 5716.833 | 0.147728 |
| 5717.181 | 0.168203 |
| 5717.53 | 0.188218 |
| 5717.877 | 0.210187 |
| 5718.224 | 0.228434 |
| 5718.571 | 0.242698 |

| | |
|---|---|
| 5718.92 | 0.256569 |
| 5719.269 | 0.270628 |
| 5719.617 | 0.28775 |
| 5719.964 | 0.31018 |
| 5720.312 | 0.340786 |
| 5720.659 | 0.380806 |
| 5721.007 | 0.431403 |
| 5721.355 | 0.495774 |
| 5721.703 | 0.574725 |
| 5722.051 | 0.668475 |
| 5722.398 | 0.776662 |
| 5722.747 | 0.897776 |
| 5723.095 | 1.022846 |
| 5723.443 | 1.145683 |
| 5723.791 | 1.262972 |
| 5724.139 | 1.372017 |
| 5724.486 | 1.475514 |
| 5724.833 | 1.571992 |
| 5725.181 | 1.65861 |
| 5725.529 | 1.727437 |
| 5725.877 | 1.773039 |
| 5726.225 | 1.798312 |
| 5726.572 | 1.804183 |
| 5726.92 | 1.796729 |
| 5727.269 | 1.781707 |
| 5727.617 | 1.765805 |
| 5727.963 | 1.750555 |
| 5728.312 | 1.734741 |
| 5728.66 | 1.720109 |
| 5729.007 | 1.708143 |
| 5729.355 | 1.69787 |
| 5729.702 | 1.687599 |
| 5730.051 | 1.67686 |
| 5730.399 | 1.666961 |
| 5730.746 | 1.65865 |
| 5731.095 | 1.651292 |
| 5731.442 | 1.647412 |
| 5731.791 | 1.646258 |
| 5732.138 | 1.650859 |
| 5732.486 | 1.660676 |
| 5732.833 | 1.676766 |
| 5733.181 | 1.700457 |
| 5733.529 | 1.728757 |

| | |
|---|---|
| 5733.877 | 1.760414 |
| 5734.226 | 1.794063 |
| 5734.572 | 1.826808 |
| 5734.92 | 1.852781 |
| 5735.269 | 1.869534 |
| 5735.616 | 1.877196 |
| 5735.964 | 1.875827 |
| 5736.313 | 1.861838 |
| 5736.66 | 1.832887 |
| 5737.007 | 1.789297 |
| 5737.354 | 1.730023 |
| 5737.703 | 1.659579 |
| 5738.052 | 1.58366 |
| 5738.399 | 1.50933 |
| 5738.747 | 1.437427 |
| 5739.094 | 1.37487 |
| 5739.441 | 1.318939 |
| 5739.789 | 1.272277 |
| 5740.137 | 1.232819 |
| 5740.484 | 1.199632 |
| 5740.832 | 1.172625 |
| 5741.18 | 1.150441 |
| 5741.528 | 1.131442 |
| 5741.875 | 1.115151 |
| 5742.224 | 1.101622 |
| 5742.572 | 1.088529 |
| 5742.92 | 1.077195 |
| 5743.268 | 1.066392 |
| 5743.787 | 1.05265 |
| 5744.958 | 1.023991 |
| 5746.771 | 0.989744 |
| 5748.658 | 0.971235 |
| 5750.62 | 0.966871 |
| 5752.655 | 0.972926 |
| 5754.764 | 0.987667 |
| 5756.945 | 1.00882 |
| 5759.2 | 1.029886 |
| 5761.528 | 1.045707 |
| 5763.931 | 1.060137 |
| 5766.406 | 1.072855 |
| 5768.957 | 1.081318 |
| 5771.581 | 1.086399 |
| 5774.277 | 1.084931 |

| | |
|---|---|
| 5777.047 | 1.077324 |
| 5779.892 | 1.065379 |
| 5782.808 | 1.052437 |
| 5785.798 | 1.039742 |
| 5788.863 | 1.027594 |
| 5792.002 | 1.015756 |
| 5795.214 | 1.007173 |
| 5798.5 | 1.002691 |
| 5801.86 | 1.000211 |
| 5805.293 | 0.999612 |
| 5808.798 | 1.000282 |
| 5812.377 | 1.004505 |
| 5816.032 | 1.00999 |
| 5819.758 | 1.013614 |
| 5823.56 | 1.017493 |
| 5827.434 | 1.026105 |
| 5831.382 | 1.030488 |
| 5835.404 | 1.030568 |
| 5839.499 | 1.029649 |
| 5843.667 | 1.029951 |
| 5847.909 | 1.034099 |
| 5852.226 | 1.037184 |
| 5856.614 | 1.035149 |
| 5861.078 | 1.024421 |
| 5865.615 | 1.011638 |

**Table S4.** Imido XANES (**1-Li**, **1-K**, and **1-Rb**)

| **1-Li** | | **1-K** | | **1-Rb** | |
|---|---|---|---|---|---|
| E(eV) | Normalized XANES | E(eV) | Normalized XANES | E(eV) | Normalized XANES |
| 5553.40049 | -5.34E-02 | 5553.4005 | 1.59E-02 | 5552.5005 | -0.06784484 |
| 5566.73252 | -4.96E-02 | 5566.7325 | 1.11E-02 | 5565.8325 | -0.04607055 |
| 5576.73301 | -4.91E-02 | 5576.733 | 7.61E-03 | 5575.833 | -0.02800729 |
| 5586.7335 | -3.81E-02 | 5586.7335 | 5.14E-03 | 5585.8335 | -0.01482073 |
| 5596.73252 | -3.03E-02 | 5596.7325 | 3.95E-03 | 5595.8325 | -0.00751829 |
| 5606.73252 | -2.67E-02 | 5606.7325 | -3.64E-05 | 5605.8325 | -0.00329756 |
| 5616.73301 | -2.27E-02 | 5616.733 | -5.54E-03 | 5615.833 | 0.001737218 |
| 5626.73398 | -8.08E-03 | 5626.734 | 1.79E-03 | 5625.834 | 0.001650672 |
| 5636.73398 | 1.17E-03 | 5636.734 | 2.48E-03 | 5635.834 | 0.003519306 |
| 5646.73496 | 2.94E-03 | 5646.735 | 2.93E-03 | 5645.835 | 0.001773595 |
| 5656.73447 | -6.36E-04 | 5656.7345 | -8.60E-04 | 5655.8345 | 0.001068324 |

| | | | | | |
|---|---|---|---|---|---|
| 5666.73301 | 8.79E-04 | 5666.733 | -7.49E-04 | 5665.833 | 0.000144824 |
| 5676.73301 | 5.27E-05 | 5676.733 | -1.95E-03 | 5675.833 | -0.00258339 |
| 5686.73301 | -1.21E-03 | 5686.733 | -1.07E-03 | 5685.833 | -0.00210925 |
| 5696.73398 | 9.62E-04 | 5696.734 | 4.17E-03 | 5695.834 | 0.002397203 |
| 5703.51719 | 8.01E-04 | 5703.5172 | 9.74E-03 | 5702.6172 | 0.009599048 |
| 5703.86436 | 2.42E-03 | 5703.8644 | 8.67E-03 | 5702.9644 | 0.010533163 |
| 5704.21055 | 4.97E-03 | 5704.2106 | 9.66E-03 | 5703.3106 | 0.010493236 |
| 5704.55869 | 4.59E-03 | 5704.5587 | 1.27E-02 | 5703.6587 | 0.010897409 |
| 5704.90684 | 7.61E-04 | 5704.9068 | 1.29E-02 | 5704.0068 | 0.01233446 |
| 5705.25498 | 3.82E-03 | 5705.255 | 1.24E-02 | 5704.355 | 0.012625537 |
| 5705.60361 | 6.69E-03 | 5705.6036 | 1.26E-02 | 5704.7036 | 0.013335417 |
| 5705.95176 | 6.67E-03 | 5705.9518 | 1.10E-02 | 5705.0518 | 0.014874729 |
| 5706.29941 | 6.47E-03 | 5706.2994 | 1.16E-02 | 5705.3994 | 0.016908407 |
| 5706.64561 | 7.65E-03 | 5706.6456 | 1.46E-02 | 5705.7456 | 0.015821073 |
| 5706.99424 | 1.16E-02 | 5706.9942 | 2.04E-02 | 5706.0942 | 0.015282807 |
| 5707.34287 | 1.28E-02 | 5707.3429 | 1.87E-02 | 5706.4429 | 0.015645305 |
| 5707.69053 | 1.13E-02 | 5707.6905 | 1.79E-02 | 5706.7905 | 0.017109644 |
| 5708.03721 | 1.32E-02 | 5708.0372 | 2.13E-02 | 5707.1372 | 0.018950167 |
| 5708.38584 | 1.46E-02 | 5708.3858 | 2.21E-02 | 5707.4858 | 0.021117362 |
| 5708.7335 | 1.67E-02 | 5708.7335 | 2.55E-02 | 5707.8335 | 0.024025889 |
| 5709.08066 | 1.81E-02 | 5709.0807 | 2.86E-02 | 5708.1807 | 0.023742725 |
| 5709.4293 | 2.03E-02 | 5709.4293 | 3.02E-02 | 5708.5293 | 0.025471943 |
| 5709.77744 | 2.11E-02 | 5709.7774 | 3.11E-02 | 5708.8774 | 0.027865789 |
| 5710.12461 | 2.24E-02 | 5710.1246 | 3.32E-02 | 5709.2246 | 0.030027706 |
| 5710.47227 | 2.46E-02 | 5710.4723 | 3.48E-02 | 5709.5723 | 0.031827123 |
| 5710.81943 | 2.66E-02 | 5710.8194 | 3.70E-02 | 5709.9194 | 0.033903461 |
| 5711.16807 | 2.87E-02 | 5711.1681 | 4.19E-02 | 5710.2681 | 0.035666014 |
| 5711.51523 | 3.29E-02 | 5711.5152 | 4.55E-02 | 5710.6152 | 0.038179196 |
| 5711.86338 | 3.74E-02 | 5711.8634 | 4.56E-02 | 5710.9634 | 0.040484586 |
| 5712.21104 | 3.83E-02 | 5712.211 | 5.20E-02 | 5711.311 | 0.043209022 |
| 5712.55918 | 4.16E-02 | 5712.5592 | 5.75E-02 | 5711.6592 | 0.046016885 |
| 5712.90684 | 4.58E-02 | 5712.9068 | 6.19E-02 | 5712.0068 | 0.049411983 |
| 5713.25547 | 4.90E-02 | 5713.2555 | 6.39E-02 | 5712.3555 | 0.053570552 |
| 5713.60312 | 5.71E-02 | 5713.6031 | 6.90E-02 | 5712.7031 | 0.05814318 |
| 5713.95176 | 6.50E-02 | 5713.9518 | 7.43E-02 | 5713.0518 | 0.062734261 |
| 5714.29844 | 7.02E-02 | 5714.2984 | 8.18E-02 | 5713.3984 | 0.067103288 |
| 5714.64609 | 7.62E-02 | 5714.6461 | 9.25E-02 | 5713.7461 | 0.073176169 |
| 5714.99473 | 8.49E-02 | 5714.9947 | 1.01E-01 | 5714.0947 | 0.079197078 |
| 5715.34141 | 9.45E-02 | 5715.3414 | 1.09E-01 | 5714.4414 | 0.085935403 |
| 5715.68955 | 0.1067537 | 5715.6896 | 1.22E-01 | 5714.7896 | 0.095101873 |
| 5716.03818 | 0.1190924 | 5716.0382 | 1.35E-01 | 5715.1382 | 0.104395747 |
| 5716.38535 | 0.1341301 | 5716.3854 | 1.52E-01 | 5715.4854 | 0.116598877 |
| 5716.73252 | 0.1541431 | 5716.7325 | 1.71E-01 | 5715.8325 | 0.128940019 |

| | | | | | |
|---|---|---|---|---|---|
| 5717.08066 | 0.1793683 | 5717.0807 | 1.94E-01 | 5716.1807 | 0.142930258 |
| 5717.42979 | 0.2005443 | 5717.4298 | 2.18E-01 | 5716.5298 | 0.160206827 |
| 5717.77695 | 0.222842 | 5717.777 | 2.41E-01 | 5716.877 | 0.17958385 |
| 5718.12412 | 0.2508308 | 5718.1241 | 2.66E-01 | 5717.2241 | 0.200603618 |
| 5718.47129 | 0.2723683 | 5718.4713 | 2.93E-01 | 5717.5713 | 0.223119435 |
| 5718.81992 | 0.2905191 | 5718.8199 | 3.17E-01 | 5717.9199 | 0.247587751 |
| 5719.16904 | 0.309051 | 5719.169 | 3.38E-01 | 5718.269 | 0.27091598 |
| 5719.5167 | 0.3272207 | 5719.5167 | 3.68E-01 | 5718.6167 | 0.292495028 |
| 5719.86436 | 0.3501892 | 5719.8644 | 4.03E-01 | 5718.9644 | 0.314692557 |
| 5720.21152 | 0.3811402 | 5720.2115 | 4.42E-01 | 5719.3115 | 0.340917734 |
| 5720.55918 | 0.4262558 | 5720.5592 | 4.96E-01 | 5719.6592 | 0.3748292 |
| 5720.90732 | 0.4785726 | 5720.9073 | 5.66E-01 | 5720.0073 | 0.412588905 |
| 5721.25498 | 0.5421402 | 5721.255 | 6.48E-01 | 5720.355 | 0.461579507 |
| 5721.60264 | 0.6244491 | 5721.6026 | 7.45E-01 | 5720.7026 | 0.524590557 |
| 5721.95078 | 0.7164172 | 5721.9508 | 8.50E-01 | 5721.0508 | 0.599230853 |
| 5722.29844 | 0.8175204 | 5722.2984 | 9.61E-01 | 5721.3984 | 0.685766447 |
| 5722.64658 | 0.9337112 | 5722.6466 | 1.08E+00 | 5721.7466 | 0.787800875 |
| 5722.99473 | 1.051497 | 5722.9947 | 1.21E+00 | 5722.0947 | 0.895812457 |
| 5723.34287 | 1.1641839 | 5723.3429 | 1.34E+00 | 5722.4429 | 1.011448018 |
| 5723.69102 | 1.2698321 | 5723.691 | 1.46E+00 | 5722.791 | 1.135332264 |
| 5724.03916 | 1.365004 | 5724.0392 | 1.56E+00 | 5723.1392 | 1.2576722 |
| 5724.38633 | 1.4434252 | 5724.3863 | 1.65E+00 | 5723.4863 | 1.367462655 |
| 5724.73252 | 1.5092675 | 5724.7325 | 1.71E+00 | 5723.8325 | 1.465307255 |
| 5725.08066 | 1.5608332 | 5725.0807 | 1.75E+00 | 5724.1807 | 1.544490291 |
| 5725.4293 | 1.598173 | 5725.4293 | 1.77E+00 | 5724.5293 | 1.603626227 |
| 5725.77744 | 1.6201855 | 5725.7774 | 1.78E+00 | 5724.8774 | 1.641510945 |
| 5726.1251 | 1.6271222 | 5726.1251 | 1.76E+00 | 5725.2251 | 1.661825936 |
| 5726.47178 | 1.6252412 | 5726.4718 | 1.74E+00 | 5725.5718 | 1.667103445 |
| 5726.82041 | 1.6182307 | 5726.8204 | 1.72E+00 | 5725.9204 | 1.658356409 |
| 5727.16904 | 1.6136583 | 5727.169 | 1.69E+00 | 5726.269 | 1.639585491 |
| 5727.5167 | 1.6043952 | 5727.5167 | 1.66E+00 | 5726.6167 | 1.6173418 |
| 5727.86338 | 1.5941909 | 5727.8634 | 1.64E+00 | 5726.9634 | 1.5927745 |
| 5728.21201 | 1.5868623 | 5728.212 | 1.61E+00 | 5727.312 | 1.568079864 |
| 5728.56016 | 1.5750226 | 5728.5602 | 1.59E+00 | 5727.6602 | 1.546464509 |
| 5728.90684 | 1.5678893 | 5728.9068 | 1.58E+00 | 5728.0068 | 1.526462927 |
| 5729.25498 | 1.5567477 | 5729.255 | 1.56E+00 | 5728.355 | 1.506218473 |
| 5729.60215 | 1.552158 | 5729.6022 | 1.54E+00 | 5728.7022 | 1.488730145 |
| 5729.95078 | 1.5461416 | 5729.9508 | 1.53E+00 | 5729.0508 | 1.472476291 |
| 5730.29893 | 1.5353957 | 5730.2989 | 1.52E+00 | 5729.3989 | 1.454215155 |
| 5730.64609 | 1.5261346 | 5730.6461 | 1.51E+00 | 5729.7461 | 1.437455955 |
| 5730.99473 | 1.5196094 | 5730.9947 | 1.50E+00 | 5730.0947 | 1.423360109 |
| 5731.34189 | 1.5149781 | 5731.3419 | 1.49E+00 | 5730.4419 | 1.407603355 |
| 5731.69053 | 1.5104522 | 5731.6905 | 1.48E+00 | 5730.7905 | 1.393866536 |

| | | | | | |
|---|---|---|---|---|---|
| 5732.0377 | 1.5128805 | 5732.0377 | 1.48E+00 | 5731.1377 | 1.384461573 |
| 5732.38633 | 1.5139685 | 5732.3863 | 1.48E+00 | 5731.4863 | 1.382561273 |
| 5732.73301 | 1.5202464 | 5732.733 | 1.48E+00 | 5731.833 | 1.3784608 |
| 5733.08066 | 1.5318785 | 5733.0807 | 1.49E+00 | 5732.1807 | 1.378757409 |
| 5733.4293 | 1.5480691 | 5733.4293 | 1.50E+00 | 5732.5293 | 1.379987218 |
| 5733.77695 | 1.5658782 | 5733.777 | 1.51E+00 | 5732.877 | 1.383969027 |
| 5734.12559 | 1.5846565 | 5734.1256 | 1.52E+00 | 5733.2256 | 1.395395018 |
| 5734.47227 | 1.6052861 | 5734.4723 | 1.54E+00 | 5733.5723 | 1.406279582 |
| 5734.81992 | 1.6273016 | 5734.8199 | 1.55E+00 | 5733.9199 | 1.4198365 |
| 5735.16904 | 1.6426315 | 5735.169 | 1.56E+00 | 5734.269 | 1.432140527 |
| 5735.51572 | 1.6499138 | 5735.5157 | 1.57E+00 | 5734.6157 | 1.449408527 |
| 5735.86387 | 1.6498148 | 5735.8639 | 1.57E+00 | 5734.9639 | 1.463851064 |
| 5736.21299 | 1.6371192 | 5736.213 | 1.55E+00 | 5735.313 | 1.471506573 |
| 5736.55967 | 1.6183476 | 5736.5597 | 1.53E+00 | 5735.6597 | 1.470626809 |
| 5736.90684 | 1.5888605 | 5736.9068 | 1.51E+00 | 5736.0068 | 1.465484009 |
| 5737.254 | 1.5469518 | 5737.254 | 1.47E+00 | 5736.354 | 1.451225609 |
| 5737.60264 | 1.5053079 | 5737.6026 | 1.43E+00 | 5736.7026 | 1.424205518 |
| 5737.95176 | 1.4589891 | 5737.9518 | 1.39E+00 | 5737.0518 | 1.393372364 |
| 5738.29893 | 1.4077003 | 5738.2989 | 1.34E+00 | 5737.3989 | 1.355901955 |
| 5738.64658 | 1.3598463 | 5738.6466 | 1.30E+00 | 5737.7466 | 1.313872336 |
| 5738.99375 | 1.315705 | 5738.9938 | 1.26E+00 | 5738.0938 | 1.271022018 |
| 5739.34141 | 1.2739024 | 5739.3414 | 1.22E+00 | 5738.4414 | 1.226511109 |
| 5739.68906 | 1.2354255 | 5739.6891 | 1.19E+00 | 5738.7891 | 1.187335255 |
| 5740.03672 | 1.2026656 | 5740.0367 | 1.16E+00 | 5739.1367 | 1.151579509 |
| 5740.38438 | 1.1763214 | 5740.3844 | 1.13E+00 | 5739.4844 | 1.120019718 |
| 5740.73203 | 1.153834 | 5740.732 | 1.12E+00 | 5739.832 | 1.092563973 |
| 5741.07969 | 1.1333939 | 5741.0797 | 1.10E+00 | 5740.1797 | 1.070961627 |
| 5741.42783 | 1.1144794 | 5741.4278 | 1.08E+00 | 5740.5278 | 1.050553209 |
| 5741.77549 | 1.1004218 | 5741.7755 | 1.07E+00 | 5740.8755 | 1.034964755 |
| 5742.12363 | 1.0875467 | 5742.1236 | 1.06E+00 | 5741.2236 | 1.021826918 |
| 5742.47178 | 1.0769635 | 5742.4718 | 1.05E+00 | 5741.5718 | 1.0117908 |
| 5742.81992 | 1.0693459 | 5742.8199 | 1.04E+00 | 5741.9199 | 1.001076809 |
| 5743.16807 | 1.0614085 | 5743.1681 | 1.03E+00 | 5742.2681 | 0.991689618 |
| 5743.68711 | 1.0493558 | 5743.6871 | 1.02E+00 | 5742.7871 | 0.984460982 |
| 5744.85801 | 1.0275859 | 5744.858 | 1.01E+00 | 5743.958 | 0.966827109 |
| 5746.67148 | 1.0021559 | 5746.6715 | 9.90E-01 | 5745.7715 | 0.946072555 |
| 5748.5582 | 0.9832374 | 5748.5582 | 9.78E-01 | 5747.6582 | 0.936159764 |
| 5750.51963 | 0.9747641 | 5750.5196 | 9.80E-01 | 5749.6196 | 0.935912027 |
| 5752.55479 | 0.9675172 | 5752.5548 | 9.84E-01 | 5751.6548 | 0.940623282 |
| 5754.66367 | 0.9689783 | 5754.6637 | 9.93E-01 | 5753.7637 | 0.950101936 |
| 5756.84482 | 0.9793085 | 5756.8448 | 1.00E+00 | 5755.9448 | 0.961079164 |
| 5759.1002 | 0.9892849 | 5759.1002 | 1.01E+00 | 5758.2002 | 0.970588273 |
| 5761.42783 | 1.0003282 | 5761.4278 | 1.01E+00 | 5760.5278 | 0.976875909 |

| | | | | | |
|---|---|---|---|---|---|
| 5763.83066 | 1.0019296 | 5763.8307 | 1.02E+00 | 5762.9307 | 0.983634755 |
| 5766.30625 | 1.0037667 | 5766.3063 | 1.02E+00 | 5765.4063 | 0.988986709 |
| 5768.85703 | 1.0069669 | 5768.857 | 1.02E+00 | 5767.957 | 0.993425627 |
| 5771.48057 | 1.0053344 | 5771.4806 | 1.02E+00 | 5770.5806 | 0.998554555 |
| 5774.17734 | 1.0034969 | 5774.1773 | 1.01E+00 | 5773.2773 | 0.9970148 |
| 5776.94736 | 0.9966806 | 5776.9474 | 1.00E+00 | 5776.0474 | 0.993841236 |
| 5779.7916 | 0.9784971 | 5779.7916 | 9.96E-01 | 5778.8916 | 0.989307382 |
| 5782.70811 | 0.9630728 | 5782.7081 | 9.89E-01 | 5781.8081 | 0.981153245 |
| 5785.69834 | 0.9482784 | 5785.6983 | 9.82E-01 | 5784.7983 | 0.9778893 |
| 5788.76328 | 0.9354794 | 5788.7633 | 9.74E-01 | 5787.8633 | 0.976828118 |
| 5791.90195 | 0.924174 | 5791.902 | 9.64E-01 | 5791.002 | 0.975768673 |
| 5795.11387 | 0.9113269 | 5795.1139 | 9.55E-01 | 5794.2139 | 0.975712318 |
| 5798.4 | 0.8999259 | 5798.4 | 9.49E-01 | 5797.5 | 0.974295464 |
| 5801.75986 | 0.8890821 | 5801.7599 | 9.45E-01 | 5800.8599 | 0.974736755 |
| 5805.19297 | 0.8789557 | 5805.193 | 9.41E-01 | 5804.293 | 0.973053091 |
| 5808.69834 | 0.8691413 | 5808.6983 | 9.38E-01 | 5807.7983 | 0.973536209 |
| 5812.27744 | 0.8571719 | 5812.2774 | 9.35E-01 | 5811.3774 | 0.975850491 |
| 5815.93223 | 0.8479618 | 5815.9322 | 9.29E-01 | 5815.0322 | 0.979537855 |
| 5819.6583 | 0.8338477 | 5819.6583 | 9.27E-01 | 5818.7583 | 0.981404564 |
| 5823.45957 | 0.8272767 | 5823.4596 | 9.23E-01 | 5822.5596 | 0.966197036 |
| 5827.33408 | 0.8373204 | 5827.3341 | 9.29E-01 | 5826.4341 | 0.959903109 |
| 5831.28232 | 0.8313869 | 5831.2823 | 9.30E-01 | 5830.3823 | 1.018108345 |
| 5835.30381 | 0.8176108 | 5835.3038 | 9.24E-01 | 5834.4038 | 1.019385618 |
| 5839.39854 | 0.8082248 | 5839.3985 | 9.18E-01 | 5838.4985 | 1.021111445 |
| 5843.56748 | 0.7989965 | 5843.5675 | 9.14E-01 | 5842.6675 | 1.024416682 |
| 5847.80869 | 0.7905131 | 5847.8087 | 9.17E-01 | 5846.9087 | 1.029645855 |
| 5852.12607 | 0.7793599 | 5852.1261 | 9.15E-01 | 5851.2261 | 1.033032482 |
| 5856.51426 | 0.7663467 | 5856.5143 | 9.05E-01 | 5855.6143 | 1.0351427 |
| 5860.97812 | 0.7441254 | 5860.9781 | 8.91E-01 | 5860.0781 | 1.033273173 |
| 5865.51523 | 0.7195885 | 5865.5152 | 8.74E-01 | 5864.6152 | 1.028076845 |

**Table S5.** Imido XANES (**1-Cs** and **1**$^-$)

| **1-Cs** | | **1**$^-$ | |
|---|---|---|---|
| E(eV) | Normalized XANES | E(eV) | Normalized XANES |
| 5580.8 | 0.309392482 | 5553.40049 | -7.77E-02 |
| 5590.79951 | 0.313898236 | 5566.73252 | -6.68E-02 |
| 5600.80049 | 0.304259926 | 5576.73301 | -5.20E-02 |
| 5610.79951 | 0.259761661 | 5586.7335 | -3.86E-02 |
| 5620.8 | 0.233820677 | 5596.73252 | -3.33E-02 |
| 5630.80049 | 0.212604538 | 5606.73252 | -2.44E-02 |
| 5640.8 | 0.222777367 | 5616.73301 | -1.88E-02 |

| | | | |
|---|---|---|---|
| 5650.8 | 0.216378227 | 5626.73398 | -9.50E-03 |
| 5660.8 | 0.219338581 | 5636.73398 | 2.45E-04 |
| 5670.8 | 0.171896383 | 5646.73496 | 4.45E-03 |
| 5680.80049 | 0.14043206 | 5656.73447 | 1.56E-03 |
| 5681.80049 | 0.129954383 | 5666.73301 | -1.03E-03 |
| 5682.8 | 0.124737829 | 5676.73301 | -3.81E-03 |
| 5683.79951 | 0.118943684 | 5686.73301 | -5.53E-04 |
| 5684.8 | 0.101963937 | 5696.73398 | 5.30E-03 |
| 5685.8 | 0.094915144 | 5703.51719 | 1.41E-02 |
| 5686.8 | 0.090352796 | 5703.86436 | 2.18E-02 |
| 5687.79951 | 0.084419012 | 5704.21055 | 1.78E-02 |
| 5688.8 | 0.070879377 | 5704.55869 | 1.76E-02 |
| 5689.80049 | 0.076527089 | 5704.90684 | 1.70E-02 |
| 5690.80049 | 0.057188522 | 5705.25498 | 2.38E-02 |
| 5691.79951 | 0.049757455 | 5705.60361 | 2.63E-02 |
| 5692.79951 | 0.045594912 | 5705.95176 | 2.58E-02 |
| 5693.8 | 0.03387424 | 5706.29941 | 2.56E-02 |
| 5694.79951 | 0.027203396 | 5706.64561 | 2.70E-02 |
| 5695.8 | 0.030833313 | 5706.99424 | 2.82E-02 |
| 5696.80049 | 0.015712734 | 5707.34287 | 3.74E-02 |
| 5697.79951 | 0.018769098 | 5707.69053 | 4.29E-02 |
| 5698.8 | 0.00917671 | 5708.03721 | 4.16E-02 |
| 5699.8 | 0.00659235 | 5708.38584 | 4.31E-02 |
| 5700.80049 | -0.00207253 | 5708.7335 | 4.82E-02 |
| 5701.8 | -0.00156069 | 5709.08066 | 4.96E-02 |
| 5702.80049 | -0.00930035 | 5709.4293 | 5.20E-02 |
| 5703.8 | -0.01590575 | 5709.77744 | 5.59E-02 |
| 5704.80049 | -0.01695128 | 5710.12461 | 6.18E-02 |
| 5705.8 | -0.02280265 | 5710.47227 | 6.83E-02 |
| 5706.8 | -0.01326754 | 5710.81943 | 7.44E-02 |
| 5707.80049 | -0.01181218 | 5711.16807 | 7.88E-02 |
| 5708.8 | -0.01186572 | 5711.51523 | 8.27E-02 |
| 5709.80049 | 0.00560458 | 5711.86338 | 9.53E-02 |
| 5710.8 | 0.009620191 | 5712.21104 | 0.1102704 |
| 5711.80049 | 0.048906311 | 5712.55918 | 0.1192734 |
| 5712.8 | 0.056853738 | 5712.90684 | 0.1290536 |
| 5713.8 | 0.106925525 | 5713.25547 | 0.1492835 |
| 5714.80049 | 0.183904514 | 5713.60312 | 0.167489 |
| 5714.89961 | 0.187141359 | 5713.95176 | 0.1863802 |
| 5715.0002 | 0.193100095 | 5714.29844 | 0.2028608 |
| 5715.10029 | 0.198375776 | 5714.64609 | 0.2187945 |
| 5715.19941 | 0.205624402 | 5714.99473 | 0.2379926 |
| 5715.3 | 0.209690407 | 5715.34141 | 0.2552378 |

| | | | |
|---|---|---|---|
| 5715.4001 | 0.214966312 | 5715.68955 | 0.2748935 |
| 5715.50068 | 0.230179861 | 5716.03818 | 0.298223 |
| 5715.59932 | 0.23742342 | 5716.38535 | 0.3185287 |
| 5715.6999 | 0.243283018 | 5716.73252 | 0.3427659 |
| 5715.80049 | 0.251344651 | 5717.08066 | 0.3595758 |
| 5715.89961 | 0.253774881 | 5717.42979 | 0.3808123 |
| 5716.0002 | 0.261501819 | 5717.77695 | 0.4069308 |
| 5716.10029 | 0.273052543 | 5718.12412 | 0.4289525 |
| 5716.19941 | 0.275747567 | 5718.47129 | 0.448635 |
| 5716.3 | 0.285443038 | 5718.81992 | 0.473795 |
| 5716.40059 | 0.295432717 | 5719.16904 | 0.5063353 |
| 5716.49971 | 0.299446076 | 5719.5167 | 0.5376182 |
| 5716.5998 | 0.308333427 | 5719.86436 | 0.570718 |
| 5716.70039 | 0.31137225 | 5720.21152 | 0.6136792 |
| 5716.79951 | 0.320736647 | 5720.55918 | 0.6737393 |
| 5716.9001 | 0.324037552 | 5720.90732 | 0.7409493 |
| 5717.00068 | 0.334425777 | 5721.25498 | 0.8184614 |
| 5717.0998 | 0.342340112 | 5721.60264 | 0.8926835 |
| 5717.1999 | 0.345438004 | 5721.95078 | 0.9852142 |
| 5717.29951 | 0.354740888 | 5722.29844 | 1.0804772 |
| 5717.4001 | 0.36235559 | 5722.64658 | 1.1770638 |
| 5717.50068 | 0.36531958 | 5722.99473 | 1.2871255 |
| 5717.59932 | 0.373868316 | 5723.34287 | 1.3941501 |
| 5717.6999 | 0.377993733 | 5723.69102 | 1.4921398 |
| 5717.80049 | 0.382371277 | 5724.03916 | 1.5722889 |
| 5717.9001 | 0.388617188 | 5724.38633 | 1.6326327 |
| 5718.00068 | 0.397048384 | 5724.73252 | 1.6651584 |
| 5718.09932 | 0.396039575 | 5725.08066 | 1.6805487 |
| 5718.1999 | 0.40319851 | 5725.4293 | 1.681316 |
| 5718.29951 | 0.409281135 | 5725.77744 | 1.668518 |
| 5718.4001 | 0.41178298 | 5726.1251 | 1.6473641 |
| 5718.50068 | 0.424908519 | 5726.47178 | 1.6149554 |
| 5718.5998 | 0.428850502 | 5726.82041 | 1.5772694 |
| 5718.70039 | 0.437625885 | 5727.16904 | 1.5449256 |
| 5718.79951 | 0.435445398 | 5727.5167 | 1.5182161 |
| 5718.9001 | 0.440721422 | 5727.86338 | 1.4908981 |
| 5718.99971 | 0.443488508 | 5728.21201 | 1.4638424 |
| 5719.0998 | 0.448266119 | 5728.56016 | 1.4515438 |
| 5719.20039 | 0.459315926 | 5728.90684 | 1.4346134 |
| 5719.3 | 0.459327668 | 5729.25498 | 1.4217011 |
| 5719.40059 | 0.458854675 | 5729.60215 | 1.4078709 |
| 5719.5002 | 0.468288422 | 5729.95078 | 1.3984538 |
| 5719.60029 | 0.478925109 | 5730.29893 | 1.3914032 |

| | | | |
|---|---|---|---|
| 5719.6999 | 0.482789606 | 5730.64609 | 1.3848524 |
| 5719.80049 | 0.490297556 | 5730.99473 | 1.3755417 |
| 5719.9001 | 0.496398717 | 5731.34189 | 1.369418 |
| 5720.00068 | 0.502095222 | 5731.69053 | 1.36479 |
| 5720.09932 | 0.511559248 | 5732.0377 | 1.3578601 |
| 5720.20039 | 0.523370564 | 5732.38633 | 1.3558459 |
| 5720.29951 | 0.53346616 | 5732.73301 | 1.3628339 |
| 5720.40059 | 0.540799558 | 5733.08066 | 1.364082 |
| 5720.5002 | 0.560021579 | 5733.4293 | 1.3679162 |
| 5720.60029 | 0.563241899 | 5733.77695 | 1.3754467 |
| 5720.6999 | 0.578378439 | 5734.12559 | 1.3858581 |
| 5720.80049 | 0.588643312 | 5734.47227 | 1.399251 |
| 5720.9001 | 0.605754077 | 5734.81992 | 1.4147973 |
| 5721.00068 | 0.61965853 | 5735.16904 | 1.4206557 |
| 5721.0998 | 0.635289967 | 5735.51572 | 1.4178466 |
| 5721.19941 | 0.654179871 | 5735.86387 | 1.4092881 |
| 5721.3 | 0.673237026 | 5736.21299 | 1.3956556 |
| 5721.39961 | 0.688828409 | 5736.55967 | 1.3746733 |
| 5721.5002 | 0.710366488 | 5736.90684 | 1.3449186 |
| 5721.59932 | 0.735416412 | 5737.254 | 1.3130313 |
| 5721.70039 | 0.752966821 | 5737.60264 | 1.2800552 |
| 5721.8 | 0.778972626 | 5737.95176 | 1.244063 |
| 5721.90059 | 0.803634882 | 5738.29893 | 1.2099751 |
| 5722.0002 | 0.825638294 | 5738.64658 | 1.1726369 |
| 5722.09932 | 0.852842152 | 5738.99375 | 1.1361259 |
| 5722.1999 | 0.883112907 | 5739.34141 | 1.1138599 |
| 5722.29951 | 0.907142937 | 5739.68906 | 1.090803 |
| 5722.40059 | 0.927799881 | 5740.03672 | 1.0686368 |
| 5722.5002 | 0.966260374 | 5740.38438 | 1.0571767 |
| 5722.59932 | 0.994932473 | 5740.73203 | 1.0413772 |
| 5722.6999 | 1.01783264 | 5741.07969 | 1.0255916 |
| 5722.79951 | 1.05625618 | 5741.42783 | 1.0158135 |
| 5722.90059 | 1.08451641 | 5741.77549 | 1.0117108 |
| 5723.0002 | 1.11342466 | 5742.12363 | 1.0030488 |
| 5723.09932 | 1.14757383 | 5742.47178 | 0.9984958 |
| 5723.20039 | 1.1837647 | 5742.81992 | 0.9917546 |
| 5723.3 | 1.20494795 | 5743.16807 | 0.9892382 |
| 5723.39961 | 1.23859012 | 5743.68711 | 0.9866204 |
| 5723.5002 | 1.27262723 | 5744.85801 | 0.9783494 |
| 5723.5998 | 1.30123723 | 5746.67148 | 0.9708158 |
| 5723.70039 | 1.33492339 | 5748.5582 | 0.9665663 |
| 5723.8 | 1.36476958 | 5750.51963 | 0.9681707 |
| 5723.89961 | 1.38933647 | 5752.55479 | 0.9839451 |

| | | | |
|---|---|---|---|
| 5724.00068 | 1.41343963 | 5754.66367 | 0.9947466 |
| 5724.0998 | 1.44764709 | 5756.84482 | 1.0036501 |
| 5724.19941 | 1.47221601 | 5759.1002 | 1.009203 |
| 5724.30049 | 1.49960279 | 5761.42783 | 1.0085888 |
| 5724.4001 | 1.52747285 | 5763.83066 | 1.0047473 |
| 5724.5002 | 1.54460621 | 5766.30625 | 0.9991045 |
| 5724.60029 | 1.56731331 | 5768.85703 | 0.9945289 |
| 5724.6999 | 1.58073056 | 5771.48057 | 0.9863971 |
| 5724.8 | 1.59867895 | 5774.17734 | 0.9810573 |
| 5724.89961 | 1.61521375 | 5776.94736 | 0.9760618 |
| 5725.00068 | 1.63429499 | 5779.7916 | 0.9783381 |
| 5725.0998 | 1.65808058 | 5782.70811 | 0.9773257 |
| 5725.19941 | 1.66159332 | 5785.69834 | 0.9783736 |
| 5725.30049 | 1.67466366 | 5788.76328 | 0.9787685 |
| 5725.4001 | 1.69056559 | 5791.90195 | 0.9803028 |
| 5725.5002 | 1.69020224 | 5795.11387 | 0.9818565 |
| 5725.59932 | 1.6945926 | 5798.4 | 0.9730932 |
| 5725.70039 | 1.70378578 | 5801.75986 | 0.9737753 |
| 5725.8 | 1.70343554 | 5805.19297 | 0.9707429 |
| 5725.9001 | 1.69690478 | 5808.69834 | 0.9710169 |
| 5725.99971 | 1.69270766 | 5812.27744 | 0.9711415 |
| 5726.10029 | 1.69002903 | 5815.93223 | 0.9698167 |
| 5726.1999 | 1.68732035 | 5819.6583 | 0.9637923 |
| 5726.3 | 1.69391191 | 5823.45957 | 0.9676976 |
| 5726.39961 | 1.69487941 | 5827.33408 | 0.9897082 |
| 5726.50068 | 1.68231523 | 5831.28232 | 0.9985261 |
| 5726.5998 | 1.67312849 | 5835.30381 | 0.9987197 |
| 5726.6999 | 1.66447067 | 5839.39854 | 0.995077 |
| 5726.79951 | 1.65497017 | 5843.56748 | 0.9993772 |
| 5726.89961 | 1.64929783 | 5847.80869 | 1.0042126 |
| 5727.00068 | 1.64629936 | 5852.12607 | 1.0114734 |
| 5727.0998 | 1.63169897 | 5856.51426 | 1.004903 |
| 5727.1999 | 1.62719262 | 5860.97812 | 0.9988036 |
| 5727.29951 | 1.62789869 | 5865.51523 | 0.9970074 |
| 5727.39961 | 1.61279964 | | |
| 5727.50068 | 1.60584104 | | |
| 5727.5998 | 1.60266292 | | |
| 5727.6999 | 1.59585249 | | |
| 5727.8 | 1.59011304 | | |
| 5727.89961 | 1.58529532 | | |
| 5728.00068 | 1.57739937 | | |
| 5728.10029 | 1.56742668 | | |
| 5728.1999 | 1.56838512 | | |

| | | | |
|---|---|---|---|
| 5728.3 | 1.56657934 | | |
| 5728.4001 | 1.55174208 | | |
| 5728.49971 | 1.54753006 | | |
| 5728.59932 | 1.54925978 | | |
| 5728.70039 | 1.53962791 | | |
| 5728.80049 | 1.53682649 | | |
| 5728.9001 | 1.53729212 | | |
| 5729.0002 | 1.52904212 | | |
| 5729.0998 | 1.52236331 | | |
| 5729.1999 | 1.52329826 | | |
| 5729.29951 | 1.51590598 | | |
| 5729.39961 | 1.50968349 | | |
| 5729.50068 | 1.50893974 | | |
| 5729.60029 | 1.5004648 | | |
| 5729.70039 | 1.49304903 | | |
| 5729.8 | 1.49557436 | | |
| 5729.9001 | 1.49606407 | | |
| 5730.0002 | 1.48646736 | | |
| 5730.0998 | 1.47872066 | | |
| 5730.1999 | 1.47889149 | | |
| 5730.3 | 1.47174108 | | |
| 5730.39961 | 1.46604192 | | |
| 5730.49971 | 1.46723711 | | |
| 5730.59932 | 1.45552814 | | |
| 5730.69941 | 1.45110166 | | |
| 5730.79951 | 1.45618773 | | |
| 5730.90059 | 1.45226467 | | |
| 5731.00068 | 1.44344747 | | |
| 5731.10029 | 1.44661474 | | |
| 5731.20039 | 1.44309986 | | |
| 5731.30049 | 1.43804896 | | |
| 5731.40059 | 1.4372077 | | |
| 5731.50068 | 1.43989992 | | |
| 5731.60029 | 1.43438876 | | |
| 5731.70039 | 1.4348613 | | |
| 5731.80049 | 1.43321431 | | |
| 5732.00068 | 1.4266609 | | |
| 5732.20039 | 1.42920732 | | |
| 5732.40059 | 1.43107283 | | |
| 5732.60029 | 1.43037558 | | |
| 5732.80049 | 1.4270674 | | |
| 5732.99971 | 1.43488467 | | |
| 5733.19941 | 1.43571246 | | |

| | | | |
|---|---|---|---|
| 5733.4001 | 1.43542469 | | |
| 5733.5998 | 1.44153678 | | |
| 5733.8 | 1.45544779 | | |
| 5734.00068 | 1.45514357 | | |
| 5734.20039 | 1.46591485 | | |
| 5734.39961 | 1.47270787 | | |
| 5734.5998 | 1.47828484 | | |
| 5734.8 | 1.48874557 | | |
| 5735.00068 | 1.50004017 | | |
| 5735.19941 | 1.50462198 | | |
| 5735.4001 | 1.51627159 | | |
| 5735.60029 | 1.51250517 | | |
| 5735.79951 | 1.52053821 | | |
| 5736.0002 | 1.5218035 | | |
| 5736.20039 | 1.51670134 | | |
| 5736.39961 | 1.51532984 | | |
| 5736.5998 | 1.50053215 | | |
| 5736.79951 | 1.49271429 | | |
| 5737.0002 | 1.48734045 | | |
| 5737.20039 | 1.47107828 | | |
| 5737.4001 | 1.44932973 | | |
| 5737.60029 | 1.42646992 | | |
| 5737.8 | 1.40311587 | | |
| 5738.00068 | 1.38047886 | | |
| 5738.1999 | 1.35471058 | | |
| 5738.40059 | 1.3357892 | | |
| 5738.5998 | 1.30475712 | | |
| 5738.79951 | 1.28031647 | | |
| 5739.0002 | 1.25974834 | | |
| 5739.1999 | 1.23551023 | | |
| 5739.40059 | 1.21495771 | | |
| 5739.5998 | 1.19998038 | | |
| 5739.79951 | 1.17548096 | | |
| 5740.00068 | 1.15898418 | | |
| 5740.1999 | 1.15079391 | | |
| 5740.4001 | 1.13008273 | | |
| 5740.59932 | 1.11556482 | | |
| 5740.80049 | 1.10880148 | | |
| 5741.0002 | 1.09604895 | | |
| 5741.1999 | 1.08570802 | | |
| 5741.39961 | 1.07161641 | | |
| 5741.59932 | 1.06466782 | | |
| 5741.80049 | 1.05167198 | | |

| | | | |
|---|---|---|---|
| 5742.0002 | 1.0508939 | | |
| 5742.1999 | 1.04476821 | | |
| 5742.4001 | 1.03275359 | | |
| 5742.5998 | 1.02946353 | | |
| 5742.8 | 1.02871394 | | |
| 5742.99971 | 1.01788425 | | |
| 5743.19941 | 1.02016675 | | |
| 5743.39961 | 1.01533735 | | |
| 5743.59932 | 1.00645471 | | |
| 5743.79951 | 1.00620663 | | |
| 5745.79951 | 0.989976108 | | |
| 5747.80049 | 0.977128208 | | |
| 5749.79951 | 0.968251884 | | |
| 5749.90059 | 0.967899024 | | |
| 5750.0002 | 0.96755904 | | |
| 5750.0998 | 0.967223108 | | |
| 5750.19941 | 0.96690011 | | |
| 5750.30049 | 0.966580093 | | |
| 5750.40059 | 0.966271222 | | |
| 5750.5002 | 0.9659729 | | |
| 5750.5998 | 0.965677083 | | |
| 5750.69941 | 0.965394795 | | |
| 5750.80049 | 0.965114892 | | |
| 5750.90059 | 0.96484524 | | |
| 5751.0002 | 0.964586198 | | |
| 5751.0998 | 0.964328706 | | |
| 5751.19941 | 0.964084625 | | |
| 5751.30049 | 0.963843524 | | |
| 5751.40059 | 0.96361208 | | |
| 5751.5002 | 0.963388741 | | |
| 5751.5998 | 0.963168442 | | |
| 5751.6999 | 0.962959766 | | |
| 5751.79951 | 0.962757826 | | |
| 5751.89961 | 0.96256274 | | |
| 5752.00068 | 0.962371886 | | |
| 5752.0998 | 0.962188423 | | |
| 5752.1999 | 0.962013543 | | |
| 5752.3 | 0.961845458 | | |
| 5752.4001 | 0.961684167 | | |
| 5752.49971 | 0.961530626 | | |
| 5752.59932 | 0.961379051 | | |
| 5752.70039 | 0.961235702 | | |
| 5752.80049 | 0.961100042 | | |

| | | | |
|---|---|---|---|
| 5752.9001 | 0.96097213 | | |
| 5753.0002 | 0.960849524 | | |
| 5753.0998 | 0.960728884 | | |
| 5753.1999 | 0.960618794 | | |
| 5753.29951 | 0.960514605 | | |
| 5753.39961 | 0.960416794 | | |
| 5753.49971 | 0.960324109 | | |
| 5753.59932 | 0.960234463 | | |
| 5753.70039 | 0.960152566 | | |
| 5753.80049 | 0.960076809 | | |
| 5753.90059 | 0.960007489 | | |
| 5754.00068 | 0.959942877 | | |
| 5754.0998 | 0.959881246 | | |
| 5754.1999 | 0.95982784 | | |
| 5754.3 | 0.959780693 | | |
| 5754.4001 | 0.959737837 | | |
| 5756.4001 | 0.964720428 | | |
| 5758.40059 | 0.965539157 | | |
| 5760.39961 | 0.960518599 | | |
| 5762.39961 | 0.967659235 | | |
| 5767.4001 | 0.970997572 | | |
| 5772.4001 | 0.966919899 | | |
| 5777.4001 | 0.962209046 | | |
| 5782.40059 | 0.953148305 | | |
| 5787.4001 | 0.942896545 | | |
| 5792.39961 | 0.951727808 | | |
| 5797.39961 | 0.959930599 | | |
| 5802.40059 | 0.973468959 | | |
| 5812.39961 | 1.00078452 | | |
| 5822.39961 | 1.0082165 | | |
| 5832.4001 | 1.00416362 | | |
| 5842.4001 | 0.981871188 | | |
| 5852.39961 | 0.952466309 | | |
| 5862.4001 | 0.917500317 | | |
| 5872.4001 | 0.907691956 | | |
| 5882.39961 | 0.913750112 | | |
| 5892.4001 | 0.897524059 | | |

**Table S6.** Oxo XANES

| $[2\text{-}K]_4$ | | $[2\text{-}Rb]_4$ | | $[2\text{-}Cs]_4$ | |
|---|---|---|---|---|---|
| E(eV) | Normalized XANES | E(eV) | Normalized XANES | E(eV) | Normalized XANES |
| 5553.4 | 0.000476 | 5552.8 | -0.080109 | 5580.8 | 0.005732 |
| 5566.733 | -0.002077 | 5566.133 | -0.055195 | 5594.133 | 0.017919 |
| 5576.733 | -0.003133 | 5576.133 | -0.037129 | 5604.134 | 0.008316 |
| 5586.734 | -0.004066 | 5586.134 | -0.022134 | 5614.133 | -0.002494 |
| 5596.733 | -0.005338 | 5596.133 | -0.011923 | 5624.134 | -0.019095 |
| 5606.733 | -0.007347 | 5606.133 | -0.003561 | 5634.134 | -0.025827 |
| 5616.733 | -0.007811 | 5616.133 | 0.001686 | 5644.134 | -0.008683 |
| 5626.734 | -0.001829 | 5626.134 | 0.001543 | 5654.134 | 0.01791 |
| 5636.734 | 0.00139 | 5636.134 | 0.003969 | 5664.134 | 0.011979 |
| 5646.735 | 0.000844 | 5646.135 | 0.003169 | 5674.134 | -0.009776 |
| 5656.734 | -0.000417 | 5656.134 | 0.001949 | 5681.134 | -0.015569 |
| 5666.733 | 0.000335 | 5666.133 | -0.00019 | 5682.134 | -0.016407 |
| 5676.733 | 0.001041 | 5676.133 | -0.002941 | 5683.133 | -0.012074 |
| 5686.733 | -0.001787 | 5686.133 | -0.002296 | 5684.133 | -0.005491 |
| 5696.734 | 0.002336 | 5696.134 | 0.001261 | 5685.134 | -0.008989 |
| 5703.517 | 0.006991 | 5702.917 | 0.007171 | 5686.134 | -0.004935 |
| 5703.864 | 0.007857 | 5703.264 | 0.006378 | 5687.133 | -0.000686 |
| 5704.211 | 0.009321 | 5703.611 | 0.006408 | 5688.133 | 0.00323 |
| 5704.559 | 0.00818 | 5703.959 | 0.008331 | 5689.134 | 0.005849 |
| 5704.907 | 0.008227 | 5704.307 | 0.009291 | 5690.134 | 0.005295 |
| 5705.255 | 0.011128 | 5704.655 | 0.009587 | 5691.134 | 0.008426 |
| 5705.604 | 0.010938 | 5705.004 | 0.009073 | 5692.133 | 0.006598 |
| 5705.952 | 0.01104 | 5705.352 | 0.009993 | 5693.133 | 0.005928 |
| 5706.299 | 0.011727 | 5705.699 | 0.011424 | 5694.133 | 0.001985 |
| 5706.646 | 0.01239 | 5706.046 | 0.012566 | 5695.133 | -0.001176 |
| 5706.994 | 0.014905 | 5706.394 | 0.011103 | 5696.134 | 0.002399 |
| 5707.343 | 0.017406 | 5706.743 | 0.011637 | 5697.134 | 0.006703 |
| 5707.691 | 0.017381 | 5707.091 | 0.015389 | 5698.133 | 0.003797 |
| 5708.037 | 0.017481 | 5707.437 | 0.017343 | 5699.134 | 0.006204 |
| 5708.386 | 0.020215 | 5707.786 | 0.01665 | 5700.134 | 0.011486 |
| 5708.734 | 0.020929 | 5708.134 | 0.018457 | 5701.134 | 0.018906 |
| 5709.081 | 0.021407 | 5708.481 | 0.020048 | 5702.134 | 0.026016 |
| 5709.429 | 0.020995 | 5708.829 | 0.020593 | 5703.134 | 0.019247 |
| 5709.777 | 0.022814 | 5709.177 | 0.023218 | 5704.134 | 0.021831 |
| 5710.125 | 0.026519 | 5709.525 | 0.024199 | 5705.134 | 0.023488 |
| 5710.472 | 0.028737 | 5709.872 | 0.02663 | 5706.134 | 0.028648 |
| 5710.819 | 0.030651 | 5710.219 | 0.028873 | 5707.134 | 0.040452 |
| 5711.168 | 0.034009 | 5710.568 | 0.031253 | 5708.134 | 0.037461 |

| | | | | | |
|---|---|---|---|---|---|
| 5711.515 | 0.036013 | 5710.915 | 0.032693 | 5709.134 | 0.048357 |
| 5711.863 | 0.037325 | 5711.263 | 0.034605 | 5710.134 | 0.067114 |
| 5712.211 | 0.040576 | 5711.611 | 0.036422 | 5711.134 | 0.080663 |
| 5712.559 | 0.045114 | 5711.959 | 0.039193 | 5712.134 | 0.106996 |
| 5712.907 | 0.049056 | 5712.307 | 0.04152 | 5713.134 | 0.130847 |
| 5713.255 | 0.053285 | 5712.655 | 0.046082 | 5714.134 | 0.176037 |
| 5713.603 | 0.057392 | 5713.003 | 0.050807 | 5714.834 | 0.213753 |
| 5713.952 | 0.062567 | 5713.352 | 0.053557 | 5714.933 | 0.221367 |
| 5714.298 | 0.068287 | 5713.698 | 0.057936 | 5715.033 | 0.22624 |
| 5714.646 | 0.074742 | 5714.046 | 0.063567 | 5715.134 | 0.237909 |
| 5714.995 | 0.082088 | 5714.395 | 0.068532 | 5715.233 | 0.238908 |
| 5715.341 | 0.089547 | 5714.741 | 0.074754 | 5715.333 | 0.250808 |
| 5715.69 | 0.099113 | 5715.09 | 0.082258 | 5715.434 | 0.262275 |
| 5716.038 | 0.110665 | 5715.438 | 0.091794 | 5715.533 | 0.258143 |
| 5716.385 | 0.125418 | 5715.785 | 0.101741 | 5715.633 | 0.260582 |
| 5716.733 | 0.142086 | 5716.133 | 0.11263 | 5715.734 | 0.268208 |
| 5717.081 | 0.164001 | 5716.481 | 0.126743 | 5715.834 | 0.276092 |
| 5717.43 | 0.186346 | 5716.83 | 0.144007 | 5715.933 | 0.278345 |
| 5717.777 | 0.21269 | 5717.177 | 0.163557 | 5716.033 | 0.277782 |
| 5718.124 | 0.239447 | 5717.524 | 0.187004 | 5716.134 | 0.287019 |
| 5718.471 | 0.263634 | 5717.871 | 0.213096 | 5716.233 | 0.288367 |
| 5718.82 | 0.281787 | 5718.22 | 0.24094 | 5716.334 | 0.29884 |
| 5719.169 | 0.295115 | 5718.569 | 0.268787 | 5716.434 | 0.307243 |
| 5719.517 | 0.308481 | 5718.917 | 0.290553 | 5716.533 | 0.303298 |
| 5719.864 | 0.324451 | 5719.264 | 0.306966 | 5716.634 | 0.315755 |
| 5720.212 | 0.340368 | 5719.612 | 0.320071 | 5716.734 | 0.327543 |
| 5720.559 | 0.369942 | 5719.959 | 0.33412 | 5716.833 | 0.327922 |
| 5720.907 | 0.410598 | 5720.307 | 0.350776 | 5716.934 | 0.335042 |
| 5721.255 | 0.463915 | 5720.655 | 0.374837 | 5717.034 | 0.340224 |
| 5721.603 | 0.531464 | 5721.003 | 0.414294 | 5717.133 | 0.344308 |
| 5721.951 | 0.608729 | 5721.351 | 0.464011 | 5717.233 | 0.356106 |
| 5722.298 | 0.702486 | 5721.698 | 0.526454 | 5717.333 | 0.367595 |
| 5722.647 | 0.815706 | 5722.047 | 0.602908 | 5717.434 | 0.371824 |
| 5722.995 | 0.950403 | 5722.395 | 0.692135 | 5717.533 | 0.376879 |
| 5723.343 | 1.099569 | 5722.743 | 0.794062 | 5717.633 | 0.376941 |
| 5723.691 | 1.255883 | 5723.091 | 0.918674 | 5717.734 | 0.380509 |
| 5724.039 | 1.412268 | 5723.439 | 1.060228 | 5717.834 | 0.388629 |
| 5724.386 | 1.551092 | 5723.786 | 1.211113 | 5717.934 | 0.396659 |
| 5724.733 | 1.658202 | 5724.133 | 1.360502 | 5718.033 | 0.409822 |
| 5725.081 | 1.731318 | 5724.481 | 1.500445 | 5718.133 | 0.410405 |
| 5725.429 | 1.762056 | 5724.829 | 1.611737 | 5718.233 | 0.420074 |
| 5725.777 | 1.769419 | 5725.177 | 1.68977 | 5718.333 | 0.432472 |
| 5726.125 | 1.757159 | 5725.525 | 1.735255 | 5718.434 | 0.438113 |

| | | | | | |
|---|---|---|---|---|---|
| 5726.472 | 1.730071 | 5725.872 | 1.747001 | 5718.534 | 0.443873 |
| 5726.82 | 1.697215 | 5726.22 | 1.737201 | 5718.634 | 0.447098 |
| 5727.169 | 1.66005 | 5726.569 | 1.714112 | 5718.734 | 0.450978 |
| 5727.517 | 1.627344 | 5726.917 | 1.684017 | 5718.833 | 0.457269 |
| 5727.863 | 1.602185 | 5727.263 | 1.650517 | 5718.933 | 0.457001 |
| 5728.212 | 1.581861 | 5727.612 | 1.618511 | 5719.033 | 0.459791 |
| 5728.56 | 1.56983 | 5727.96 | 1.590784 | 5719.134 | 0.466647 |
| 5728.907 | 1.564195 | 5728.307 | 1.569069 | 5719.234 | 0.470765 |
| 5729.255 | 1.561617 | 5728.655 | 1.554449 | 5719.334 | 0.483204 |
| 5729.602 | 1.562024 | 5729.002 | 1.54656 | 5719.434 | 0.481171 |
| 5729.951 | 1.563073 | 5729.351 | 1.544293 | 5719.533 | 0.487569 |
| 5730.299 | 1.561921 | 5729.699 | 1.544436 | 5719.634 | 0.49563 |
| 5730.646 | 1.561921 | 5730.046 | 1.544728 | 5719.734 | 0.495784 |
| 5730.995 | 1.561939 | 5730.395 | 1.545029 | 5719.834 | 0.501219 |
| 5731.342 | 1.563581 | 5730.742 | 1.546556 | 5719.934 | 0.507296 |
| 5731.691 | 1.564645 | 5731.091 | 1.548702 | 5720.033 | 0.507547 |
| 5732.038 | 1.564179 | 5731.438 | 1.546247 | 5720.133 | 0.511991 |
| 5732.386 | 1.564028 | 5731.786 | 1.546534 | 5720.234 | 0.520364 |
| 5732.733 | 1.565904 | 5732.133 | 1.546886 | 5720.333 | 0.530392 |
| 5733.081 | 1.569206 | 5732.481 | 1.54731 | 5720.434 | 0.537387 |
| 5733.429 | 1.573127 | 5732.829 | 1.544732 | 5720.533 | 0.549662 |
| 5733.777 | 1.576962 | 5733.177 | 1.544918 | 5720.634 | 0.558647 |
| 5734.126 | 1.581876 | 5733.526 | 1.547623 | 5720.734 | 0.564413 |
| 5734.472 | 1.587765 | 5733.872 | 1.54779 | 5720.834 | 0.581314 |
| 5734.82 | 1.595114 | 5734.22 | 1.552415 | 5720.934 | 0.59673 |
| 5735.169 | 1.601591 | 5734.569 | 1.558968 | 5721.034 | 0.605524 |
| 5735.516 | 1.605913 | 5734.916 | 1.565097 | 5721.133 | 0.618695 |
| 5735.864 | 1.602207 | 5735.264 | 1.57179 | 5721.233 | 0.638519 |
| 5736.213 | 1.589301 | 5735.613 | 1.577896 | 5721.333 | 0.650069 |
| 5736.56 | 1.570351 | 5735.96 | 1.578688 | 5721.433 | 0.667079 |
| 5736.907 | 1.541588 | 5736.307 | 1.572904 | 5721.533 | 0.690135 |
| 5737.254 | 1.502679 | 5736.654 | 1.56041 | 5721.633 | 0.706928 |
| 5737.603 | 1.45811 | 5737.003 | 1.537252 | 5721.734 | 0.729489 |
| 5737.952 | 1.410862 | 5737.352 | 1.503571 | 5721.834 | 0.749473 |
| 5738.299 | 1.364077 | 5737.699 | 1.465102 | 5721.934 | 0.768547 |
| 5738.647 | 1.322997 | 5738.047 | 1.422101 | 5722.033 | 0.790766 |
| 5738.994 | 1.28447 | 5738.394 | 1.378496 | 5722.133 | 0.819031 |
| 5739.341 | 1.247682 | 5738.741 | 1.337031 | 5722.233 | 0.842396 |
| 5739.689 | 1.214985 | 5739.089 | 1.300914 | 5722.333 | 0.867387 |
| 5740.037 | 1.18511 | 5739.437 | 1.264432 | 5722.434 | 0.893155 |
| 5740.384 | 1.158366 | 5739.784 | 1.230975 | 5722.533 | 0.919407 |
| 5740.732 | 1.136298 | 5740.132 | 1.204698 | 5722.633 | 0.94916 |
| 5741.08 | 1.117085 | 5740.48 | 1.178449 | 5722.733 | 0.987932 |

| | | | | | |
|---|---|---|---|---|---|
| 5741.428 | 1.100967 | 5740.828 | 1.155834 | 5722.833 | 1.018716 |
| 5741.775 | 1.086725 | 5741.175 | 1.136491 | 5722.934 | 1.039835 |
| 5742.124 | 1.075088 | 5741.524 | 1.120043 | 5723.033 | 1.072076 |
| 5742.472 | 1.065626 | 5741.872 | 1.10674 | 5723.133 | 1.109362 |
| 5742.82 | 1.055723 | 5742.22 | 1.096278 | 5723.234 | 1.135922 |
| 5743.168 | 1.048049 | 5742.568 | 1.086616 | 5723.333 | 1.171017 |
| 5743.687 | 1.039913 | 5743.087 | 1.076034 | 5723.433 | 1.202678 |
| 5744.858 | 1.023532 | 5744.258 | 1.059987 | 5723.533 | 1.242282 |
| 5746.671 | 1.000406 | 5746.071 | 1.040727 | 5723.634 | 1.282989 |
| 5748.558 | 0.982379 | 5747.958 | 1.022846 | 5723.734 | 1.316873 |
| 5750.52 | 0.972974 | 5749.92 | 1.007486 | 5723.833 | 1.35731 |
| 5752.555 | 0.971075 | 5751.955 | 1.005119 | 5723.933 | 1.383112 |
| 5754.664 | 0.97731 | 5754.064 | 1.010212 | 5724.034 | 1.419658 |
| 5756.845 | 0.987532 | 5756.245 | 1.018039 | 5724.133 | 1.455518 |
| 5759.1 | 0.993945 | 5758.5 | 1.026913 | 5724.233 | 1.476097 |
| 5761.428 | 0.992466 | 5760.828 | 1.030277 | 5724.334 | 1.506149 |
| 5763.831 | 0.986057 | 5763.231 | 1.029534 | 5724.433 | 1.538888 |
| 5766.306 | 0.981637 | 5765.706 | 1.028663 | 5724.533 | 1.565221 |
| 5768.857 | 0.980636 | 5768.257 | 1.029393 | 5724.634 | 1.585618 |
| 5771.481 | 0.976972 | 5770.881 | 1.030893 | 5724.733 | 1.613667 |
| 5774.177 | 0.972305 | 5773.577 | 1.029657 | 5724.833 | 1.629822 |
| 5776.947 | 0.965034 | 5776.347 | 1.025287 | 5724.933 | 1.648904 |
| 5779.792 | 0.960408 | 5779.192 | 1.022876 | 5725.034 | 1.675938 |
| 5782.708 | 0.956648 | 5782.108 | 1.021424 | 5725.133 | 1.689323 |
| 5785.698 | 0.9539 | 5785.098 | 1.01983 | 5725.233 | 1.696712 |
| 5788.763 | 0.948948 | 5788.163 | 1.017985 | 5725.334 | 1.714897 |
| 5791.902 | 0.941102 | 5791.302 | 1.015634 | 5725.433 | 1.720275 |
| 5795.114 | 0.933381 | 5794.514 | 1.016316 | 5725.533 | 1.71421 |
| 5798.4 | 0.92836 | 5797.8 | 1.019973 | 5725.633 | 1.716681 |
| 5801.76 | 0.926718 | 5801.16 | 1.020782 | 5725.734 | 1.720521 |
| 5805.193 | 0.927131 | 5804.593 | 1.022527 | 5725.833 | 1.703287 |
| 5808.698 | 0.928651 | 5808.098 | 1.024518 | 5725.933 | 1.702634 |
| 5812.277 | 0.928813 | 5811.677 | 1.02795 | 5726.033 | 1.704493 |
| 5815.932 | 0.927582 | 5815.332 | 1.031231 | 5726.134 | 1.700124 |
| 5819.658 | 0.922692 | 5819.058 | 1.031555 | 5726.233 | 1.698964 |
| 5823.46 | 0.917394 | 5822.86 | 1.01417 | 5726.333 | 1.696187 |
| 5827.334 | 0.919198 | 5826.734 | 1.00715 | 5726.433 | 1.685768 |
| 5831.282 | 0.914171 | 5830.682 | 1.061846 | 5726.534 | 1.670239 |
| 5835.304 | 0.90783 | 5834.704 | 1.056891 | 5726.633 | 1.662587 |
| 5839.399 | 0.905004 | 5838.799 | 1.054824 | 5726.733 | 1.661234 |
| 5843.567 | 0.900143 | 5842.967 | 1.05538 | 5726.833 | 1.648236 |
| 5847.809 | 0.898856 | 5847.209 | 1.058034 | 5726.933 | 1.642114 |
| 5852.126 | 0.898478 | 5851.526 | 1.063342 | 5727.034 | 1.63179 |

| | | | | | |
|---|---|---|---|---|---|
| 5856.514 | 0.889997 | 5855.914 | 1.06688 | 5727.133 | 1.616195 |
| 5860.978 | 0.876597 | 5860.378 | 1.062914 | 5727.233 | 1.616102 |
| 5865.515 | 0.860713 | 5864.915 | 1.052942 | 5727.333 | 1.618769 |
| | | | | 5727.433 | 1.610327 |
| | | | | 5727.534 | 1.599272 |
| | | | | 5727.633 | 1.597066 |
| | | | | 5727.733 | 1.587063 |
| | | | | 5727.833 | 1.578516 |
| | | | | 5727.933 | 1.573931 |
| | | | | 5728.034 | 1.570441 |
| | | | | 5728.134 | 1.56212 |
| | | | | 5728.233 | 1.560304 |
| | | | | 5728.333 | 1.552845 |
| | | | | 5728.433 | 1.539068 |
| | | | | 5728.533 | 1.541646 |
| | | | | 5728.633 | 1.547917 |
| | | | | 5728.734 | 1.543504 |
| | | | | 5728.834 | 1.540568 |
| | | | | 5728.933 | 1.531457 |
| | | | | 5729.033 | 1.525226 |
| | | | | 5729.133 | 1.528157 |
| | | | | 5729.233 | 1.527724 |
| | | | | 5729.333 | 1.523528 |
| | | | | 5729.433 | 1.520134 |
| | | | | 5729.534 | 1.517618 |
| | | | | 5729.634 | 1.518743 |
| | | | | 5729.734 | 1.520977 |
| | | | | 5729.833 | 1.519147 |
| | | | | 5729.933 | 1.525089 |
| | | | | 5730.033 | 1.518249 |
| | | | | 5730.133 | 1.515064 |
| | | | | 5730.233 | 1.522068 |
| | | | | 5730.333 | 1.516513 |
| | | | | 5730.433 | 1.514506 |
| | | | | 5730.533 | 1.52129 |
| | | | | 5730.633 | 1.516739 |
| | | | | 5730.733 | 1.508293 |
| | | | | 5730.833 | 1.507442 |
| | | | | 5730.934 | 1.50896 |
| | | | | 5731.034 | 1.503192 |
| | | | | 5731.134 | 1.502806 |
| | | | | 5731.234 | 1.508041 |
| | | | | 5731.334 | 1.497918 |

| | | | | | |
|---|---|---|---|---|---|
| | | | | 5731.434 | 1.503034 |
| | | | | 5731.534 | 1.504097 |
| | | | | 5731.634 | 1.498856 |
| | | | | 5731.734 | 1.49855 |
| | | | | 5731.867 | 1.501114 |
| | | | | 5732.067 | 1.498003 |
| | | | | 5732.267 | 1.504026 |
| | | | | 5732.467 | 1.499274 |
| | | | | 5732.667 | 1.496071 |
| | | | | 5732.867 | 1.491299 |
| | | | | 5733.066 | 1.494343 |
| | | | | 5733.266 | 1.500492 |
| | | | | 5733.467 | 1.48923 |
| | | | | 5733.667 | 1.491092 |
| | | | | 5733.867 | 1.500414 |
| | | | | 5734.067 | 1.49612 |
| | | | | 5734.267 | 1.502469 |
| | | | | 5734.467 | 1.508572 |
| | | | | 5734.667 | 1.507013 |
| | | | | 5734.867 | 1.51149 |
| | | | | 5735.067 | 1.524536 |
| | | | | 5735.266 | 1.531105 |
| | | | | 5735.467 | 1.5343 |
| | | | | 5735.667 | 1.535054 |
| | | | | 5735.866 | 1.533388 |
| | | | | 5736.067 | 1.529647 |
| | | | | 5736.267 | 1.529832 |
| | | | | 5736.467 | 1.527734 |
| | | | | 5736.666 | 1.513412 |
| | | | | 5736.866 | 1.506505 |
| | | | | 5737.067 | 1.499971 |
| | | | | 5737.267 | 1.474427 |
| | | | | 5737.467 | 1.460395 |
| | | | | 5737.667 | 1.43899 |
| | | | | 5737.867 | 1.415917 |
| | | | | 5738.067 | 1.401127 |
| | | | | 5738.267 | 1.388301 |
| | | | | 5738.467 | 1.368712 |
| | | | | 5738.666 | 1.346047 |
| | | | | 5738.866 | 1.328413 |
| | | | | 5739.067 | 1.315083 |
| | | | | 5739.267 | 1.297405 |
| | | | | 5739.467 | 1.28241 |

| | | | | | |
|---|---|---|---|---|---|
| | | | | 5739.666 | 1.272414 |
| | | | | 5739.866 | 1.25755 |
| | | | | 5740.067 | 1.24978 |
| | | | | 5740.267 | 1.234044 |
| | | | | 5740.467 | 1.210051 |
| | | | | 5740.666 | 1.20606 |
| | | | | 5740.867 | 1.202762 |
| | | | | 5741.067 | 1.188903 |
| | | | | 5741.266 | 1.177373 |
| | | | | 5741.466 | 1.167809 |
| | | | | 5741.666 | 1.158174 |
| | | | | 5741.867 | 1.148856 |
| | | | | 5742.067 | 1.1559 |
| | | | | 5742.267 | 1.153764 |
| | | | | 5742.467 | 1.129145 |
| | | | | 5742.667 | 1.125158 |
| | | | | 5742.866 | 1.123984 |
| | | | | 5743.066 | 1.114546 |
| | | | | 5743.266 | 1.111874 |
| | | | | 5743.466 | 1.104287 |
| | | | | 5743.666 | 1.08864 |
| | | | | 5744.466 | 1.078265 |
| | | | | 5746.467 | 1.060916 |
| | | | | 5748.467 | 1.04529 |
| | | | | 5749.833 | 1.048733 |
| | | | | 5749.934 | 1.04869 |
| | | | | 5750.033 | 1.048658 |
| | | | | 5750.133 | 1.048639 |
| | | | | 5750.233 | 1.048637 |
| | | | | 5750.334 | 1.048643 |
| | | | | 5750.434 | 1.048666 |
| | | | | 5750.533 | 1.048699 |
| | | | | 5750.633 | 1.048741 |
| | | | | 5750.733 | 1.0488 |
| | | | | 5750.834 | 1.048868 |
| | | | | 5750.934 | 1.048953 |
| | | | | 5751.033 | 1.049044 |
| | | | | 5751.133 | 1.049145 |
| | | | | 5751.233 | 1.049261 |
| | | | | 5751.334 | 1.049386 |
| | | | | 5751.434 | 1.049527 |
| | | | | 5751.533 | 1.049673 |
| | | | | 5751.633 | 1.049827 |

| | | | | | |
|---|---|---|---|---|---|
| | | | | 5751.733 | 1.049998 |
| | | | | 5751.833 | 1.050176 |
| | | | | 5751.933 | 1.050368 |
| | | | | 5752.034 | 1.05057 |
| | | | | 5752.133 | 1.050771 |
| | | | | 5752.233 | 1.05099 |
| | | | | 5752.333 | 1.051219 |
| | | | | 5752.433 | 1.051459 |
| | | | | 5752.533 | 1.051703 |
| | | | | 5752.633 | 1.051956 |
| | | | | 5752.734 | 1.052222 |
| | | | | 5752.834 | 1.052496 |
| | | | | 5752.933 | 1.052775 |
| | | | | 5753.033 | 1.053066 |
| | | | | 5753.133 | 1.053358 |
| | | | | 5753.233 | 1.053665 |
| | | | | 5753.333 | 1.053977 |
| | | | | 5753.433 | 1.054297 |
| | | | | 5753.533 | 1.054626 |
| | | | | 5753.633 | 1.05496 |
| | | | | 5753.734 | 1.055305 |
| | | | | 5753.834 | 1.055655 |
| | | | | 5753.934 | 1.056013 |
| | | | | 5754.034 | 1.056417 |
| | | | | 5754.133 | 1.056867 |
| | | | | 5754.233 | 1.057262 |
| | | | | 5754.333 | 1.057651 |
| | | | | 5755.067 | 1.060051 |
| | | | | 5757.067 | 1.065727 |
| | | | | 5759.067 | 1.069218 |
| | | | | 5761.066 | 1.08209 |
| | | | | 5764.067 | 1.09813 |
| | | | | 5769.067 | 1.078563 |
| | | | | 5774.067 | 1.059375 |
| | | | | 5779.067 | 1.035758 |
| | | | | 5784.067 | 1.013829 |
| | | | | 5789.067 | 1.001376 |
| | | | | 5794.066 | 0.996816 |
| | | | | 5799.067 | 1.010769 |
| | | | | 5805.734 | 1.045539 |
| | | | | 5815.733 | 1.06009 |
| | | | | 5825.733 | 1.06314 |
| | | | | 5835.734 | 1.075152 |

| | | | | | |
|---|---|---|---|---|---|
| | | | | 5845.733 | 1.074646 |
| | | | | 5855.733 | 1.034558 |
| | | | | 5865.734 | 1.010658 |
| | | | | 5875.733 | 1.034984 |
| | | | | 5885.733 | 1.079686 |

**Table S7.** Anilide HERFD

| **1-H** | |
|---|---|
| E(eV) | Normalized HERFD |
| 5680 | 7.28E-05 |
| 5681.334 | 2.81E-05 |
| 5682.333 | 1.46E-04 |
| 5683.333 | -2.96E-04 |
| 5684.334 | -2.95E-04 |
| 5685.334 | -1.65E-04 |
| 5686.333 | -1.17E-05 |
| 5687.333 | 1.07E-04 |
| 5688.334 | -4.35E-05 |
| 5689.334 | -1.71E-04 |
| 5690.334 | -9.89E-05 |
| 5691.333 | 6.74E-05 |
| 5692.333 | 1.99E-04 |
| 5693.333 | 2.82E-04 |
| 5694.333 | -3.35E-05 |
| 5695.334 | 3.10E-04 |
| 5696.334 | 1.74E-05 |
| 5697.333 | -1.70E-04 |
| 5698.334 | -3.85E-05 |
| 5699.334 | 1.64E-04 |
| 5700.334 | 2.36E-05 |
| 5701.334 | 8.38E-05 |
| 5702.334 | -4.01E-04 |
| 5703.334 | -2.70E-04 |
| 5704.334 | -2.45E-04 |
| 5705.334 | -1.94E-05 |
| 5706.334 | 2.42E-04 |
| 5707.334 | -3.08E-05 |
| 5708.334 | 2.78E-04 |
| 5709.334 | 4.07E-04 |
| 5710.334 | 2.52E-04 |
| 5711.334 | 1.68E-04 |

| | |
|---|---|
| 5712.334 | 1.19E-04 |
| 5713.334 | 3.79E-04 |
| 5714.034 | 1.05E-03 |
| 5714.133 | 1.40E-03 |
| 5714.233 | 1.98E-03 |
| 5714.334 | 1.92E-03 |
| 5714.433 | 1.43E-03 |
| 5714.533 | 1.63E-03 |
| 5714.634 | 1.79E-03 |
| 5714.733 | 2.35E-03 |
| 5714.833 | 1.96E-03 |
| 5714.934 | 2.14E-03 |
| 5715.034 | 2.87E-03 |
| 5715.133 | 3.32E-03 |
| 5715.233 | 3.65E-03 |
| 5715.334 | 4.17E-03 |
| 5715.433 | 5.36E-03 |
| 5715.534 | 5.83E-03 |
| 5715.634 | 6.63E-03 |
| 5715.733 | 8.29E-03 |
| 5715.834 | 1.07E-02 |
| 5715.934 | 1.22E-02 |
| 5716.033 | 1.33E-02 |
| 5716.134 | 1.63E-02 |
| 5716.234 | 1.93E-02 |
| 5716.333 | 2.40E-02 |
| 5716.433 | 2.98E-02 |
| 5716.533 | 3.61E-02 |
| 5716.634 | 4.35E-02 |
| 5716.733 | 5.42E-02 |
| 5716.833 | 6.52E-02 |
| 5716.934 | 7.69E-02 |
| 5717.034 | 9.00E-02 |
| 5717.134 | 0.102022 |
| 5717.233 | 0.115622 |
| 5717.333 | 0.130704 |
| 5717.433 | 0.140945 |
| 5717.533 | 0.15054 |
| 5717.634 | 0.157275 |
| 5717.734 | 0.159842 |
| 5717.834 | 0.157776 |
| 5717.934 | 0.156474 |
| 5718.033 | 0.159187 |

| | |
|---|---|
| 5718.133 | 0.153938 |
| 5718.233 | 0.146258 |
| 5718.334 | 0.142568 |
| 5718.434 | 0.130082 |
| 5718.534 | 0.11974 |
| 5718.634 | 0.112325 |
| 5718.733 | 0.105138 |
| 5718.834 | 9.73E-02 |
| 5718.934 | 8.99E-02 |
| 5719.034 | 8.30E-02 |
| 5719.134 | 7.83E-02 |
| 5719.233 | 7.59E-02 |
| 5719.333 | 7.56E-02 |
| 5719.434 | 7.22E-02 |
| 5719.533 | 7.01E-02 |
| 5719.634 | 6.97E-02 |
| 5719.733 | 6.97E-02 |
| 5719.834 | 7.21E-02 |
| 5719.934 | 7.48E-02 |
| 5720.034 | 7.89E-02 |
| 5720.134 | 8.31E-02 |
| 5720.234 | 8.85E-02 |
| 5720.333 | 9.38E-02 |
| 5720.433 | 0.101629 |
| 5720.533 | 0.110645 |
| 5720.633 | 0.117395 |
| 5720.733 | 0.132165 |
| 5720.833 | 0.142669 |
| 5720.934 | 0.158909 |
| 5721.034 | 0.177477 |
| 5721.134 | 0.197695 |
| 5721.233 | 0.221093 |
| 5721.333 | 0.246642 |
| 5721.433 | 0.276916 |
| 5721.533 | 0.312625 |
| 5721.634 | 0.352984 |
| 5721.733 | 0.399332 |
| 5721.833 | 0.447363 |
| 5721.933 | 0.507087 |
| 5722.033 | 0.570816 |
| 5722.134 | 0.646345 |
| 5722.233 | 0.717874 |
| 5722.333 | 0.78806 |

| | |
|---|---|
| 5722.434 | 0.863923 |
| 5722.533 | 0.944593 |
| 5722.633 | 1.015084 |
| 5722.733 | 1.097348 |
| 5722.834 | 1.159422 |
| 5722.934 | 1.21847 |
| 5723.033 | 1.2768 |
| 5723.133 | 1.328766 |
| 5723.234 | 1.375749 |
| 5723.333 | 1.423115 |
| 5723.433 | 1.461281 |
| 5723.534 | 1.508267 |
| 5723.633 | 1.548771 |
| 5723.733 | 1.585034 |
| 5723.834 | 1.626981 |
| 5723.933 | 1.670224 |
| 5724.033 | 1.712996 |
| 5724.133 | 1.762062 |
| 5724.234 | 1.819186 |
| 5724.333 | 1.875766 |
| 5724.433 | 1.920633 |
| 5724.534 | 1.960664 |
| 5724.633 | 2.004492 |
| 5724.733 | 2.04064 |
| 5724.833 | 2.084447 |
| 5724.934 | 2.111756 |
| 5725.033 | 2.140803 |
| 5725.133 | 2.159092 |
| 5725.233 | 2.172897 |
| 5725.334 | 2.188952 |
| 5725.433 | 2.184391 |
| 5725.533 | 2.179174 |
| 5725.633 | 2.182233 |
| 5725.734 | 2.156125 |
| 5725.833 | 2.145373 |
| 5725.933 | 2.142239 |
| 5726.033 | 2.119534 |
| 5726.133 | 2.09846 |
| 5726.234 | 2.081596 |
| 5726.333 | 2.056989 |
| 5726.433 | 2.032575 |
| 5726.533 | 2.018249 |
| 5726.633 | 1.993377 |

| | |
|---|---|
| 5726.734 | 1.962407 |
| 5726.833 | 1.953711 |
| 5726.933 | 1.935093 |
| 5727.033 | 1.910469 |
| 5727.133 | 1.903261 |
| 5727.234 | 1.885113 |
| 5727.334 | 1.865506 |
| 5727.433 | 1.853382 |
| 5727.533 | 1.829172 |
| 5727.633 | 1.825792 |
| 5727.733 | 1.814546 |
| 5727.833 | 1.800968 |
| 5727.934 | 1.798923 |
| 5728.034 | 1.791247 |
| 5728.133 | 1.779584 |
| 5728.233 | 1.777978 |
| 5728.333 | 1.761229 |
| 5728.433 | 1.748837 |
| 5728.533 | 1.749079 |
| 5728.633 | 1.748516 |
| 5728.734 | 1.749517 |
| 5728.834 | 1.740626 |
| 5728.934 | 1.72621 |
| 5729.033 | 1.713507 |
| 5729.133 | 1.70146 |
| 5729.233 | 1.700055 |
| 5729.333 | 1.706289 |
| 5729.433 | 1.686083 |
| 5729.533 | 1.66375 |
| 5729.633 | 1.667309 |
| 5729.733 | 1.657463 |
| 5729.833 | 1.64698 |
| 5729.933 | 1.64854 |
| 5730.033 | 1.646244 |
| 5730.134 | 1.624619 |
| 5730.234 | 1.612739 |
| 5730.334 | 1.590956 |
| 5730.434 | 1.587517 |
| 5730.534 | 1.59011 |
| 5730.634 | 1.579417 |
| 5730.734 | 1.569637 |
| 5730.834 | 1.561753 |
| 5730.934 | 1.554089 |

| | |
|---|---|
| 5731.067 | 1.535802 |
| 5731.267 | 1.523669 |
| 5731.467 | 1.511441 |
| 5731.667 | 1.493118 |
| 5731.867 | 1.475056 |
| 5732.067 | 1.468055 |
| 5732.266 | 1.459805 |
| 5732.466 | 1.458733 |
| 5732.667 | 1.448264 |
| 5732.867 | 1.447546 |
| 5733.067 | 1.450201 |
| 5733.267 | 1.460603 |
| 5733.467 | 1.458588 |
| 5733.667 | 1.464659 |
| 5733.867 | 1.488579 |
| 5734.067 | 1.499722 |
| 5734.267 | 1.510048 |
| 5734.466 | 1.530127 |
| 5734.667 | 1.551457 |
| 5734.867 | 1.573941 |
| 5735.066 | 1.599171 |
| 5735.267 | 1.629361 |
| 5735.467 | 1.666283 |
| 5735.667 | 1.713218 |
| 5735.866 | 1.744255 |
| 5736.066 | 1.783895 |
| 5736.267 | 1.832251 |
| 5736.467 | 1.84071 |
| 5736.667 | 1.851682 |
| 5736.867 | 1.849597 |
| 5737.067 | 1.839702 |
| 5737.267 | 1.822536 |
| 5737.467 | 1.810525 |
| 5737.667 | 1.784162 |
| 5737.866 | 1.751368 |
| 5738.066 | 1.71324 |
| 5738.267 | 1.688816 |
| 5738.467 | 1.640032 |
| 5738.667 | 1.581497 |
| 5738.866 | 1.514639 |
| 5739.066 | 1.443954 |
| 5739.267 | 1.376057 |
| 5739.467 | 1.301672 |

| | |
|---|---|
| 5739.667 | 1.243721 |
| 5739.866 | 1.183975 |
| 5740.067 | 1.129043 |
| 5740.267 | 1.079478 |
| 5740.466 | 1.04648 |
| 5740.666 | 1.028243 |
| 5740.866 | 1.009819 |
| 5741.067 | 0.992767 |
| 5741.267 | 0.983969 |
| 5741.467 | 0.982594 |
| 5741.667 | 0.979935 |
| 5741.867 | 0.97349 |
| 5742.066 | 0.973258 |
| 5742.266 | 0.962822 |
| 5742.466 | 0.959315 |
| 5742.666 | 0.959314 |
| 5742.866 | 0.960009 |
| 5743.666 | 0.959014 |
| 5745.667 | 0.948332 |
| 5747.667 | 0.924557 |
| 5749.033 | 0.919449 |
| 5749.134 | 0.919643 |
| 5749.233 | 0.923983 |
| 5749.333 | 0.924754 |
| 5749.433 | 0.924037 |
| 5749.534 | 0.916679 |
| 5749.634 | 0.921748 |
| 5749.733 | 0.92413 |
| 5749.833 | 0.926852 |
| 5749.933 | 0.925516 |
| 5750.034 | 0.929226 |
| 5750.134 | 0.927138 |
| 5750.233 | 0.924887 |
| 5750.333 | 0.916555 |
| 5750.433 | 0.919358 |
| 5750.534 | 0.929315 |
| 5750.634 | 0.927749 |
| 5750.733 | 0.929477 |
| 5750.833 | 0.92503 |
| 5750.933 | 0.926414 |
| 5751.033 | 0.931929 |
| 5751.133 | 0.938724 |
| 5751.234 | 0.944052 |

| | |
|---|---|
| 5751.333 | 0.936396 |
| 5751.433 | 0.936954 |
| 5751.533 | 0.9456 |
| 5751.633 | 0.944524 |
| 5751.733 | 0.941633 |
| 5751.833 | 0.941013 |
| 5751.934 | 0.937507 |
| 5752.034 | 0.944743 |
| 5752.133 | 0.949299 |
| 5752.233 | 0.943055 |
| 5752.966 | 0.946605 |
| 5754.966 | 0.977253 |
| 5756.967 | 1.000007 |
| 5758.967 | 1.018707 |
| 5761.966 | 1.036695 |
| 5766.966 | 1.060373 |
| 5771.967 | 1.085736 |
| 5776.967 | 1.085705 |
| 5781.966 | 1.06936 |
| 5786.967 | 1.059128 |
| 5791.967 | 1.048246 |
| 5796.966 | 1.040592 |
| 5803.633 | 1.050281 |
| 5813.633 | 1.071258 |
| 5823.633 | 1.097403 |
| 5833.633 | 1.111549 |

**Table S8.** Imido HERFD

| **1-K** | | **1-Rb** | | **1-Cs** | |
|---|---|---|---|---|---|
| E(eV) | Normalized HERFD | E(eV) | Normalized HERFD | E(eV) | Normalized HERFD |
| 5680 | 4.99E-04 | 5680 | 1.27E-04 | 5680 | 2.59E-04 |
| 5681.334 | -2.30E-04 | 5681.334 | -3.26E-05 | 5681.334 | 7.01E-05 |
| 5682.333 | 2.92E-05 | 5682.333 | -3.57E-05 | 5682.333 | 1.58E-04 |
| 5683.333 | 8.92E-05 | 5683.333 | -1.43E-04 | 5683.333 | -1.61E-04 |
| 5684.334 | -5.99E-04 | 5684.334 | 4.25E-05 | 5684.334 | 2.07E-04 |
| 5685.334 | -1.10E-04 | 5685.334 | -2.55E-05 | 5685.334 | 3.38E-04 |
| 5686.333 | 4.33E-04 | 5686.333 | 3.06E-05 | 5686.333 | -4.96E-04 |
| 5687.333 | 3.83E-04 | 5687.333 | -6.22E-05 | 5687.333 | -3.86E-04 |
| 5688.334 | 4.14E-04 | 5688.334 | -8.98E-05 | 5688.334 | -6.84E-04 |

| | | | | | |
|---|---|---|---|---|---|
| 5689.334 | -1.09E-05 | 5689.334 | 1.92E-04 | 5689.334 | -2.08E-04 |
| 5690.334 | -8.41E-04 | 5690.334 | 7.12E-05 | 5690.334 | -1.62E-04 |
| 5691.333 | -3.25E-04 | 5691.333 | 7.78E-05 | 5691.333 | 7.76E-05 |
| 5692.333 | 1.10E-04 | 5692.333 | 2.18E-04 | 5692.333 | 5.92E-05 |
| 5693.333 | 4.41E-04 | 5693.333 | 1.27E-04 | 5693.333 | -2.62E-06 |
| 5694.333 | 4.95E-04 | 5694.333 | 1.95E-05 | 5694.333 | 6.57E-05 |
| 5695.334 | 2.67E-04 | 5695.334 | -3.68E-04 | 5695.334 | 6.53E-04 |
| 5696.334 | 6.44E-04 | 5696.334 | -1.40E-04 | 5696.334 | 6.13E-04 |
| 5697.333 | -2.41E-04 | 5697.333 | 1.59E-04 | 5697.333 | 1.40E-04 |
| 5698.334 | -5.84E-04 | 5698.334 | 5.63E-05 | 5698.334 | -2.69E-04 |
| 5699.334 | -5.31E-04 | 5699.334 | -1.34E-04 | 5699.334 | -1.14E-04 |
| 5700.334 | -4.13E-05 | 5700.334 | -1.45E-04 | 5700.334 | -3.94E-04 |
| 5701.334 | -3.23E-04 | 5701.334 | 4.34E-05 | 5701.334 | 2.60E-04 |
| 5702.334 | -4.80E-04 | 5702.334 | -1.68E-04 | 5702.334 | 5.44E-04 |
| 5703.334 | -2.64E-04 | 5703.334 | -1.16E-04 | 5703.334 | 4.57E-05 |
| 5704.334 | -2.42E-04 | 5704.334 | -1.83E-04 | 5704.334 | 2.58E-05 |
| 5705.334 | -2.16E-04 | 5705.334 | -3.58E-05 | 5705.334 | 1.79E-04 |
| 5706.334 | 9.24E-05 | 5706.334 | 1.92E-04 | 5706.334 | 4.84E-04 |
| 5707.334 | 2.56E-04 | 5707.334 | 5.65E-05 | 5707.334 | 5.28E-04 |
| 5708.334 | -2.70E-04 | 5708.334 | 4.36E-04 | 5708.334 | 6.98E-05 |
| 5709.334 | -5.09E-05 | 5709.334 | 6.17E-04 | 5709.334 | -1.27E-04 |
| 5710.334 | -1.28E-04 | 5710.334 | 3.74E-04 | 5710.334 | 5.04E-04 |
| 5711.334 | 2.80E-04 | 5711.334 | 9.43E-04 | 5711.334 | 7.63E-04 |
| 5712.334 | 5.14E-04 | 5712.334 | 1.05E-03 | 5712.334 | 3.25E-04 |
| 5713.334 | 8.09E-04 | 5713.334 | 1.30E-03 | 5713.334 | 9.53E-04 |
| 5714.034 | 1.60E-03 | 5714.034 | 2.78E-03 | 5714.034 | 1.88E-03 |
| 5714.133 | 1.81E-03 | 5714.133 | 3.56E-03 | 5714.133 | 1.80E-03 |
| 5714.233 | 1.22E-03 | 5714.233 | 3.26E-03 | 5714.233 | 2.86E-03 |
| 5714.334 | 1.93E-03 | 5714.334 | 3.96E-03 | 5714.334 | 2.71E-03 |
| 5714.433 | 2.07E-03 | 5714.433 | 4.61E-03 | 5714.433 | 3.14E-03 |
| 5714.533 | 2.75E-03 | 5714.533 | 4.83E-03 | 5714.533 | 3.88E-03 |
| 5714.634 | 4.42E-03 | 5714.634 | 6.11E-03 | 5714.634 | 3.38E-03 |
| 5714.733 | 4.34E-03 | 5714.733 | 7.95E-03 | 5714.733 | 4.16E-03 |
| 5714.833 | 5.80E-03 | 5714.833 | 8.96E-03 | 5714.833 | 6.35E-03 |
| 5714.934 | 7.34E-03 | 5714.934 | 1.09E-02 | 5714.934 | 7.18E-03 |
| 5715.034 | 7.59E-03 | 5715.034 | 1.40E-02 | 5715.034 | 8.11E-03 |
| 5715.133 | 8.33E-03 | 5715.133 | 1.83E-02 | 5715.133 | 1.04E-02 |
| 5715.233 | 1.06E-02 | 5715.233 | 2.00E-02 | 5715.233 | 1.27E-02 |
| 5715.334 | 1.26E-02 | 5715.334 | 2.19E-02 | 5715.334 | 1.48E-02 |
| 5715.433 | 1.36E-02 | 5715.433 | 2.58E-02 | 5715.433 | 1.77E-02 |
| 5715.534 | 1.72E-02 | 5715.534 | 3.02E-02 | 5715.534 | 2.17E-02 |
| 5715.634 | 2.10E-02 | 5715.634 | 3.33E-02 | 5715.634 | 2.47E-02 |
| 5715.733 | 2.39E-02 | 5715.733 | 3.59E-02 | 5715.733 | 2.63E-02 |

| | | | | | |
|---|---|---|---|---|---|
| 5715.834 | 2.72E-02 | 5715.834 | 3.76E-02 | 5715.834 | 2.92E-02 |
| 5715.934 | 3.14E-02 | 5715.934 | 4.09E-02 | 5715.934 | 3.20E-02 |
| 5716.033 | 3.40E-02 | 5716.033 | 4.48E-02 | 5716.033 | 3.45E-02 |
| 5716.134 | 3.31E-02 | 5716.134 | 4.40E-02 | 5716.134 | 3.96E-02 |
| 5716.234 | 3.70E-02 | 5716.234 | 4.91E-02 | 5716.234 | 4.17E-02 |
| 5716.333 | 4.41E-02 | 5716.333 | 5.78E-02 | 5716.333 | 4.08E-02 |
| 5716.433 | 5.28E-02 | 5716.433 | 6.61E-02 | 5716.433 | 4.25E-02 |
| 5716.533 | 6.13E-02 | 5716.533 | 7.51E-02 | 5716.533 | 4.72E-02 |
| 5716.634 | 7.00E-02 | 5716.634 | 8.52E-02 | 5716.634 | 5.31E-02 |
| 5716.733 | 8.29E-02 | 5716.733 | 9.60E-02 | 5716.733 | 5.72E-02 |
| 5716.833 | 9.67E-02 | 5716.833 | 0.107778 | 5716.833 | 6.37E-02 |
| 5716.934 | 0.108368 | 5716.934 | 0.119479 | 5716.934 | 7.03E-02 |
| 5717.034 | 0.120095 | 5717.034 | 0.12876 | 5717.034 | 8.03E-02 |
| 5717.134 | 0.128785 | 5717.134 | 0.137812 | 5717.134 | 8.85E-02 |
| 5717.233 | 0.13461 | 5717.233 | 0.145903 | 5717.233 | 9.74E-02 |
| 5717.333 | 0.141905 | 5717.333 | 0.152286 | 5717.333 | 0.102625 |
| 5717.433 | 0.156514 | 5717.433 | 0.155881 | 5717.433 | 0.111304 |
| 5717.533 | 0.159399 | 5717.533 | 0.155066 | 5717.533 | 0.122477 |
| 5717.634 | 0.154851 | 5717.634 | 0.159219 | 5717.634 | 0.127972 |
| 5717.734 | 0.157986 | 5717.734 | 0.161042 | 5717.734 | 0.134119 |
| 5717.834 | 0.166178 | 5717.834 | 0.157046 | 5717.834 | 0.140786 |
| 5717.934 | 0.156232 | 5717.934 | 0.153597 | 5717.934 | 0.144714 |
| 5718.033 | 0.152114 | 5718.033 | 0.153055 | 5718.033 | 0.14298 |
| 5718.133 | 0.148163 | 5718.133 | 0.150053 | 5718.133 | 0.141736 |
| 5718.233 | 0.144819 | 5718.233 | 0.141973 | 5718.233 | 0.141992 |
| 5718.334 | 0.144391 | 5718.334 | 0.141097 | 5718.334 | 0.1405 |
| 5718.434 | 0.137264 | 5718.434 | 0.136399 | 5718.434 | 0.140159 |
| 5718.534 | 0.130061 | 5718.534 | 0.131639 | 5718.534 | 0.136651 |
| 5718.634 | 0.120607 | 5718.634 | 0.129994 | 5718.634 | 0.131473 |
| 5718.733 | 0.116515 | 5718.733 | 0.128011 | 5718.733 | 0.125851 |
| 5718.834 | 0.114146 | 5718.834 | 0.127658 | 5718.834 | 0.127907 |
| 5718.934 | 0.117086 | 5718.934 | 0.126237 | 5718.934 | 0.123132 |
| 5719.034 | 0.111081 | 5719.034 | 0.127926 | 5719.034 | 0.117387 |
| 5719.134 | 0.104731 | 5719.134 | 0.128664 | 5719.134 | 0.116068 |
| 5719.233 | 0.106023 | 5719.233 | 0.131529 | 5719.233 | 0.117756 |
| 5719.333 | 0.111178 | 5719.333 | 0.139107 | 5719.333 | 0.118545 |
| 5719.434 | 0.114953 | 5719.434 | 0.154284 | 5719.434 | 0.120143 |
| 5719.533 | 0.115493 | 5719.533 | 0.163552 | 5719.533 | 0.127167 |
| 5719.634 | 0.118523 | 5719.634 | 0.177822 | 5719.634 | 0.136114 |
| 5719.733 | 0.129417 | 5719.733 | 0.194206 | 5719.733 | 0.142308 |
| 5719.834 | 0.145966 | 5719.834 | 0.213406 | 5719.834 | 0.153574 |
| 5719.934 | 0.156866 | 5719.934 | 0.235874 | 5719.934 | 0.169254 |
| 5720.034 | 0.160032 | 5720.034 | 0.262282 | 5720.034 | 0.188961 |

| | | | | | |
|---|---|---|---|---|---|
| 5720.134 | 0.175336 | 5720.134 | 0.296939 | 5720.134 | 0.210657 |
| 5720.234 | 0.196547 | 5720.234 | 0.325982 | 5720.234 | 0.231941 |
| 5720.333 | 0.208548 | 5720.333 | 0.357288 | 5720.333 | 0.258358 |
| 5720.433 | 0.233204 | 5720.433 | 0.394848 | 5720.433 | 0.284193 |
| 5720.533 | 0.258822 | 5720.533 | 0.434584 | 5720.533 | 0.319587 |
| 5720.633 | 0.286159 | 5720.633 | 0.46832 | 5720.633 | 0.357096 |
| 5720.733 | 0.319836 | 5720.733 | 0.507721 | 5720.733 | 0.396417 |
| 5720.833 | 0.344068 | 5720.833 | 0.553486 | 5720.833 | 0.438248 |
| 5720.934 | 0.374704 | 5720.934 | 0.59381 | 5720.934 | 0.476327 |
| 5721.034 | 0.407869 | 5721.034 | 0.630541 | 5721.034 | 0.513676 |
| 5721.134 | 0.441976 | 5721.134 | 0.671175 | 5721.134 | 0.548742 |
| 5721.233 | 0.481135 | 5721.233 | 0.711539 | 5721.233 | 0.596123 |
| 5721.333 | 0.535395 | 5721.333 | 0.766212 | 5721.333 | 0.647719 |
| 5721.433 | 0.580569 | 5721.433 | 0.824686 | 5721.433 | 0.694213 |
| 5721.533 | 0.630901 | 5721.533 | 0.881849 | 5721.533 | 0.736046 |
| 5721.634 | 0.688201 | 5721.634 | 0.948312 | 5721.634 | 0.787369 |
| 5721.733 | 0.748866 | 5721.733 | 1.029386 | 5721.733 | 0.834201 |
| 5721.833 | 0.833006 | 5721.833 | 1.106854 | 5721.833 | 0.897925 |
| 5721.933 | 0.907739 | 5721.933 | 1.190985 | 5721.933 | 0.957135 |
| 5722.033 | 0.993292 | 5722.033 | 1.269061 | 5722.033 | 1.024792 |
| 5722.134 | 1.081441 | 5722.134 | 1.346184 | 5722.134 | 1.091829 |
| 5722.233 | 1.152763 | 5722.233 | 1.430021 | 5722.233 | 1.159879 |
| 5722.333 | 1.244423 | 5722.333 | 1.501577 | 5722.333 | 1.236863 |
| 5722.434 | 1.319386 | 5722.434 | 1.580738 | 5722.434 | 1.307266 |
| 5722.533 | 1.396087 | 5722.533 | 1.661953 | 5722.533 | 1.388379 |
| 5722.633 | 1.480141 | 5722.633 | 1.725034 | 5722.633 | 1.471015 |
| 5722.733 | 1.552856 | 5722.733 | 1.779091 | 5722.733 | 1.549178 |
| 5722.834 | 1.605394 | 5722.834 | 1.846757 | 5722.834 | 1.645883 |
| 5722.934 | 1.665249 | 5722.934 | 1.91026 | 5722.934 | 1.731344 |
| 5723.033 | 1.750114 | 5723.033 | 1.963505 | 5723.033 | 1.797097 |
| 5723.133 | 1.818717 | 5723.133 | 2.019095 | 5723.133 | 1.870279 |
| 5723.234 | 1.857579 | 5723.234 | 2.064177 | 5723.234 | 1.949304 |
| 5723.333 | 1.948372 | 5723.333 | 2.104659 | 5723.333 | 2.008783 |
| 5723.433 | 2.003052 | 5723.433 | 2.135865 | 5723.433 | 2.080534 |
| 5723.534 | 2.045397 | 5723.534 | 2.196232 | 5723.534 | 2.13867 |
| 5723.633 | 2.099893 | 5723.633 | 2.240903 | 5723.633 | 2.186842 |
| 5723.733 | 2.153872 | 5723.733 | 2.271075 | 5723.733 | 2.240596 |
| 5723.834 | 2.206115 | 5723.834 | 2.290284 | 5723.834 | 2.294358 |
| 5723.933 | 2.224132 | 5723.933 | 2.318048 | 5723.933 | 2.333565 |
| 5724.033 | 2.225865 | 5724.033 | 2.33429 | 5724.033 | 2.358129 |
| 5724.133 | 2.275543 | 5724.133 | 2.352603 | 5724.133 | 2.399118 |
| 5724.234 | 2.314314 | 5724.234 | 2.336913 | 5724.234 | 2.44887 |
| 5724.333 | 2.323911 | 5724.333 | 2.343621 | 5724.333 | 2.456654 |

| | | | | | |
|---|---|---|---|---|---|
| 5724.433 | 2.327334 | 5724.433 | 2.346381 | 5724.433 | 2.460103 |
| 5724.534 | 2.324753 | 5724.534 | 2.333947 | 5724.534 | 2.450459 |
| 5724.633 | 2.314721 | 5724.633 | 2.324311 | 5724.633 | 2.479341 |
| 5724.733 | 2.300366 | 5724.733 | 2.317134 | 5724.733 | 2.480631 |
| 5724.833 | 2.299444 | 5724.833 | 2.307406 | 5724.833 | 2.478676 |
| 5724.934 | 2.290503 | 5724.934 | 2.288078 | 5724.934 | 2.454802 |
| 5725.033 | 2.267578 | 5725.033 | 2.266402 | 5725.033 | 2.452832 |
| 5725.133 | 2.239794 | 5725.133 | 2.244537 | 5725.133 | 2.449239 |
| 5725.233 | 2.223054 | 5725.233 | 2.239268 | 5725.233 | 2.431843 |
| 5725.334 | 2.215745 | 5725.334 | 2.194377 | 5725.334 | 2.411477 |
| 5725.433 | 2.202411 | 5725.433 | 2.165802 | 5725.433 | 2.386608 |
| 5725.533 | 2.125912 | 5725.533 | 2.149791 | 5725.533 | 2.381602 |
| 5725.633 | 2.09873 | 5725.633 | 2.126174 | 5725.633 | 2.345603 |
| 5725.734 | 2.095603 | 5725.734 | 2.110465 | 5725.734 | 2.29795 |
| 5725.833 | 2.050547 | 5725.833 | 2.091822 | 5725.833 | 2.280163 |
| 5725.933 | 2.048507 | 5725.933 | 2.046298 | 5725.933 | 2.243554 |
| 5726.033 | 2.030815 | 5726.033 | 2.005855 | 5726.033 | 2.226494 |
| 5726.133 | 2.001097 | 5726.133 | 1.995419 | 5726.133 | 2.214674 |
| 5726.234 | 1.995692 | 5726.234 | 1.982798 | 5726.234 | 2.186836 |
| 5726.333 | 1.960745 | 5726.333 | 1.948379 | 5726.333 | 2.137725 |
| 5726.433 | 1.906068 | 5726.433 | 1.926515 | 5726.433 | 2.104957 |
| 5726.533 | 1.891904 | 5726.533 | 1.912414 | 5726.533 | 2.058111 |
| 5726.633 | 1.891893 | 5726.633 | 1.883567 | 5726.633 | 2.047446 |
| 5726.734 | 1.855933 | 5726.734 | 1.871516 | 5726.734 | 2.042388 |
| 5726.833 | 1.831758 | 5726.833 | 1.847002 | 5726.833 | 2.010362 |
| 5726.933 | 1.84388 | 5726.933 | 1.824339 | 5726.933 | 1.97545 |
| 5727.033 | 1.83605 | 5727.033 | 1.806473 | 5727.033 | 1.939202 |
| 5727.133 | 1.806746 | 5727.133 | 1.800072 | 5727.133 | 1.918869 |
| 5727.234 | 1.788581 | 5727.234 | 1.792582 | 5727.234 | 1.896302 |
| 5727.334 | 1.781501 | 5727.334 | 1.775828 | 5727.334 | 1.874281 |
| 5727.433 | 1.755052 | 5727.433 | 1.74806 | 5727.433 | 1.86733 |
| 5727.533 | 1.742232 | 5727.533 | 1.74382 | 5727.533 | 1.847525 |
| 5727.633 | 1.744148 | 5727.633 | 1.724643 | 5727.633 | 1.822693 |
| 5727.733 | 1.751835 | 5727.733 | 1.701196 | 5727.733 | 1.810089 |
| 5727.833 | 1.748073 | 5727.833 | 1.71253 | 5727.833 | 1.795985 |
| 5727.934 | 1.735921 | 5727.934 | 1.721567 | 5727.934 | 1.786247 |
| 5728.034 | 1.716506 | 5728.034 | 1.698029 | 5728.034 | 1.761349 |
| 5728.133 | 1.716976 | 5728.133 | 1.680949 | 5728.133 | 1.739604 |
| 5728.233 | 1.684712 | 5728.233 | 1.668899 | 5728.233 | 1.732636 |
| 5728.333 | 1.681855 | 5728.333 | 1.661182 | 5728.333 | 1.718161 |
| 5728.433 | 1.690009 | 5728.433 | 1.667081 | 5728.433 | 1.689084 |
| 5728.533 | 1.711446 | 5728.533 | 1.645872 | 5728.533 | 1.697637 |
| 5728.633 | 1.689858 | 5728.633 | 1.628788 | 5728.633 | 1.697613 |

| | | | | | |
|---|---|---|---|---|---|
| 5728.734 | 1.676063 | 5728.734 | 1.623442 | 5728.734 | 1.686557 |
| 5728.834 | 1.648042 | 5728.834 | 1.616061 | 5728.834 | 1.668642 |
| 5728.934 | 1.630141 | 5728.934 | 1.610178 | 5728.934 | 1.664796 |
| 5729.033 | 1.642995 | 5729.033 | 1.596594 | 5729.033 | 1.651273 |
| 5729.133 | 1.642598 | 5729.133 | 1.587365 | 5729.133 | 1.654898 |
| 5729.233 | 1.630145 | 5729.233 | 1.577438 | 5729.233 | 1.634233 |
| 5729.333 | 1.61782 | 5729.333 | 1.555628 | 5729.333 | 1.614096 |
| 5729.433 | 1.623396 | 5729.433 | 1.551729 | 5729.433 | 1.618086 |
| 5729.533 | 1.629685 | 5729.533 | 1.545467 | 5729.533 | 1.608477 |
| 5729.633 | 1.603552 | 5729.633 | 1.546701 | 5729.633 | 1.606992 |
| 5729.733 | 1.584949 | 5729.733 | 1.543404 | 5729.733 | 1.60248 |
| 5729.833 | 1.572405 | 5729.833 | 1.535904 | 5729.833 | 1.599422 |
| 5729.933 | 1.57209 | 5729.933 | 1.524863 | 5729.933 | 1.584558 |
| 5730.033 | 1.554791 | 5730.033 | 1.522305 | 5730.033 | 1.565477 |
| 5730.134 | 1.567582 | 5730.134 | 1.51468 | 5730.134 | 1.564008 |
| 5730.234 | 1.558673 | 5730.234 | 1.499821 | 5730.234 | 1.567857 |
| 5730.334 | 1.539505 | 5730.334 | 1.49763 | 5730.334 | 1.554084 |
| 5730.434 | 1.529545 | 5730.434 | 1.495195 | 5730.434 | 1.523958 |
| 5730.534 | 1.537564 | 5730.534 | 1.484019 | 5730.534 | 1.52494 |
| 5730.634 | 1.512084 | 5730.634 | 1.469692 | 5730.634 | 1.52127 |
| 5730.734 | 1.493798 | 5730.734 | 1.46746 | 5730.734 | 1.505928 |
| 5730.834 | 1.489896 | 5730.834 | 1.459697 | 5730.834 | 1.509345 |
| 5730.934 | 1.507663 | 5730.934 | 1.450102 | 5730.934 | 1.503567 |
| 5731.067 | 1.485729 | 5731.067 | 1.443281 | 5731.067 | 1.504822 |
| 5731.267 | 1.464088 | 5731.267 | 1.437407 | 5731.267 | 1.498632 |
| 5731.467 | 1.463381 | 5731.467 | 1.424542 | 5731.467 | 1.469509 |
| 5731.667 | 1.447526 | 5731.667 | 1.415361 | 5731.667 | 1.450015 |
| 5731.867 | 1.460096 | 5731.867 | 1.405655 | 5731.867 | 1.451316 |
| 5732.067 | 1.444843 | 5732.067 | 1.393199 | 5732.067 | 1.449852 |
| 5732.266 | 1.425941 | 5732.266 | 1.396249 | 5732.266 | 1.446159 |
| 5732.466 | 1.420983 | 5732.466 | 1.401864 | 5732.466 | 1.445888 |
| 5732.667 | 1.437828 | 5732.667 | 1.393147 | 5732.667 | 1.420615 |
| 5732.867 | 1.407201 | 5732.867 | 1.37969 | 5732.867 | 1.413132 |
| 5733.067 | 1.390714 | 5733.067 | 1.380908 | 5733.067 | 1.416696 |
| 5733.267 | 1.404243 | 5733.267 | 1.393268 | 5733.267 | 1.417195 |
| 5733.467 | 1.433662 | 5733.467 | 1.388628 | 5733.467 | 1.41216 |
| 5733.667 | 1.422591 | 5733.667 | 1.393094 | 5733.667 | 1.401748 |
| 5733.867 | 1.435746 | 5733.867 | 1.404324 | 5733.867 | 1.403631 |
| 5734.067 | 1.431296 | 5734.067 | 1.399113 | 5734.067 | 1.403843 |
| 5734.267 | 1.44495 | 5734.267 | 1.414549 | 5734.267 | 1.41274 |
| 5734.466 | 1.439966 | 5734.466 | 1.427857 | 5734.466 | 1.416946 |
| 5734.667 | 1.469445 | 5734.667 | 1.436223 | 5734.667 | 1.420339 |
| 5734.867 | 1.481291 | 5734.867 | 1.456862 | 5734.867 | 1.434379 |

| | | | | | |
|---|---|---|---|---|---|
| 5735.066 | 1.504403 | 5735.066 | 1.481628 | 5735.066 | 1.439404 |
| 5735.267 | 1.514012 | 5735.267 | 1.49707 | 5735.267 | 1.451353 |
| 5735.467 | 1.53427 | 5735.467 | 1.527198 | 5735.467 | 1.461496 |
| 5735.667 | 1.573051 | 5735.667 | 1.542132 | 5735.667 | 1.477328 |
| 5735.866 | 1.609733 | 5735.866 | 1.577531 | 5735.866 | 1.489504 |
| 5736.066 | 1.635532 | 5736.066 | 1.597861 | 5736.066 | 1.509464 |
| 5736.267 | 1.665668 | 5736.267 | 1.633678 | 5736.267 | 1.518246 |
| 5736.467 | 1.674368 | 5736.467 | 1.651989 | 5736.467 | 1.541563 |
| 5736.667 | 1.673519 | 5736.667 | 1.650554 | 5736.667 | 1.561175 |
| 5736.867 | 1.687158 | 5736.867 | 1.644976 | 5736.867 | 1.574723 |
| 5737.067 | 1.656801 | 5737.067 | 1.648333 | 5737.067 | 1.581612 |
| 5737.267 | 1.631543 | 5737.267 | 1.630172 | 5737.267 | 1.565953 |
| 5737.467 | 1.640175 | 5737.467 | 1.607476 | 5737.467 | 1.558047 |
| 5737.667 | 1.579039 | 5737.667 | 1.572711 | 5737.667 | 1.544362 |
| 5737.866 | 1.544648 | 5737.866 | 1.532678 | 5737.866 | 1.51289 |
| 5738.066 | 1.511147 | 5738.066 | 1.501799 | 5738.066 | 1.474336 |
| 5738.267 | 1.455046 | 5738.267 | 1.465963 | 5738.267 | 1.450393 |
| 5738.467 | 1.410767 | 5738.467 | 1.414895 | 5738.467 | 1.419946 |
| 5738.667 | 1.360701 | 5738.667 | 1.360159 | 5738.667 | 1.379286 |
| 5738.866 | 1.308827 | 5738.866 | 1.311669 | 5738.866 | 1.328974 |
| 5739.066 | 1.278142 | 5739.066 | 1.27099 | 5739.066 | 1.285321 |
| 5739.267 | 1.227225 | 5739.267 | 1.239504 | 5739.267 | 1.243732 |
| 5739.467 | 1.189092 | 5739.467 | 1.196153 | 5739.467 | 1.220922 |
| 5739.667 | 1.146883 | 5739.667 | 1.156736 | 5739.667 | 1.17903 |
| 5739.866 | 1.1055 | 5739.866 | 1.12356 | 5739.866 | 1.154912 |
| 5740.067 | 1.081079 | 5740.067 | 1.094273 | 5740.067 | 1.120674 |
| 5740.267 | 1.078528 | 5740.267 | 1.072423 | 5740.267 | 1.092283 |
| 5740.466 | 1.062976 | 5740.466 | 1.074734 | 5740.466 | 1.064816 |
| 5740.666 | 1.050826 | 5740.666 | 1.054197 | 5740.666 | 1.04931 |
| 5740.866 | 1.029386 | 5740.866 | 1.041992 | 5740.866 | 1.043556 |
| 5741.067 | 1.027976 | 5741.067 | 1.023663 | 5741.067 | 1.016264 |
| 5741.267 | 1.009684 | 5741.267 | 1.030843 | 5741.267 | 1.008097 |
| 5741.467 | 0.98014 | 5741.467 | 1.025581 | 5741.467 | 1.002799 |
| 5741.667 | 0.992159 | 5741.667 | 1.010304 | 5741.667 | 0.993497 |
| 5741.867 | 0.985707 | 5741.867 | 1.01648 | 5741.867 | 0.986546 |
| 5742.066 | 0.979977 | 5742.066 | 1.012296 | 5742.066 | 0.982667 |
| 5742.266 | 0.999912 | 5742.266 | 1.015224 | 5742.266 | 0.981442 |
| 5742.466 | 0.992392 | 5742.466 | 1.015821 | 5742.466 | 0.982353 |
| 5742.666 | 0.993816 | 5742.666 | 1.008703 | 5742.666 | 0.976056 |
| 5742.866 | 1.005895 | 5742.866 | 1.008584 | 5742.866 | 0.976337 |
| 5743.666 | 0.998657 | 5743.666 | 1.013293 | 5743.666 | 0.974343 |
| 5745.667 | 0.972681 | 5745.667 | 1.009797 | 5745.667 | 0.959318 |
| 5747.667 | 0.979779 | 5747.667 | 1.009851 | 5747.667 | 0.964442 |

| | | | | | |
|---|---|---|---|---|---|
| 5749.033 | 0.95347 | 5749.033 | 1.020622 | 5749.033 | 0.975735 |
| 5749.134 | 0.955658 | 5749.134 | 1.017867 | 5749.134 | 0.967079 |
| 5749.233 | 0.96586 | 5749.233 | 1.013228 | 5749.233 | 0.97841 |
| 5749.333 | 0.976284 | 5749.333 | 1.010499 | 5749.333 | 0.975577 |
| 5749.433 | 0.97737 | 5749.433 | 1.016418 | 5749.433 | 0.972456 |
| 5749.534 | 0.972746 | 5749.534 | 1.014353 | 5749.534 | 0.96726 |
| 5749.634 | 0.974048 | 5749.634 | 1.016743 | 5749.634 | 0.980481 |
| 5749.733 | 0.951713 | 5749.733 | 1.020972 | 5749.733 | 0.990051 |
| 5749.833 | 0.967322 | 5749.833 | 1.027143 | 5749.833 | 0.980776 |
| 5749.933 | 0.969594 | 5749.933 | 1.026298 | 5749.933 | 0.966185 |
| 5750.034 | 0.95525 | 5750.034 | 1.023665 | 5750.034 | 0.976012 |
| 5750.134 | 0.960027 | 5750.134 | 1.029473 | 5750.134 | 0.97056 |
| 5750.233 | 0.967867 | 5750.233 | 1.035913 | 5750.233 | 0.96649 |
| 5750.333 | 0.973327 | 5750.333 | 1.039532 | 5750.333 | 0.979514 |
| 5750.433 | 0.989109 | 5750.433 | 1.018296 | 5750.433 | 0.981187 |
| 5750.534 | 0.983708 | 5750.534 | 1.024145 | 5750.534 | 0.965611 |
| 5750.634 | 0.974674 | 5750.634 | 1.030404 | 5750.634 | 0.978098 |
| 5750.733 | 0.969174 | 5750.733 | 1.031185 | 5750.733 | 0.993613 |
| 5750.833 | 0.956196 | 5750.833 | 1.034756 | 5750.833 | 0.99675 |
| 5750.933 | 0.967855 | 5750.933 | 1.031869 | 5750.933 | 0.999647 |
| 5751.033 | 0.985064 | 5751.033 | 1.028481 | 5751.033 | 0.988473 |
| 5751.133 | 0.976287 | 5751.133 | 1.022547 | 5751.133 | 0.985516 |
| 5751.234 | 0.986078 | 5751.234 | 1.023432 | 5751.234 | 0.99199 |
| 5751.333 | 0.988615 | 5751.333 | 1.033846 | 5751.333 | 0.993036 |
| 5751.433 | 0.980908 | 5751.433 | 1.033522 | 5751.433 | 0.989596 |
| 5751.533 | 0.980654 | 5751.533 | 1.037108 | 5751.533 | 0.995647 |
| 5751.633 | 0.990503 | 5751.633 | 1.037753 | 5751.633 | 0.993576 |
| 5751.733 | 0.983098 | 5751.733 | 1.042436 | 5751.733 | 0.99288 |
| 5751.833 | 0.973904 | 5751.833 | 1.03854 | 5751.833 | 0.999074 |
| 5751.934 | 0.991081 | 5751.934 | 1.037277 | 5751.934 | 0.99296 |
| 5752.034 | 0.99875 | 5752.034 | 1.04551 | 5752.034 | 0.997341 |
| 5752.133 | 0.987277 | 5752.133 | 1.048347 | 5752.133 | 0.996292 |
| 5752.233 | 0.970644 | 5752.233 | 1.040505 | 5752.233 | 0.990164 |
| 5752.333 | 0.988785 | 5752.333 | 1.046124 | 5752.966 | 1.007254 |
| 5752.433 | 0.976106 | 5752.433 | 1.050533 | 5754.966 | 1.022043 |
| 5752.533 | 0.971318 | 5752.533 | 1.051267 | 5756.967 | 1.042348 |
| 5752.633 | 0.987521 | 5752.633 | 1.048567 | 5758.967 | 1.058243 |
| 5752.733 | 1.001996 | 5752.733 | 1.053199 | 5761.966 | 1.066126 |
| 5752.833 | 0.990001 | 5752.833 | 1.043525 | 5766.966 | 1.063166 |
| 5752.934 | 0.996856 | 5752.934 | 1.035409 | 5771.967 | 1.074787 |
| 5753.034 | 0.999537 | 5753.034 | 1.039785 | 5776.967 | 1.062527 |
| 5753.134 | 0.982594 | 5753.134 | 1.042761 | 5781.966 | 1.059871 |
| 5753.234 | 0.975726 | 5753.234 | 1.043046 | 5786.967 | 1.0613 |

| | | | | | |
|---|---|---|---|---|---|
| 5753.333 | 0.973403 | 5753.333 | 1.04104 | 5791.967 | 1.071316 |
| 5753.433 | 0.99177 | 5753.433 | 1.047252 | 5796.966 | 1.069947 |
| 5753.533 | 0.997624 | 5753.533 | 1.042965 | 5803.633 | 1.069888 |
| 5754.267 | 1.008175 | 5754.267 | 1.041007 | 5813.633 | 1.101077 |
| 5756.267 | 1.017792 | 5756.267 | 1.06792 | 5823.633 | 1.115974 |
| 5758.267 | 1.023726 | 5758.267 | 1.079991 | 5833.633 | 1.11957 |
| 5760.266 | 1.027288 | 5760.266 | 1.088906 | | |
| 5763.267 | 1.058492 | 5763.267 | 1.098195 | | |
| 5768.267 | 1.076425 | 5768.267 | 1.107816 | | |
| 5773.267 | 1.090557 | 5773.267 | 1.11954 | | |
| 5778.267 | 1.070867 | 5778.267 | 1.106419 | | |
| 5783.267 | 1.052777 | 5783.267 | 1.099183 | | |
| 5788.267 | 1.072286 | 5788.267 | 1.093991 | | |
| 5793.266 | 1.070971 | 5793.266 | 1.10395 | | |
| 5798.267 | 1.077907 | 5798.267 | 1.109809 | | |
| 5804.934 | 1.082613 | 5804.934 | 1.121856 | | |
| 5814.933 | 1.110298 | 5814.933 | 1.132134 | | |
| 5824.933 | 1.128978 | 5824.933 | 1.143113 | | |
| 5834.934 | 1.134497 | 5834.934 | 1.168358 | | |
| 5844.933 | 1.155933 | 5844.933 | 1.185251 | | |
| 5854.933 | 1.163757 | 5854.933 | 1.195473 | | |
| 5864.934 | 1.152966 | 5864.934 | 1.192205 | | |
| 5874.933 | 1.158671 | 5874.933 | 1.199073 | | |
| 5884.933 | 1.165858 | 5884.933 | 1.217158 | | |

**Table S9.** Oxo HERFD

| **[2-K]$_4$** | | **[2-Rb]$_4$** | | **[2-Cs]$_4$** | |
|---|---|---|---|---|---|
| E(eV) | Normalized HERFD | E(eV) | Normalized HERFD | E(eV) | Normalized HERFD |
| 5680 | -7.83E-05 | 5680 | 1.69E-04 | 5680 | 4.17E-04 |
| 5681.334 | 8.2E-05 | 5681.334 | 2.07E-04 | 5681.334 | -4.62E-04 |
| 5682.333 | 0.00027 | 5682.333 | 4.65E-05 | 5682.333 | -5.80E-05 |
| 5683.333 | -5.61E-05 | 5683.333 | -1.78E-04 | 5683.333 | -1.99E-04 |
| 5684.334 | -9.66E-05 | 5684.334 | -1.73E-04 | 5684.334 | -8.78E-05 |
| 5685.334 | -2.05E-05 | 5685.334 | -1.50E-05 | 5685.334 | -7.14E-06 |
| 5686.333 | 0.000117 | 5686.333 | -5.06E-05 | 5686.333 | -1.12E-04 |
| 5687.333 | -6.04E-05 | 5687.333 | -2.83E-05 | 5687.333 | 1.13E-05 |
| 5688.334 | -5.94E-05 | 5688.334 | -8.87E-06 | 5688.334 | -2.40E-04 |
| 5689.334 | -8.14E-05 | 5689.334 | -2.94E-04 | 5689.334 | -1.73E-05 |
| 5690.334 | 0.000147 | 5690.334 | -5.22E-05 | 5690.334 | -3.25E-04 |
| 5691.333 | 5.85E-05 | 5691.333 | 5.37E-05 | 5691.333 | 1.76E-04 |
| 5692.333 | -3.87E-05 | 5692.333 | -9.36E-06 | 5692.333 | 4.29E-04 |

| | | | | | |
|---|---|---|---|---|---|
| 5693.333 | -7.02E-05 | 5693.333 | -8.45E-05 | 5693.333 | 1.06E-04 |
| 5694.333 | 6.34E-05 | 5694.333 | 4.35E-05 | 5694.333 | 1.12E-04 |
| 5695.334 | 0.000117 | 5695.334 | 1.09E-04 | 5695.334 | 1.91E-05 |
| 5696.334 | -3.72E-05 | 5696.334 | 1.79E-05 | 5696.334 | 3.64E-04 |
| 5697.333 | -4.08E-05 | 5697.333 | -8.17E-05 | 5697.333 | 9.07E-05 |
| 5698.334 | -0.000115 | 5698.334 | 1.90E-04 | 5698.334 | 5.09E-05 |
| 5699.334 | -0.000124 | 5699.334 | 1.80E-04 | 5699.334 | 1.19E-04 |
| 5700.334 | 3.94E-05 | 5700.334 | 1.46E-04 | 5700.334 | 2.50E-04 |
| 5701.334 | -4.94E-05 | 5701.334 | -2.08E-05 | 5701.334 | 8.38E-05 |
| 5702.334 | -0.000124 | 5702.334 | 2.40E-06 | 5702.334 | 2.89E-04 |
| 5703.334 | -4.75E-05 | 5703.334 | 2.33E-05 | 5703.334 | -5.45E-05 |
| 5704.334 | -0.000143 | 5704.334 | 1.15E-04 | 5704.334 | 1.18E-04 |
| 5705.334 | -6.64E-05 | 5705.334 | 1.04E-04 | 5705.334 | 3.52E-04 |
| 5706.334 | 4.36E-05 | 5706.334 | 9.21E-05 | 5706.334 | 1.65E-04 |
| 5707.334 | 0.000129 | 5707.334 | -7.76E-05 | 5707.334 | 4.81E-04 |
| 5708.334 | 8.68E-05 | 5708.334 | 8.82E-05 | 5708.334 | -1.03E-05 |
| 5709.334 | 9.63E-05 | 5709.334 | 1.56E-04 | 5709.334 | -6.47E-05 |
| 5710.334 | 0.000418 | 5710.334 | 1.52E-05 | 5710.334 | 5.97E-04 |
| 5711.334 | 0.000389 | 5711.334 | 1.82E-04 | 5711.334 | 5.85E-05 |
| 5712.334 | 0.000411 | 5712.334 | 5.98E-04 | 5712.334 | -4.53E-05 |
| 5713.334 | 0.00068 | 5713.334 | 7.11E-04 | 5713.334 | 8.59E-04 |
| 5714.034 | 0.001263 | 5714.034 | 1.11E-03 | 5714.034 | 1.42E-03 |
| 5714.133 | 0.001452 | 5714.133 | 1.07E-03 | 5714.133 | 1.13E-03 |
| 5714.233 | 0.001637 | 5714.233 | 1.35E-03 | 5714.233 | 1.33E-03 |
| 5714.334 | 0.00192 | 5714.334 | 1.87E-03 | 5714.334 | 1.81E-03 |
| 5714.433 | 0.002271 | 5714.433 | 1.43E-03 | 5714.433 | 1.95E-03 |
| 5714.533 | 0.002598 | 5714.533 | 1.46E-03 | 5714.533 | 1.83E-03 |
| 5714.634 | 0.003436 | 5714.634 | 2.18E-03 | 5714.634 | 2.34E-03 |
| 5714.733 | 0.003919 | 5714.733 | 2.79E-03 | 5714.733 | 4.02E-03 |
| 5714.833 | 0.004586 | 5714.833 | 2.68E-03 | 5714.833 | 4.55E-03 |
| 5714.934 | 0.00558 | 5714.934 | 2.99E-03 | 5714.934 | 5.10E-03 |
| 5715.034 | 0.006769 | 5715.034 | 4.07E-03 | 5715.034 | 5.39E-03 |
| 5715.133 | 0.00784 | 5715.133 | 5.00E-03 | 5715.133 | 5.22E-03 |
| 5715.233 | 0.009122 | 5715.233 | 5.57E-03 | 5715.233 | 6.85E-03 |
| 5715.334 | 0.010713 | 5715.334 | 6.45E-03 | 5715.334 | 8.47E-03 |
| 5715.433 | 0.012554 | 5715.433 | 7.56E-03 | 5715.433 | 1.03E-02 |
| 5715.534 | 0.015022 | 5715.534 | 7.84E-03 | 5715.534 | 1.08E-02 |
| 5715.634 | 0.017422 | 5715.634 | 9.45E-03 | 5715.634 | 1.28E-02 |
| 5715.733 | 0.020967 | 5715.733 | 1.09E-02 | 5715.733 | 1.50E-02 |
| 5715.834 | 0.024137 | 5715.834 | 1.21E-02 | 5715.834 | 1.58E-02 |
| 5715.934 | 0.025903 | 5715.934 | 1.35E-02 | 5715.934 | 1.73E-02 |
| 5716.033 | 0.028552 | 5716.033 | 1.54E-02 | 5716.033 | 2.03E-02 |
| 5716.134 | 0.031863 | 5716.134 | 1.71E-02 | 5716.134 | 2.18E-02 |

| | | | | | |
|---|---|---|---|---|---|
| 5716.234 | 0.034197 | 5716.234 | 1.92E-02 | 5716.234 | 2.45E-02 |
| 5716.333 | 0.036217 | 5716.333 | 2.16E-02 | 5716.333 | 2.34E-02 |
| 5716.433 | 0.038576 | 5716.433 | 2.47E-02 | 5716.433 | 2.73E-02 |
| 5716.533 | 0.040098 | 5716.533 | 2.91E-02 | 5716.533 | 3.09E-02 |
| 5716.634 | 0.040824 | 5716.634 | 3.37E-02 | 5716.634 | 3.14E-02 |
| 5716.733 | 0.043297 | 5716.733 | 3.89E-02 | 5716.733 | 3.66E-02 |
| 5716.833 | 0.045267 | 5716.833 | 4.81E-02 | 5716.833 | 4.48E-02 |
| 5716.934 | 0.050073 | 5716.934 | 5.84E-02 | 5716.934 | 5.12E-02 |
| 5717.034 | 0.055943 | 5717.034 | 7.22E-02 | 5717.034 | 5.95E-02 |
| 5717.134 | 0.062752 | 5717.134 | 8.65E-02 | 5717.134 | 6.82E-02 |
| 5717.233 | 0.073818 | 5717.233 | 0.103055 | 5717.233 | 7.99E-02 |
| 5717.333 | 0.086181 | 5717.333 | 0.121111 | 5717.333 | 9.55E-02 |
| 5717.433 | 0.097419 | 5717.433 | 0.143541 | 5717.433 | 0.114968 |
| 5717.533 | 0.111861 | 5717.533 | 0.166232 | 5717.533 | 0.133718 |
| 5717.634 | 0.126053 | 5717.634 | 0.185237 | 5717.634 | 0.148032 |
| 5717.734 | 0.142265 | 5717.734 | 0.202743 | 5717.734 | 0.160296 |
| 5717.834 | 0.156936 | 5717.834 | 0.222009 | 5717.834 | 0.180097 |
| 5717.934 | 0.172726 | 5717.934 | 0.236061 | 5717.934 | 0.189792 |
| 5718.033 | 0.183451 | 5718.033 | 0.241061 | 5718.033 | 0.193241 |
| 5718.133 | 0.191507 | 5718.133 | 0.240581 | 5718.133 | 0.199034 |
| 5718.233 | 0.19812 | 5718.233 | 0.2365 | 5718.233 | 0.199394 |
| 5718.334 | 0.200467 | 5718.334 | 0.229103 | 5718.334 | 0.194802 |
| 5718.434 | 0.198427 | 5718.434 | 0.21923 | 5718.434 | 0.18957 |
| 5718.534 | 0.192488 | 5718.534 | 0.209686 | 5718.534 | 0.179033 |
| 5718.634 | 0.186766 | 5718.634 | 0.195437 | 5718.634 | 0.167785 |
| 5718.733 | 0.180671 | 5718.733 | 0.181673 | 5718.733 | 0.15642 |
| 5718.834 | 0.170565 | 5718.834 | 0.16795 | 5718.834 | 0.150546 |
| 5718.934 | 0.161981 | 5718.934 | 0.152111 | 5718.934 | 0.144173 |
| 5719.034 | 0.15377 | 5719.034 | 0.142241 | 5719.034 | 0.138684 |
| 5719.134 | 0.144504 | 5719.134 | 0.128026 | 5719.134 | 0.123541 |
| 5719.233 | 0.1355 | 5719.233 | 0.115206 | 5719.233 | 0.109434 |
| 5719.333 | 0.128245 | 5719.333 | 0.107411 | 5719.333 | 0.103502 |
| 5719.434 | 0.120837 | 5719.434 | 0.100365 | 5719.434 | 0.103622 |
| 5719.533 | 0.117586 | 5719.533 | 9.52E-02 | 5719.533 | 9.91E-02 |
| 5719.634 | 0.115187 | 5719.634 | 9.17E-02 | 5719.634 | 9.47E-02 |
| 5719.733 | 0.114228 | 5719.733 | 9.07E-02 | 5719.733 | 9.36E-02 |
| 5719.834 | 0.116804 | 5719.834 | 8.91E-02 | 5719.834 | 9.52E-02 |
| 5719.934 | 0.11941 | 5719.934 | 9.21E-02 | 5719.934 | 9.76E-02 |
| 5720.034 | 0.123304 | 5720.034 | 9.40E-02 | 5720.034 | 9.66E-02 |
| 5720.134 | 0.131886 | 5720.134 | 9.66E-02 | 5720.134 | 0.106728 |
| 5720.234 | 0.142917 | 5720.234 | 0.100595 | 5720.234 | 0.118912 |
| 5720.333 | 0.155441 | 5720.333 | 0.107825 | 5720.333 | 0.126437 |
| 5720.433 | 0.171574 | 5720.433 | 0.115498 | 5720.433 | 0.136015 |

| | | | | | |
|---|---|---|---|---|---|
| 5720.533 | 0.195832 | 5720.533 | 0.127099 | 5720.533 | 0.151017 |
| 5720.633 | 0.219212 | 5720.633 | 0.137458 | 5720.633 | 0.17326 |
| 5720.733 | 0.2455 | 5720.733 | 0.150665 | 5720.733 | 0.196201 |
| 5720.833 | 0.274996 | 5720.833 | 0.165486 | 5720.833 | 0.212259 |
| 5720.934 | 0.305858 | 5720.934 | 0.176476 | 5720.934 | 0.225304 |
| 5721.034 | 0.341592 | 5721.034 | 0.189283 | 5721.034 | 0.248469 |
| 5721.134 | 0.37954 | 5721.134 | 0.208017 | 5721.134 | 0.283561 |
| 5721.233 | 0.418958 | 5721.233 | 0.225417 | 5721.233 | 0.307953 |
| 5721.333 | 0.465406 | 5721.333 | 0.242999 | 5721.333 | 0.324916 |
| 5721.433 | 0.508921 | 5721.433 | 0.259158 | 5721.433 | 0.352148 |
| 5721.533 | 0.549536 | 5721.533 | 0.274171 | 5721.533 | 0.387496 |
| 5721.634 | 0.586687 | 5721.634 | 0.293144 | 5721.634 | 0.415255 |
| 5721.733 | 0.62861 | 5721.733 | 0.319195 | 5721.733 | 0.441759 |
| 5721.833 | 0.667527 | 5721.833 | 0.34326 | 5721.833 | 0.474312 |
| 5721.933 | 0.706408 | 5721.933 | 0.36964 | 5721.933 | 0.50985 |
| 5722.033 | 0.748384 | 5722.033 | 0.404437 | 5722.033 | 0.546996 |
| 5722.134 | 0.789966 | 5722.134 | 0.441219 | 5722.134 | 0.602186 |
| 5722.233 | 0.832312 | 5722.233 | 0.483449 | 5722.233 | 0.657056 |
| 5722.333 | 0.876237 | 5722.333 | 0.534301 | 5722.333 | 0.690199 |
| 5722.434 | 0.92814 | 5722.434 | 0.594553 | 5722.434 | 0.73203 |
| 5722.533 | 0.985593 | 5722.533 | 0.657062 | 5722.533 | 0.802228 |
| 5722.633 | 1.047997 | 5722.633 | 0.731426 | 5722.633 | 0.882735 |
| 5722.733 | 1.115569 | 5722.733 | 0.810629 | 5722.733 | 0.966657 |
| 5722.834 | 1.190181 | 5722.834 | 0.898035 | 5722.834 | 1.062366 |
| 5722.934 | 1.270306 | 5722.934 | 1.000318 | 5722.934 | 1.152057 |
| 5723.033 | 1.351698 | 5723.033 | 1.102507 | 5723.033 | 1.24099 |
| 5723.133 | 1.443081 | 5723.133 | 1.207454 | 5723.133 | 1.350488 |
| 5723.234 | 1.534745 | 5723.234 | 1.3162 | 5723.234 | 1.450643 |
| 5723.333 | 1.625287 | 5723.333 | 1.418477 | 5723.333 | 1.552819 |
| 5723.433 | 1.716839 | 5723.433 | 1.518055 | 5723.433 | 1.652558 |
| 5723.534 | 1.80936 | 5723.534 | 1.618617 | 5723.534 | 1.748161 |
| 5723.633 | 1.898487 | 5723.633 | 1.702422 | 5723.633 | 1.84359 |
| 5723.733 | 1.979726 | 5723.733 | 1.785289 | 5723.733 | 1.945627 |
| 5723.834 | 2.048657 | 5723.834 | 1.854367 | 5723.834 | 2.046075 |
| 5723.933 | 2.121745 | 5723.933 | 1.92756 | 5723.933 | 2.119167 |
| 5724.033 | 2.188074 | 5724.033 | 1.998552 | 5724.033 | 2.188683 |
| 5724.133 | 2.245525 | 5724.133 | 2.048423 | 5724.133 | 2.261099 |
| 5724.234 | 2.286807 | 5724.234 | 2.096754 | 5724.234 | 2.30203 |
| 5724.333 | 2.333307 | 5724.333 | 2.144606 | 5724.333 | 2.325877 |
| 5724.433 | 2.370751 | 5724.433 | 2.172969 | 5724.433 | 2.376807 |
| 5724.534 | 2.400095 | 5724.534 | 2.192943 | 5724.534 | 2.400536 |
| 5724.633 | 2.416801 | 5724.633 | 2.20182 | 5724.633 | 2.418454 |
| 5724.733 | 2.432196 | 5724.733 | 2.209096 | 5724.733 | 2.430029 |

| | | | | | |
|---|---|---|---|---|---|
| 5724.833 | 2.430977 | 5724.833 | 2.196509 | 5724.833 | 2.417825 |
| 5724.934 | 2.425363 | 5724.934 | 2.178859 | 5724.934 | 2.397697 |
| 5725.033 | 2.413702 | 5725.033 | 2.160752 | 5725.033 | 2.361923 |
| 5725.133 | 2.393716 | 5725.133 | 2.137129 | 5725.133 | 2.332598 |
| 5725.233 | 2.374926 | 5725.233 | 2.103657 | 5725.233 | 2.319668 |
| 5725.334 | 2.350233 | 5725.334 | 2.081085 | 5725.334 | 2.300872 |
| 5725.433 | 2.319214 | 5725.433 | 2.060451 | 5725.433 | 2.245042 |
| 5725.533 | 2.274731 | 5725.533 | 2.019226 | 5725.533 | 2.220206 |
| 5725.633 | 2.247448 | 5725.633 | 1.986192 | 5725.633 | 2.210704 |
| 5725.734 | 2.221276 | 5725.734 | 1.959954 | 5725.734 | 2.156557 |
| 5725.833 | 2.184051 | 5725.833 | 1.931308 | 5725.833 | 2.126081 |
| 5725.933 | 2.149735 | 5725.933 | 1.896939 | 5725.933 | 2.088646 |
| 5726.033 | 2.11802 | 5726.033 | 1.85966 | 5726.033 | 2.058235 |
| 5726.133 | 2.085558 | 5726.133 | 1.842043 | 5726.133 | 2.03794 |
| 5726.234 | 2.05387 | 5726.234 | 1.815949 | 5726.234 | 1.98741 |
| 5726.333 | 2.017907 | 5726.333 | 1.788891 | 5726.333 | 1.946084 |
| 5726.433 | 1.982859 | 5726.433 | 1.756806 | 5726.433 | 1.89903 |
| 5726.533 | 1.949397 | 5726.533 | 1.731012 | 5726.533 | 1.891292 |
| 5726.633 | 1.913217 | 5726.633 | 1.710081 | 5726.633 | 1.847943 |
| 5726.734 | 1.874442 | 5726.734 | 1.688309 | 5726.734 | 1.806573 |
| 5726.833 | 1.842247 | 5726.833 | 1.658396 | 5726.833 | 1.792974 |
| 5726.933 | 1.816776 | 5726.933 | 1.64299 | 5726.933 | 1.786141 |
| 5727.033 | 1.788837 | 5727.033 | 1.626353 | 5727.033 | 1.754647 |
| 5727.133 | 1.759233 | 5727.133 | 1.624715 | 5727.133 | 1.735003 |
| 5727.234 | 1.732483 | 5727.234 | 1.610602 | 5727.234 | 1.723793 |
| 5727.334 | 1.7004 | 5727.334 | 1.595836 | 5727.334 | 1.703367 |
| 5727.433 | 1.674785 | 5727.433 | 1.58375 | 5727.433 | 1.693359 |
| 5727.533 | 1.65727 | 5727.533 | 1.57054 | 5727.533 | 1.675556 |
| 5727.633 | 1.635017 | 5727.633 | 1.56576 | 5727.633 | 1.675318 |
| 5727.733 | 1.616595 | 5727.733 | 1.563042 | 5727.733 | 1.660105 |
| 5727.833 | 1.597963 | 5727.833 | 1.54856 | 5727.833 | 1.649879 |
| 5727.934 | 1.579799 | 5727.934 | 1.545948 | 5727.934 | 1.640335 |
| 5728.034 | 1.56558 | 5728.034 | 1.543391 | 5728.034 | 1.646746 |
| 5728.133 | 1.562172 | 5728.133 | 1.541088 | 5728.133 | 1.633756 |
| 5728.233 | 1.549498 | 5728.233 | 1.543629 | 5728.233 | 1.630647 |
| 5728.333 | 1.540896 | 5728.333 | 1.537819 | 5728.333 | 1.637093 |
| 5728.433 | 1.533139 | 5728.433 | 1.542899 | 5728.433 | 1.629638 |
| 5728.533 | 1.52954 | 5728.533 | 1.542199 | 5728.533 | 1.631593 |
| 5728.633 | 1.524595 | 5728.633 | 1.546031 | 5728.633 | 1.623987 |
| 5728.734 | 1.519603 | 5728.734 | 1.538812 | 5728.734 | 1.619239 |
| 5728.834 | 1.512554 | 5728.834 | 1.545859 | 5728.834 | 1.626531 |
| 5728.934 | 1.504049 | 5728.934 | 1.547811 | 5728.934 | 1.612796 |
| 5729.033 | 1.504506 | 5729.033 | 1.53494 | 5729.033 | 1.619416 |

| | | | | | |
|---|---|---|---|---|---|
| 5729.133 | 1.500911 | 5729.133 | 1.540082 | 5729.133 | 1.612609 |
| 5729.233 | 1.501727 | 5729.233 | 1.548273 | 5729.233 | 1.609607 |
| 5729.333 | 1.495069 | 5729.333 | 1.546501 | 5729.333 | 1.619954 |
| 5729.433 | 1.489105 | 5729.433 | 1.539108 | 5729.433 | 1.635474 |
| 5729.533 | 1.482224 | 5729.533 | 1.542818 | 5729.533 | 1.614979 |
| 5729.633 | 1.481069 | 5729.633 | 1.552068 | 5729.633 | 1.623977 |
| 5729.733 | 1.479906 | 5729.733 | 1.542437 | 5729.733 | 1.619951 |
| 5729.833 | 1.480971 | 5729.833 | 1.536641 | 5729.833 | 1.595092 |
| 5729.933 | 1.470507 | 5729.933 | 1.528335 | 5729.933 | 1.599044 |
| 5730.033 | 1.469074 | 5730.033 | 1.527978 | 5730.033 | 1.587026 |
| 5730.134 | 1.467868 | 5730.134 | 1.527768 | 5730.134 | 1.579141 |
| 5730.234 | 1.461425 | 5730.234 | 1.534446 | 5730.234 | 1.592529 |
| 5730.334 | 1.453874 | 5730.334 | 1.530735 | 5730.334 | 1.588406 |
| 5730.434 | 1.453334 | 5730.434 | 1.524089 | 5730.434 | 1.594239 |
| 5730.534 | 1.451069 | 5730.534 | 1.515574 | 5730.534 | 1.584653 |
| 5730.634 | 1.446692 | 5730.634 | 1.512541 | 5730.634 | 1.567023 |
| 5730.734 | 1.439207 | 5730.734 | 1.511266 | 5730.734 | 1.562881 |
| 5730.834 | 1.436619 | 5730.834 | 1.509585 | 5730.834 | 1.537816 |
| 5730.934 | 1.430649 | 5730.934 | 1.50157 | 5730.934 | 1.559899 |
| 5731.134 | 1.425398 | 5731.067 | 1.500399 | 5731.067 | 1.550292 |
| 5731.534 | 1.407369 | 5731.267 | 1.491571 | 5731.267 | 1.54104 |
| 5731.934 | 1.395373 | 5731.467 | 1.480645 | 5731.467 | 1.517188 |
| 5732.333 | 1.378749 | 5731.667 | 1.473128 | 5731.667 | 1.499145 |
| 5732.733 | 1.367115 | 5731.867 | 1.46961 | 5731.867 | 1.493902 |
| 5733.133 | 1.347039 | 5732.067 | 1.456631 | 5732.067 | 1.486257 |
| 5733.534 | 1.334082 | 5732.266 | 1.442168 | 5732.266 | 1.482532 |
| 5733.934 | 1.321657 | 5732.466 | 1.439607 | 5732.466 | 1.480317 |
| 5734.334 | 1.305025 | 5732.667 | 1.430629 | 5732.667 | 1.464633 |
| 5734.733 | 1.297529 | 5732.867 | 1.426263 | 5732.867 | 1.442465 |
| 5735.133 | 1.286058 | 5733.067 | 1.417425 | 5733.067 | 1.447091 |
| 5735.534 | 1.283166 | 5733.267 | 1.410392 | 5733.267 | 1.440277 |
| 5735.933 | 1.297726 | 5733.467 | 1.400252 | 5733.467 | 1.437174 |
| 5736.334 | 1.325724 | 5733.667 | 1.397227 | 5733.667 | 1.423821 |
| 5736.733 | 1.339407 | 5733.867 | 1.386939 | 5733.867 | 1.408215 |
| 5737.133 | 1.359206 | 5734.067 | 1.380466 | 5734.067 | 1.407793 |
| 5737.533 | 1.367234 | 5734.267 | 1.378752 | 5734.267 | 1.414789 |
| 5737.933 | 1.347796 | 5734.466 | 1.377919 | 5734.466 | 1.404868 |
| 5738.334 | 1.304101 | 5734.667 | 1.37429 | 5734.667 | 1.411031 |
| 5738.733 | 1.250329 | 5734.867 | 1.373066 | 5734.867 | 1.415833 |
| 5739.133 | 1.178568 | 5735.066 | 1.379828 | 5735.066 | 1.421776 |
| 5739.533 | 1.112475 | 5735.267 | 1.384459 | 5735.267 | 1.417833 |
| 5739.933 | 1.06525 | 5735.467 | 1.38244 | 5735.467 | 1.409894 |
| 5740.334 | 1.0346 | 5735.667 | 1.397042 | 5735.667 | 1.413177 |

| | | | | | |
|---|---|---|---|---|---|
| 5740.733 | 1.007871 | 5735.866 | 1.416205 | 5735.866 | 1.420711 |
| 5741.134 | 0.986312 | 5736.066 | 1.413517 | 5736.066 | 1.436972 |
| 5741.533 | 0.974753 | 5736.267 | 1.431157 | 5736.267 | 1.461224 |
| 5741.933 | 0.957858 | 5736.467 | 1.45469 | 5736.467 | 1.483896 |
| 5742.333 | 0.946274 | 5736.667 | 1.475554 | 5736.667 | 1.479349 |
| 5742.733 | 0.943366 | 5736.867 | 1.490256 | 5736.867 | 1.479434 |
| 5743.666 | 0.939641 | 5737.067 | 1.493966 | 5737.067 | 1.494189 |
| 5745.667 | 0.936467 | 5737.267 | 1.510407 | 5737.267 | 1.517835 |
| 5747.667 | 0.93507 | 5737.467 | 1.50595 | 5737.467 | 1.504339 |
| 5749.033 | 0.937902 | 5737.667 | 1.484613 | 5737.667 | 1.482489 |
| 5749.134 | 0.935404 | 5737.866 | 1.4672 | 5737.866 | 1.465604 |
| 5749.233 | 0.934597 | 5738.066 | 1.429385 | 5738.066 | 1.419927 |
| 5749.333 | 0.938344 | 5738.267 | 1.392985 | 5738.267 | 1.390756 |
| 5749.433 | 0.940013 | 5738.467 | 1.350078 | 5738.467 | 1.34074 |
| 5749.534 | 0.939788 | 5738.667 | 1.307979 | 5738.667 | 1.286852 |
| 5749.634 | 0.941752 | 5738.866 | 1.25991 | 5738.866 | 1.24805 |
| 5749.733 | 0.944731 | 5739.066 | 1.213923 | 5739.066 | 1.22797 |
| 5749.833 | 0.942052 | 5739.267 | 1.174454 | 5739.267 | 1.190985 |
| 5749.933 | 0.940587 | 5739.467 | 1.150947 | 5739.467 | 1.165888 |
| 5750.034 | 0.939725 | 5739.667 | 1.120378 | 5739.667 | 1.132357 |
| 5750.134 | 0.937073 | 5739.866 | 1.097482 | 5739.866 | 1.111004 |
| 5750.233 | 0.937149 | 5740.067 | 1.075218 | 5740.067 | 1.079705 |
| 5750.333 | 0.938329 | 5740.267 | 1.061728 | 5740.267 | 1.075712 |
| 5750.433 | 0.937867 | 5740.466 | 1.049086 | 5740.466 | 1.058164 |
| 5750.534 | 0.941595 | 5740.666 | 1.03175 | 5740.666 | 1.033454 |
| 5750.634 | 0.943886 | 5740.866 | 1.02024 | 5740.866 | 1.019552 |
| 5750.733 | 0.940718 | 5741.067 | 1.007376 | 5741.067 | 1.017996 |
| 5750.833 | 0.94439 | 5741.267 | 0.996921 | 5741.267 | 1.005848 |
| 5750.933 | 0.946373 | 5741.467 | 0.987924 | 5741.467 | 0.983102 |
| 5751.033 | 0.945647 | 5741.667 | 0.985345 | 5741.667 | 0.987292 |
| 5751.133 | 0.948307 | 5741.867 | 0.979366 | 5741.867 | 0.979743 |
| 5751.234 | 0.949953 | 5742.066 | 0.972216 | 5742.066 | 0.970451 |
| 5751.333 | 0.952756 | 5742.266 | 0.966221 | 5742.266 | 0.959122 |
| 5751.433 | 0.952954 | 5742.466 | 0.961978 | 5742.466 | 0.974811 |
| 5751.533 | 0.956651 | 5742.666 | 0.951126 | 5742.666 | 0.972944 |
| 5751.633 | 0.952517 | 5742.866 | 0.955545 | 5742.866 | 0.948065 |
| 5751.733 | 0.949566 | 5743.666 | 0.953142 | 5743.666 | 0.931168 |
| 5751.833 | 0.949523 | 5745.667 | 0.94842 | 5745.667 | 0.923846 |
| 5751.934 | 0.952572 | 5747.667 | 0.934471 | 5747.667 | 0.921909 |
| 5752.034 | 0.953004 | 5749.033 | 0.931265 | 5749.033 | 0.898014 |
| 5752.133 | 0.951576 | 5749.134 | 0.930478 | 5749.134 | 0.897157 |
| 5752.233 | 0.954346 | 5749.233 | 0.926906 | 5749.233 | 0.907455 |
| 5752.966 | 0.963788 | 5749.333 | 0.924347 | 5749.333 | 0.910895 |

| | | | | | |
|---|---|---|---|---|---|
| 5754.966 | 0.983469 | 5749.433 | 0.925883 | 5749.433 | 0.932974 |
| 5756.967 | 0.999296 | 5749.534 | 0.929799 | 5749.534 | 0.93388 |
| 5758.967 | 1.005198 | 5749.634 | 0.930332 | 5749.634 | 0.911291 |
| 5761.966 | 1.003551 | 5749.733 | 0.93057 | 5749.733 | 0.932646 |
| 5766.966 | 1.003472 | 5749.833 | 0.933242 | 5749.833 | 0.928811 |
| 5771.967 | 1.009996 | 5749.933 | 0.939955 | 5749.933 | 0.915217 |
| 5776.967 | 1.009537 | 5750.034 | 0.933311 | 5750.034 | 0.916296 |
| 5781.966 | 1.007535 | 5750.134 | 0.928351 | 5750.134 | 0.916545 |
| 5786.967 | 1.00499 | 5750.233 | 0.926509 | 5750.233 | 0.909027 |
| 5791.967 | 1.003294 | 5750.333 | 0.935447 | 5750.333 | 0.922699 |
| 5796.966 | 1.002303 | 5750.433 | 0.937285 | 5750.433 | 0.919692 |
| 5803.633 | 1.01516 | 5750.534 | 0.935545 | 5750.534 | 0.927081 |
| 5813.633 | 1.047518 | 5750.634 | 0.937723 | 5750.634 | 0.940376 |
| 5823.633 | 1.067334 | 5750.733 | 0.938969 | 5750.733 | 0.932483 |
| 5833.633 | 1.075684 | 5750.833 | 0.94303 | 5750.833 | 0.923241 |
| | | 5750.933 | 0.946608 | 5750.933 | 0.923716 |
| | | 5751.033 | 0.94562 | 5751.033 | 0.920971 |
| | | 5751.133 | 0.938252 | 5751.133 | 0.922495 |
| | | 5751.234 | 0.932543 | 5751.234 | 0.922703 |
| | | 5751.333 | 0.939498 | 5751.333 | 0.931715 |
| | | 5751.433 | 0.939022 | 5751.433 | 0.927016 |
| | | 5751.533 | 0.938236 | 5751.533 | 0.927792 |
| | | 5751.633 | 0.943346 | 5751.633 | 0.925826 |
| | | 5751.733 | 0.935429 | 5751.733 | 0.920386 |
| | | 5751.833 | 0.934903 | 5751.833 | 0.921883 |
| | | 5751.934 | 0.937732 | 5751.934 | 0.916605 |
| | | 5752.034 | 0.936149 | 5752.034 | 0.929028 |
| | | 5752.133 | 0.934446 | 5752.133 | 0.930031 |
| | | 5752.233 | 0.943934 | 5752.233 | 0.931024 |
| | | 5752.333 | 0.943384 | 5752.333 | 0.925956 |
| | | 5752.433 | 0.948278 | 5752.433 | 0.934635 |
| | | 5752.533 | 0.951883 | 5752.533 | 0.930169 |
| | | 5752.633 | 0.951526 | 5752.633 | 0.924405 |
| | | 5752.733 | 0.945836 | 5752.733 | 0.940366 |
| | | 5752.833 | 0.941216 | 5752.833 | 0.930106 |
| | | 5752.934 | 0.944962 | 5752.934 | 0.946357 |
| | | 5753.034 | 0.950692 | 5753.034 | 0.949904 |
| | | 5753.134 | 0.946656 | 5753.134 | 0.936351 |
| | | 5753.234 | 0.940079 | 5753.234 | 0.929001 |
| | | 5753.333 | 0.948206 | 5753.333 | 0.953087 |
| | | 5753.433 | 0.955156 | 5753.433 | 0.962826 |
| | | 5753.533 | 0.948252 | 5753.533 | 0.950817 |
| | | 5754.267 | 0.949466 | 5754.267 | 0.947102 |

| | | | | | |
|---|---|---|---|---|---|
| | | 5756.267 | 0.968843 | 5756.267 | 0.966629 |
| | | 5758.267 | 0.97914 | 5758.267 | 0.989946 |
| | | 5760.266 | 0.996083 | 5760.266 | 1.002162 |
| | | 5763.267 | 0.999391 | 5763.267 | 0.994604 |
| | | 5768.267 | 0.993026 | 5768.267 | 0.992082 |
| | | 5773.267 | 0.997287 | 5773.267 | 1.008256 |
| | | 5778.267 | 0.995749 | 5778.267 | 1.002355 |
| | | 5783.267 | 0.993235 | 5783.267 | 1.006403 |
| | | 5788.267 | 0.993585 | 5788.267 | 1.009677 |
| | | 5793.266 | 0.997794 | 5793.266 | 0.988176 |
| | | 5798.267 | 1.00158 | 5798.267 | 1.002879 |
| | | 5804.934 | 1.015628 | 5804.934 | 1.017424 |
| | | 5814.933 | 1.031823 | 5814.933 | 1.03287 |
| | | 5824.933 | 1.04495 | 5824.933 | 1.044159 |
| | | 5834.934 | 1.057768 | 5834.934 | 1.067354 |
| | | 5844.933 | 1.067344 | 5844.933 | 1.071473 |
| | | 5854.933 | 1.072859 | 5854.933 | 1.079334 |
| | | 5864.934 | 1.060723 | 5864.934 | 1.075851 |
| | | 5874.933 | 1.069641 | 5874.933 | 1.072626 |
| | | 5884.933 | 1.092297 | 5884.933 | 1.084406 |

## Additional HERFD and FDMNES results

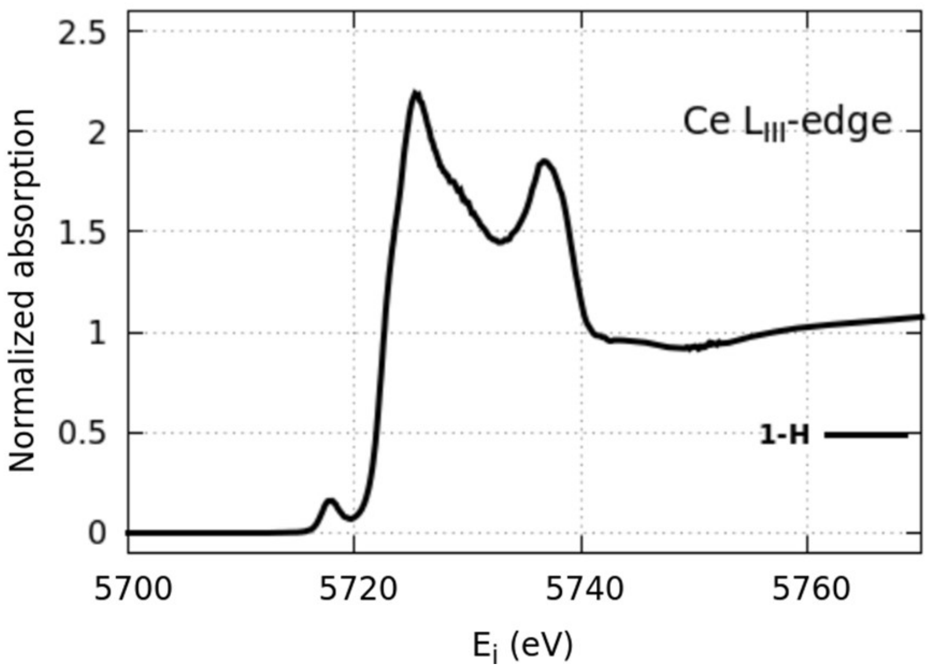


**Figure S17. 1-H** HERFD spectrum.

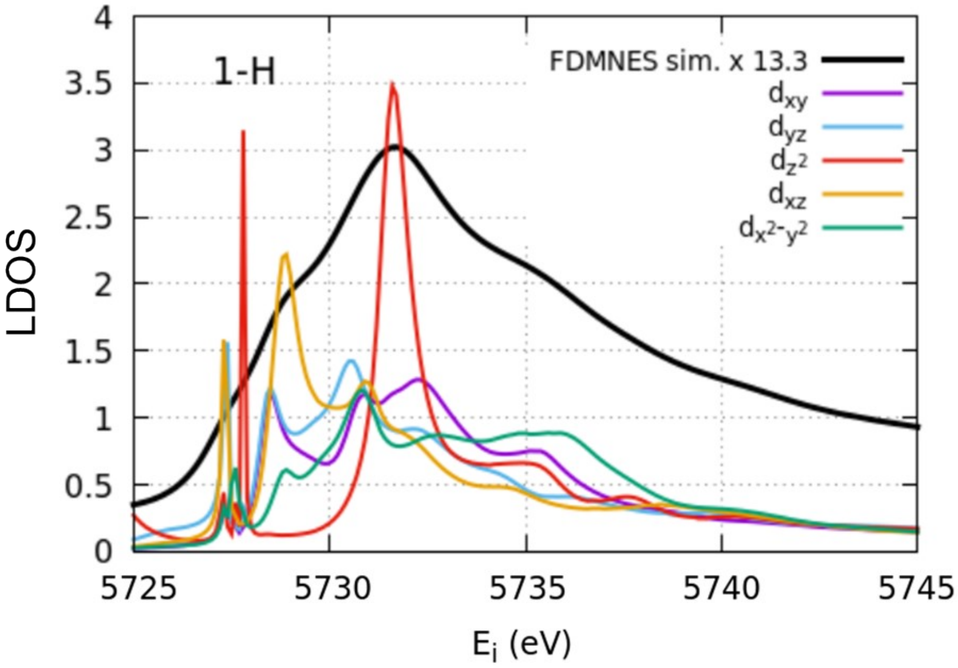


**Figure S18. 1-H** FDMNES simulation and local density of state (LDOS).

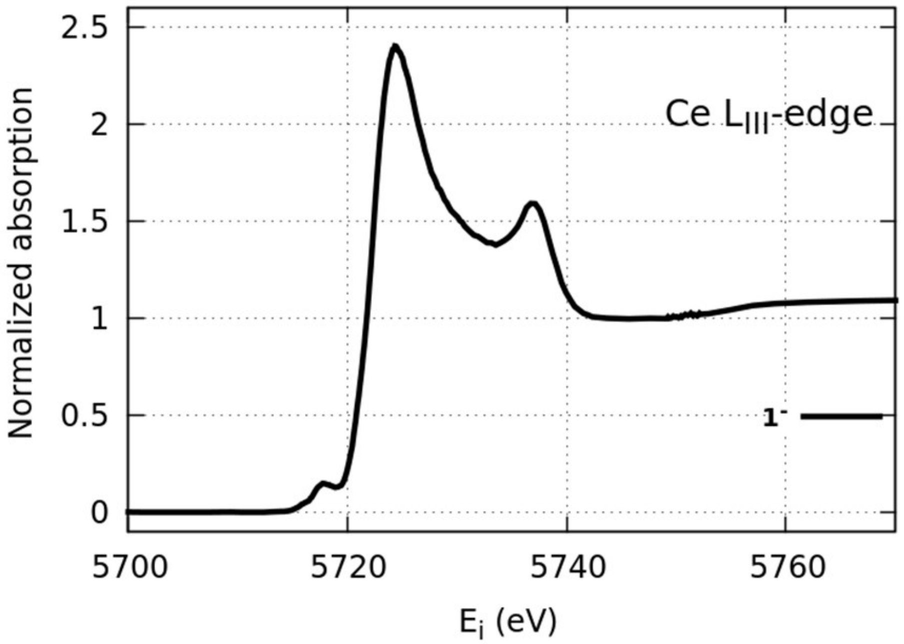


**Figure S19. 1**$^-$ HERFD spectrum.

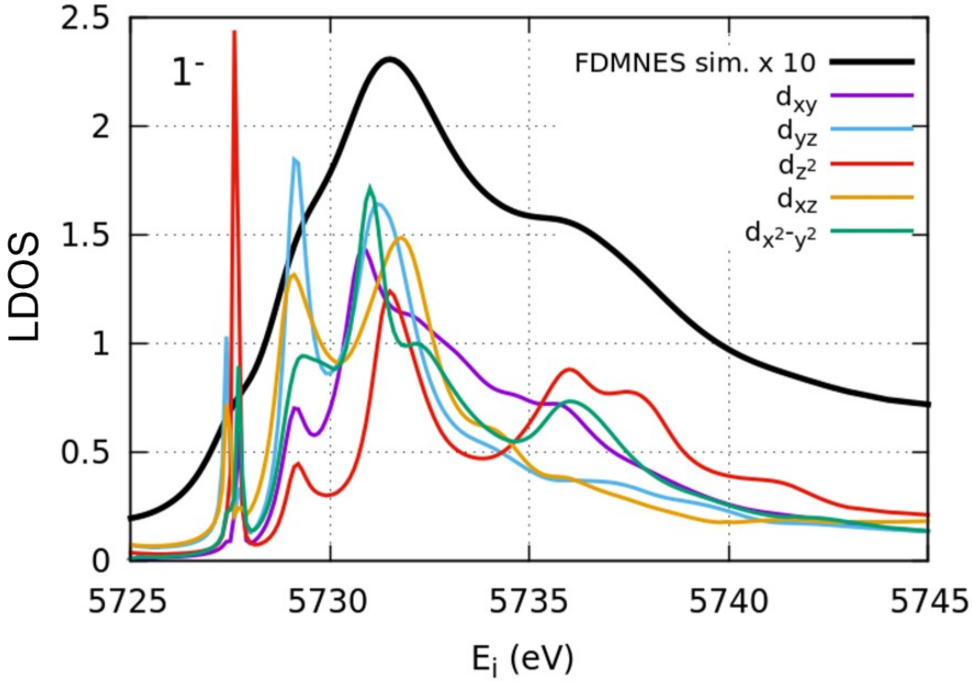


**Figure S20. 1**$^-$ FDMNES simulation and local density of state (LDOS).

## Further details of magnetism results

### *Magnetic susceptibility curves*

Magnetic susceptibility curves for all samples are shown. In cases where more than one data set was collected, average $\chi_0$ values have been reported in Table 1 of the manuscript, with error bars included to represent variation in results. Here, only one representative data set per sample has been shown, with the exception of **1**, given the especially wide variation compared with the other samples, whose results varied minimally between data sets.

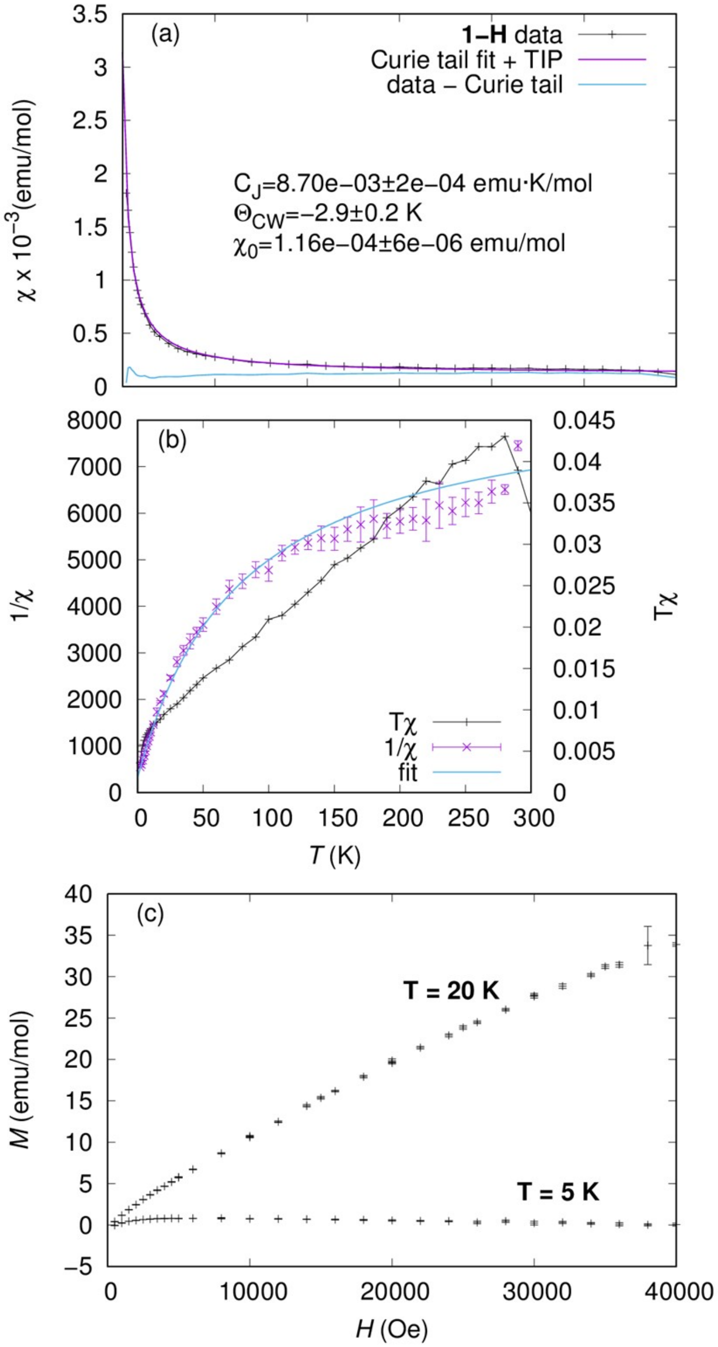


**Figure S21a.** Magnetic susceptibility curves and fitting for **1-H**.

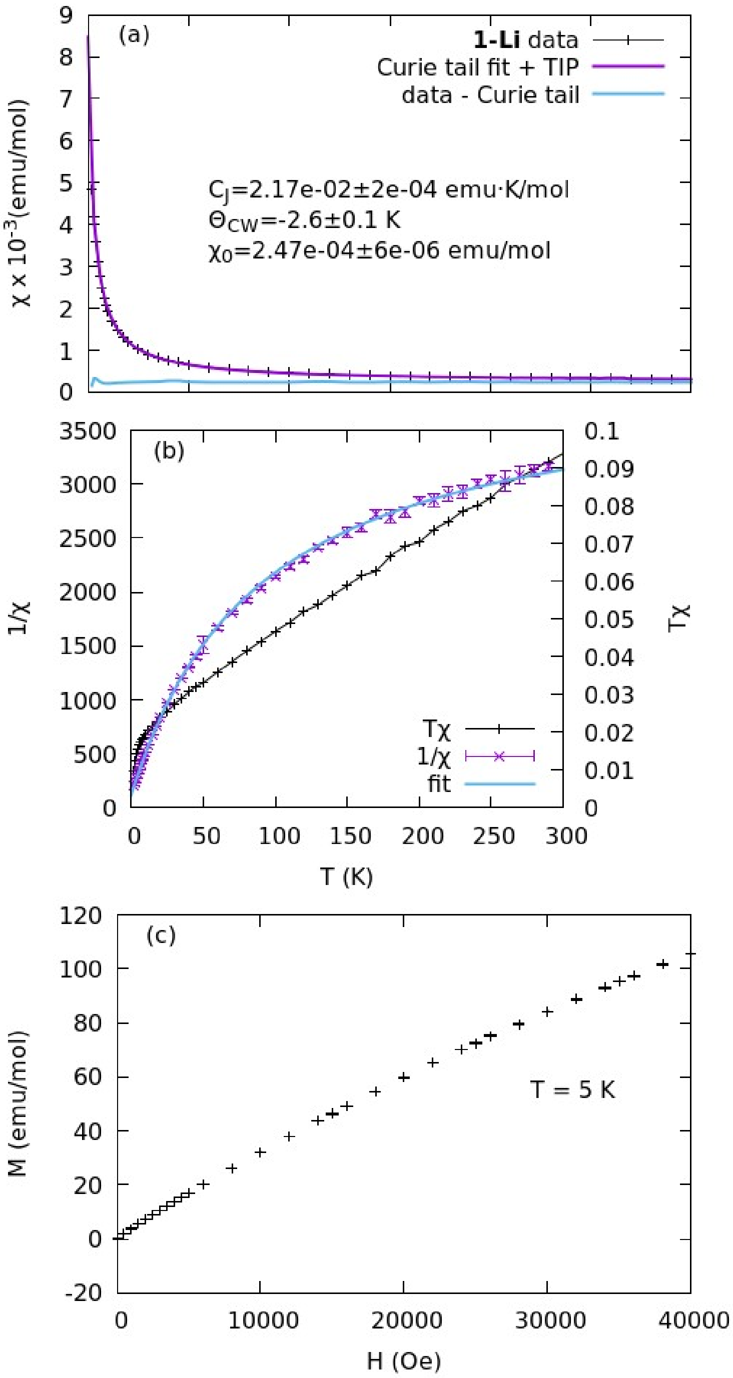


**Figure S21b.** Magnetic susceptibility curves and fitting for **1-Li**.

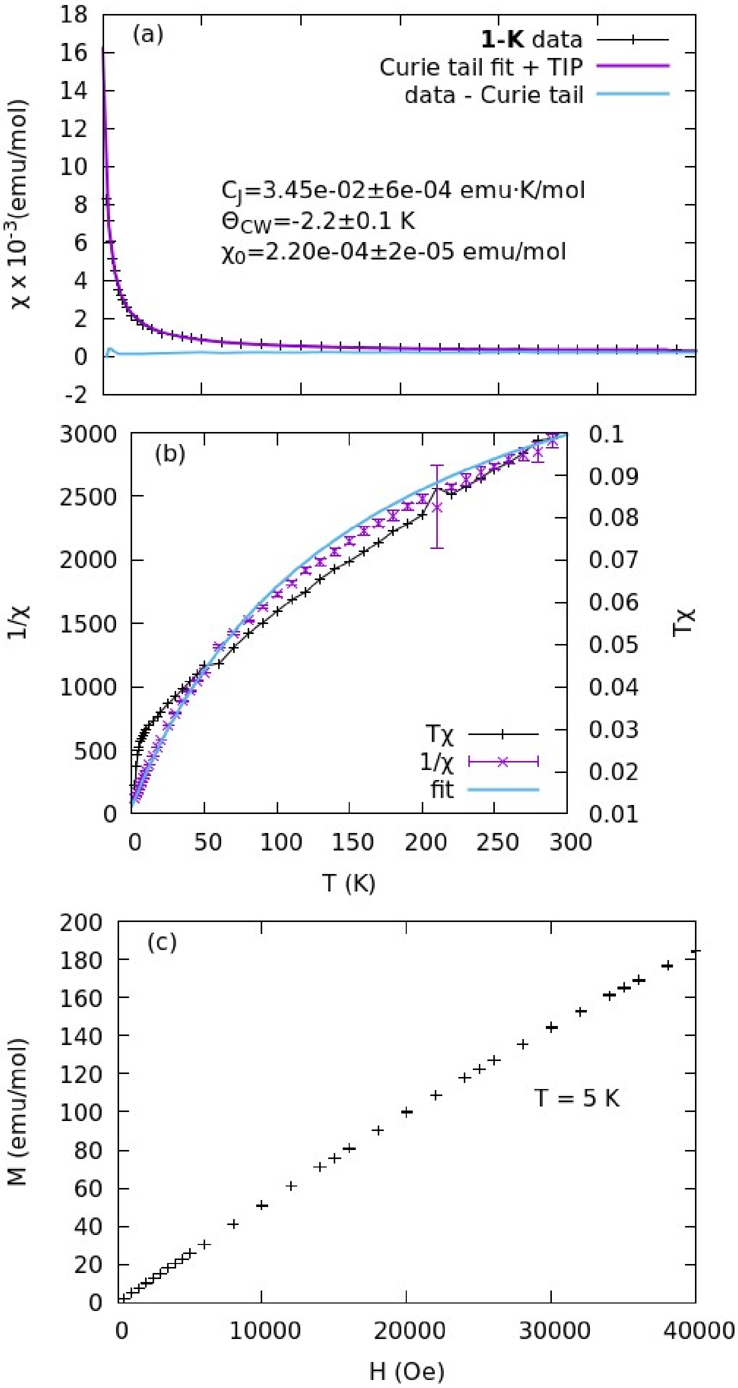


**Figure S21c.** Magnetic susceptibility curves and fitting for **1-K**.

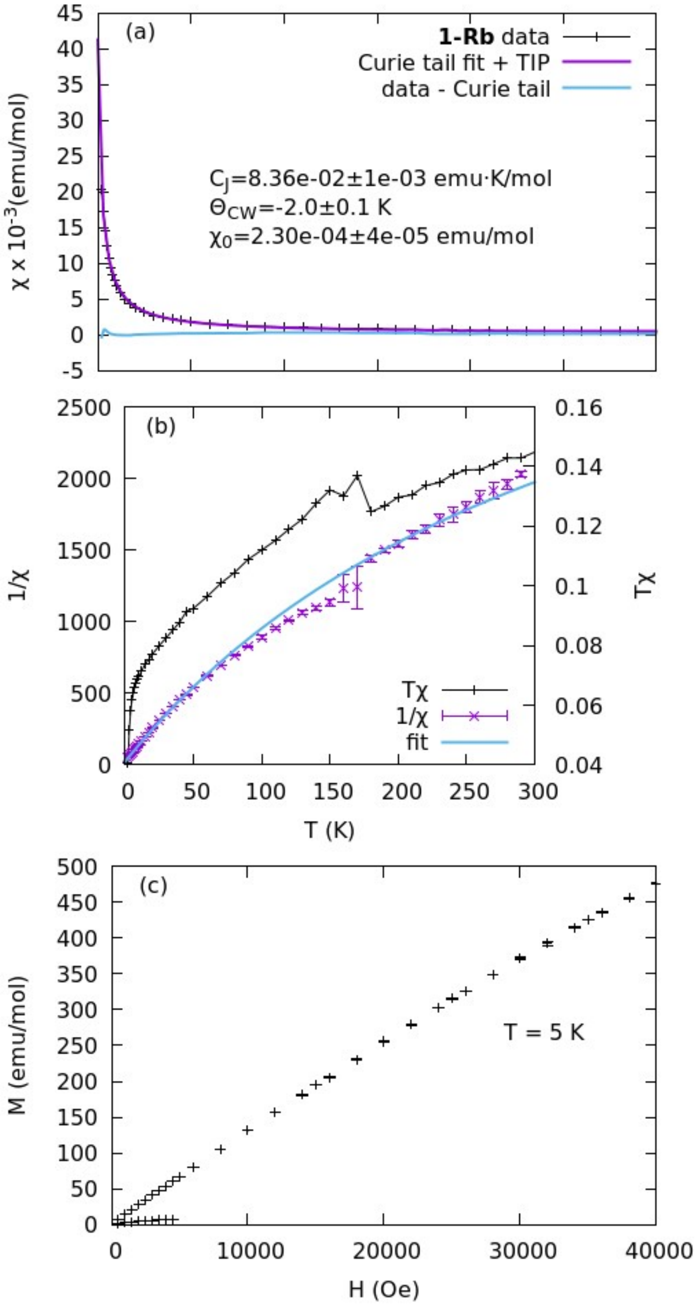


**Figure S21d.** Magnetic susceptibility curves and fitting for **1-Rb**.

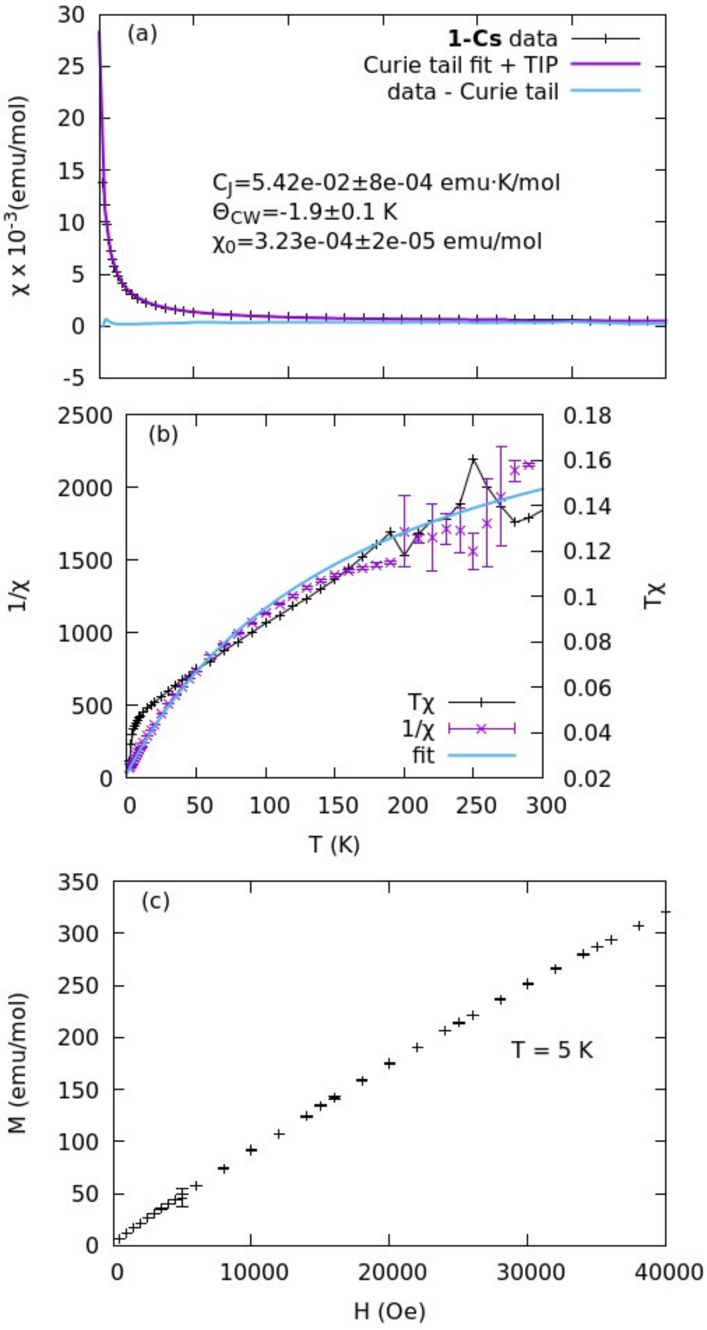


**Figure S21e.** Magnetic susceptibility curves and fitting for **1-Cs**.

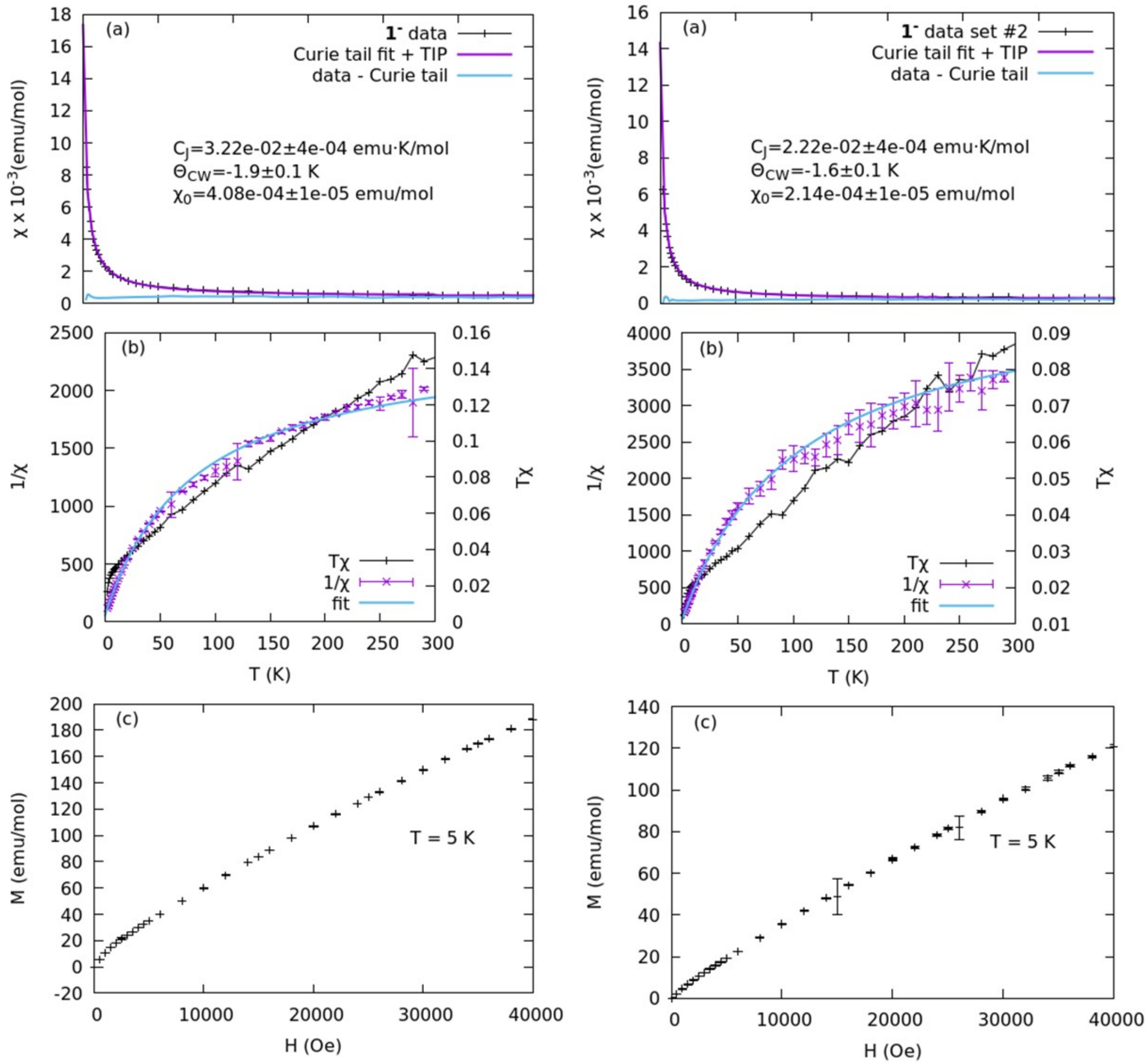


**Figure S21f.** Magnetic susceptibility curves and fitting for **1**$^-$. Here, two different data sets were included from samples synthesized at different times, to represent the wider variation observed in the results for this sample.

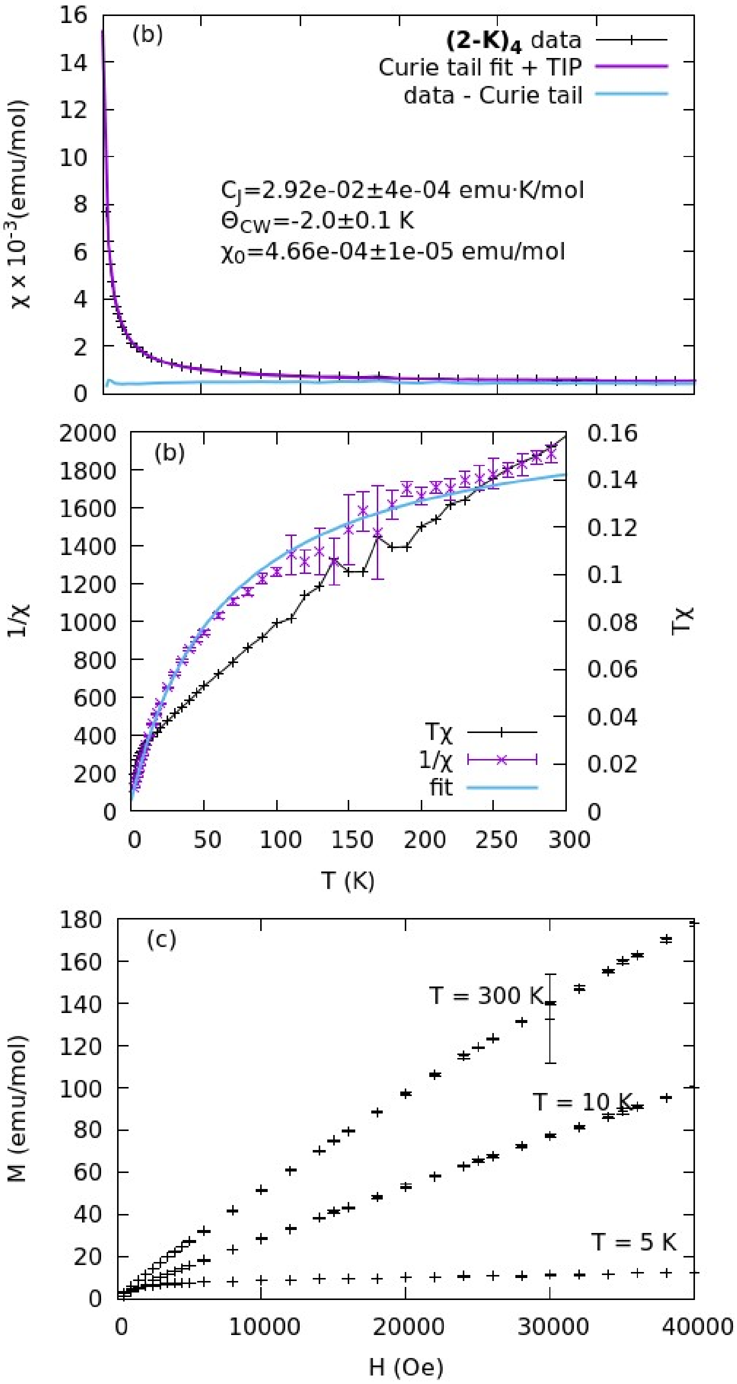


**Figure S21g.** Magnetic susceptibility curves and fitting for **(2-K)$_4$**.

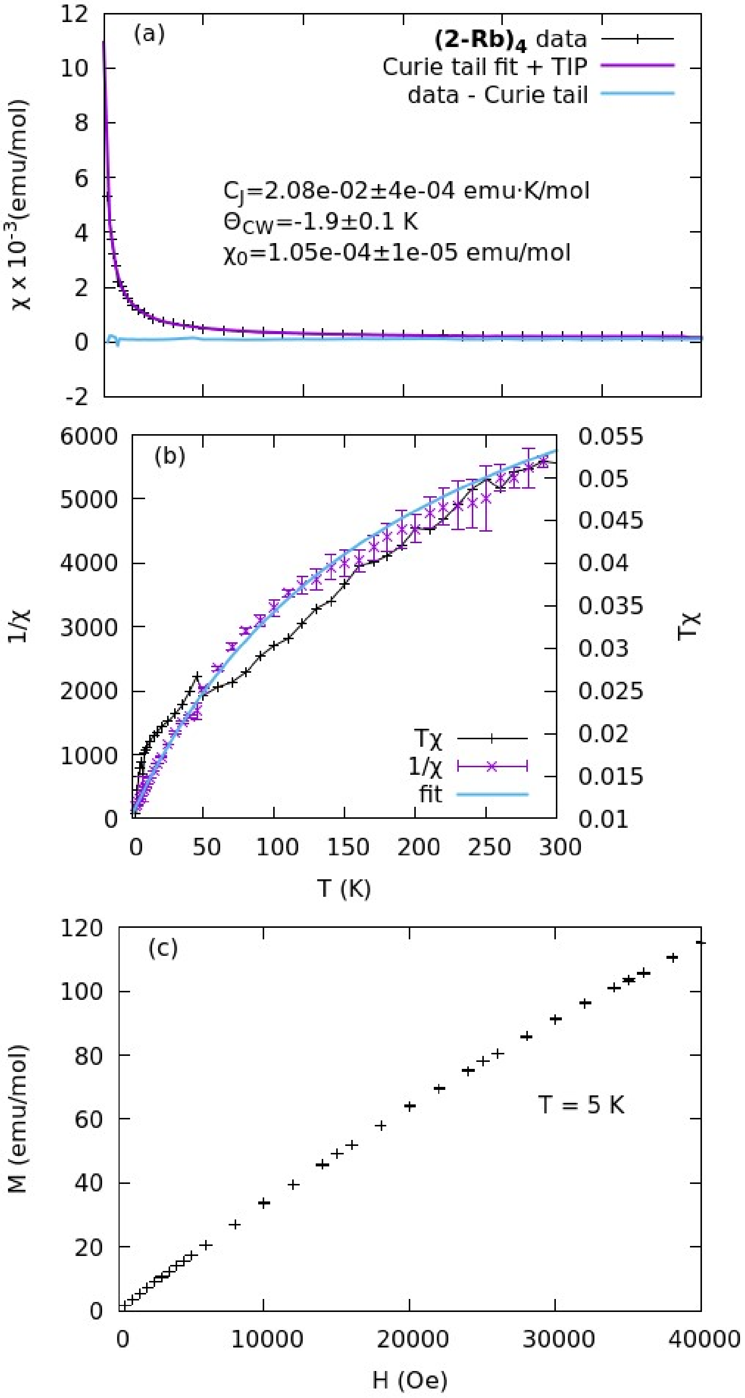


**Figure S21h.** Magnetic susceptibility curves and fitting for **(2-Rb)**$_4$.

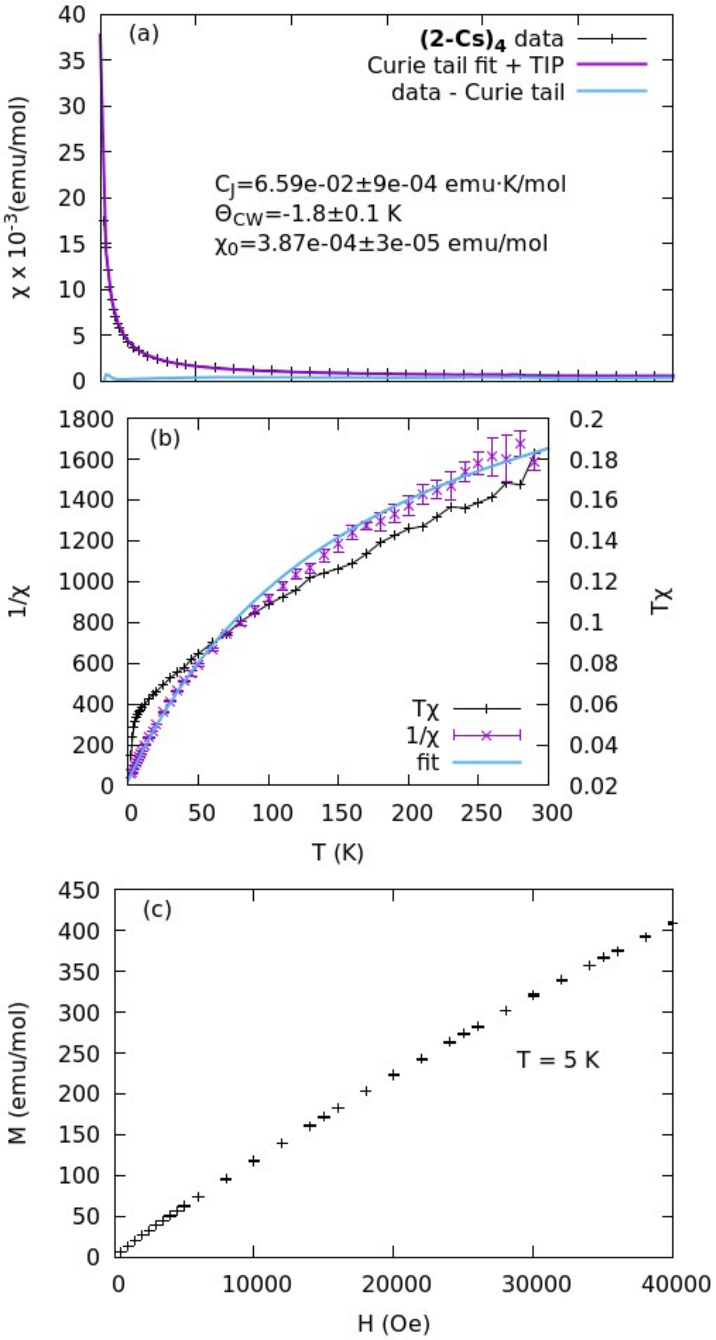


**Figure S21i.** Magnetic susceptibility curves and fitting for **(2-Cs)$_4$**.

### *Evans Method Analysis*

**Evans measurement of (2-K)$_4$ susceptibility:** 1.9 mM solution of (2-K)$_4$ in THF-*d8* was used. $\Delta_{ppm}$ = 0.025 ppm was observed. $\chi_m$ = 0.0033 emu/mol.

**Evans measurement of (2-Cs)$_4$ susceptibility:** 2.85 mM solution of (2-Cs)$_4$ in THF-*d8* was used. Peak splitting was not observable by $^1$H NMR.

## Further details of computational results

**Figure S22**. Restricted Open-Shell Hartree-Fock molecular orbitals used to build the Complete Active Space (CAS) for each Ce imido and oxo complex. Spin-multiplicity values are shown in brackets.

**1-K:** **[Triplet]**

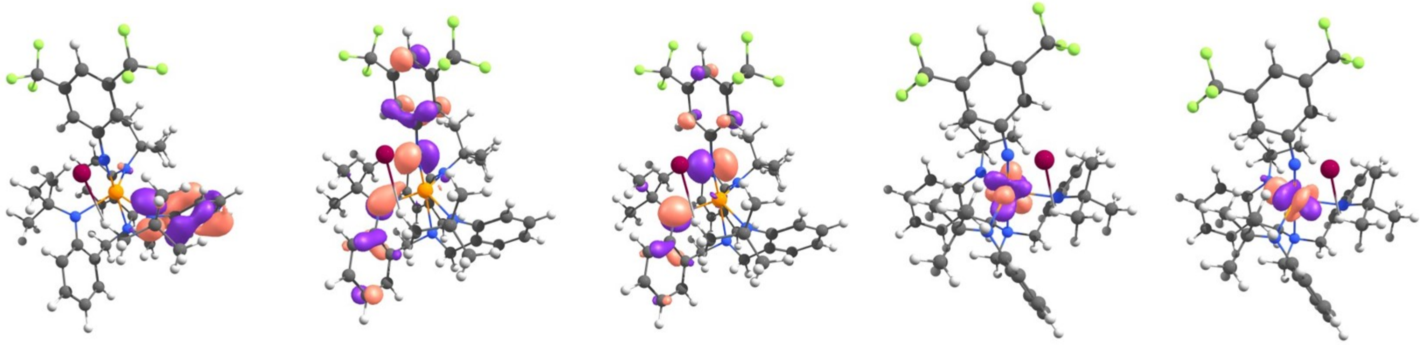

**1-Rb:** **[Singlet]**

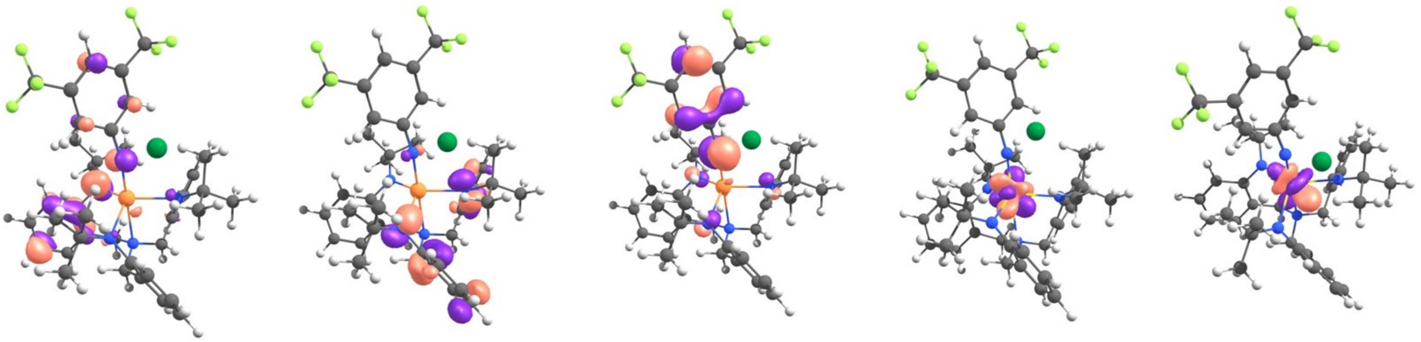

**1-Cs** **[Singlet]**

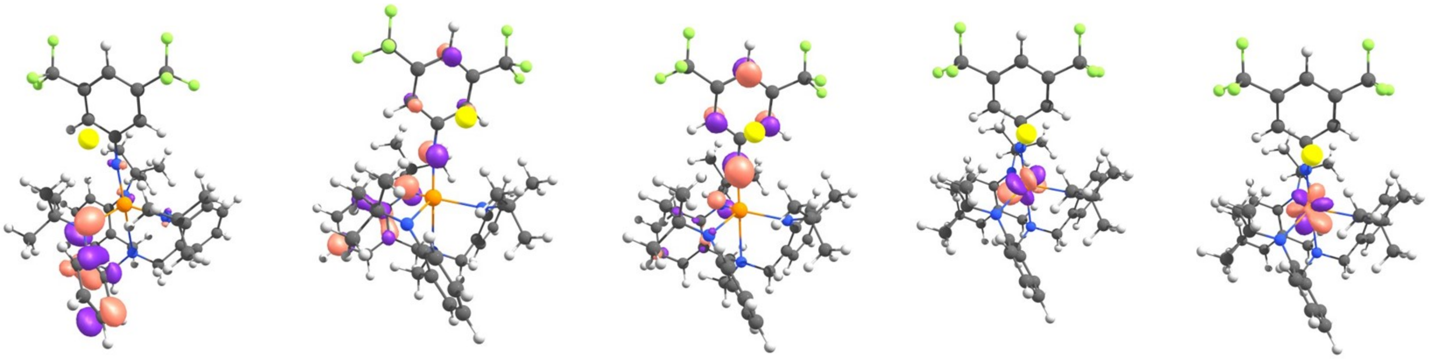

HOMO-2 HOMO-1 HOMO LUMO+
LUMO+18

**(2-K)$_4$:** **[Quintet]**

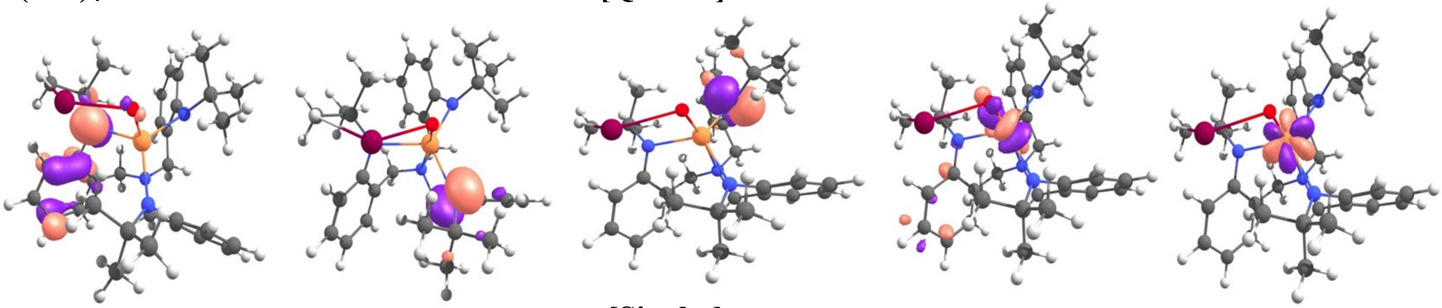

**(2-Rb)$_4$:** **[Singlet]**

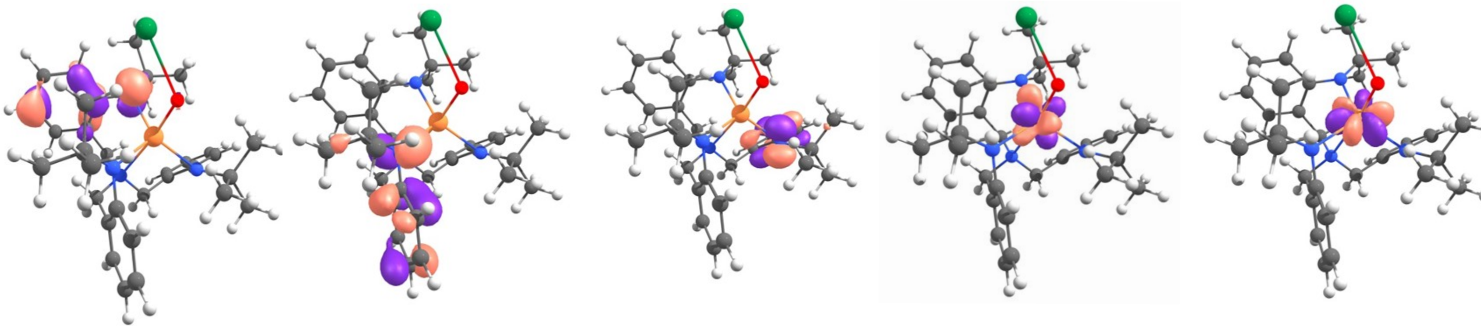

**(2-Cs)$_4$:** **[Singlet]**

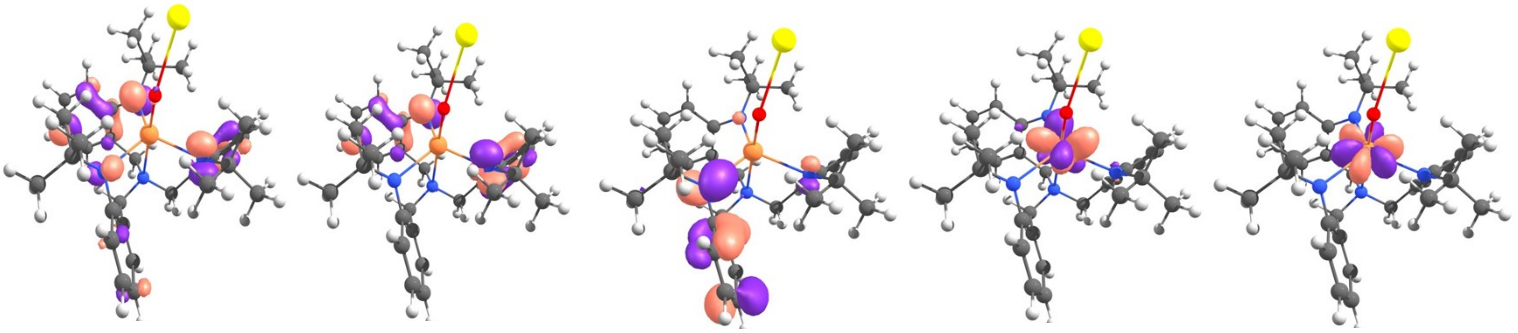

*Cartesian coordinates of all optimized structures*

---

**1-K**

| | | | |
|---|---|---|---|
| C | 14.302878 | 7.008390 | 23.715996 |
| C | 14.949615 | 8.200888 | 24.057827 |
| C | 16.282971 | 8.444600 | 23.618198 |
| C | 16.901219 | 7.417483 | 22.863749 |
| C | 16.246860 | 6.232327 | 22.552165 |
| C | 14.932151 | 6.019567 | 22.966817 |
| C | 14.280901 | 9.171490 | 24.993263 |
| N | 13.767685 | 10.397101 | 24.348188 |
| C | 13.379911 | 11.394805 | 25.365521 |
| C | 14.514690 | 11.934760 | 26.196521 |
| C | 14.626810 | 11.546597 | 27.535603 |
| C | 15.553451 | 12.125232 | 28.397533 |
| C | 16.380186 | 13.133586 | 27.902481 |
| C | 16.299345 | 13.519290 | 26.569991 |
| C | 15.385417 | 12.928549 | 25.662166 |
| N | 15.309174 | 13.245967 | 24.306881 |
| C | 15.266962 | 14.667851 | 23.890156 |
| C | 14.501706 | 14.707432 | 22.558722 |
| N | 16.880296 | 9.669119 | 23.919962 |
| C | 18.257500 | 9.700939 | 24.449954 |
| C | 18.354307 | 10.987608 | 25.287136 |
| C | 12.625233 | 10.077946 | 23.467184 |
| C | 12.167195 | 11.218575 | 22.596714 |
| C | 12.918661 | 11.592084 | 21.447766 |
| C | 12.373027 | 12.607149 | 20.628086 |
| C | 11.154576 | 13.210547 | 20.913946 |
| C | 10.432090 | 12.841289 | 22.048363 |
| C | 10.951723 | 11.852620 | 22.877325 |
| N | 14.161212 | 10.989223 | 21.212213 |
| Ce | 15.715507 | 11.435927 | 22.834691 |
| N | 17.230086 | 12.313722 | 21.690379 |
| K | 19.544858 | 13.228678 | 22.978052 |
| C | 14.437777 | 10.336617 | 19.909670 |
| C | 15.708765 | 9.500915 | 20.112881 |
| C | 14.702086 | 11.320185 | 18.750267 |
| C | 13.286715 | 9.394726 | 19.521552 |
| C | 19.333672 | 9.755788 | 23.341532 |
| C | 18.560519 | 8.526030 | 25.395102 |
| C | 16.668006 | 15.269291 | 23.629360 |
| C | 14.504222 | 15.551813 | 24.890612 |
| H | 11.779833 | 9.723986 | 24.083463 |

| | | | |
|---|---|---|---|
| H | 12.945055 | 9.248850 | 22.828819 |
| H | 10.392505 | 11.539787 | 23.757262 |
| H | 9.481592 | 13.312484 | 22.281023 |
| H | 10.776164 | 13.987643 | 20.254191 |
| H | 12.939232 | 12.947128 | 19.769989 |
| H | 16.558063 | 10.139307 | 20.393166 |
| H | 15.989154 | 8.988497 | 19.186643 |
| H | 15.559815 | 8.740186 | 20.884512 |
| H | 13.787857 | 11.807762 | 18.402680 |
| H | 15.120627 | 10.782732 | 17.891257 |
| H | 15.419965 | 12.088374 | 19.051997 |
| H | 13.136748 | 8.630373 | 20.291058 |
| H | 13.508170 | 8.886910 | 18.575960 |
| H | 12.349241 | 9.945724 | 19.396414 |
| H | 12.618243 | 10.953418 | 26.032663 |
| H | 12.914200 | 12.226585 | 24.827684 |
| H | 13.941295 | 10.791074 | 27.915311 |
| H | 15.614102 | 11.812864 | 29.435754 |
| H | 17.100343 | 13.619591 | 28.557102 |
| H | 16.968766 | 14.294047 | 26.211444 |
| H | 14.980685 | 14.063312 | 21.810064 |
| H | 14.477768 | 15.722456 | 22.145319 |
| H | 13.472339 | 14.366151 | 22.692564 |
| H | 17.285858 | 15.286030 | 24.534117 |
| H | 16.592096 | 16.301137 | 23.266544 |
| H | 17.155357 | 14.670210 | 22.851719 |
| H | 13.506219 | 15.140525 | 25.072721 |
| H | 14.386343 | 16.561958 | 24.483471 |
| H | 15.012810 | 15.634227 | 25.854445 |
| H | 13.453460 | 8.666553 | 25.522358 |
| H | 15.013007 | 9.497026 | 25.738263 |
| H | 13.289178 | 6.844818 | 24.077360 |
| H | 14.412729 | 5.098379 | 22.720234 |
| H | 16.763736 | 5.476042 | 21.966175 |
| H | 17.908178 | 7.570835 | 22.492417 |
| H | 18.010729 | 11.861649 | 24.716527 |
| H | 19.380009 | 11.165222 | 25.639318 |
| H | 17.700130 | 10.935197 | 26.160146 |
| H | 19.456807 | 8.796075 | 22.833738 |
| H | 20.319340 | 10.020948 | 23.751701 |
| H | 19.025701 | 10.482179 | 22.581009 |
| H | 17.824395 | 8.488484 | 26.204221 |
| H | 19.554386 | 8.644236 | 25.842290 |
| H | 18.538724 | 7.565114 | 24.875548 |
| C | 17.904212 | 12.795834 | 20.622443 |
| C | 19.044704 | 12.130167 | 20.075416 |

| | | | |
|---|---|---|---|
| C | 19.821887 | 12.697960 | 19.067522 |
| C | 19.510998 | 13.938583 | 18.516729 |
| C | 18.375081 | 14.595970 | 19.003791 |
| C | 17.594895 | 14.055595 | 20.015603 |
| H | 19.289929 | 11.138707 | 20.447501 |
| C | 21.067044 | 11.972553 | 18.643207 |
| H | 20.115382 | 14.370357 | 17.729330 |
| C | 18.050344 | 15.967359 | 18.478400 |
| H | 16.720251 | 14.592627 | 20.366284 |
| F | 16.742936 | 16.264567 | 18.588250 |
| F | 18.725568 | 16.922156 | 19.163785 |
| F | 18.391328 | 16.108431 | 17.183843 |
| F | 21.600415 | 12.466182 | 17.513795 |
| F | 22.026414 | 12.068231 | 19.604431 |
| F | 20.854607 | 10.657397 | 18.453914 |

**1-Rb**

| | | | |
|---|---|---|---|
| C | 14.299032 | 7.032337 | 23.765430 |
| C | 14.949165 | 8.227576 | 24.090374 |
| C | 16.275490 | 8.470379 | 23.629828 |
| C | 16.884124 | 7.439875 | 22.872410 |
| C | 16.226723 | 6.252143 | 22.577287 |
| C | 14.918659 | 6.039955 | 23.012806 |
| C | 14.292875 | 9.204073 | 25.028414 |
| N | 13.771555 | 10.425272 | 24.381998 |
| C | 13.393689 | 11.428288 | 25.397600 |
| C | 14.536840 | 11.979857 | 26.209008 |
| C | 14.670677 | 11.600372 | 27.548466 |
| C | 15.603828 | 12.191887 | 28.394602 |
| C | 16.413920 | 13.205112 | 27.882494 |
| C | 16.311835 | 13.582564 | 26.549096 |
| C | 15.392000 | 12.977714 | 25.656077 |
| N | 15.293962 | 13.283332 | 24.300397 |
| C | 15.249227 | 14.700036 | 23.868752 |
| C | 14.498044 | 14.722456 | 22.528717 |
| N | 16.875155 | 9.697103 | 23.916963 |
| C | 18.257004 | 9.734402 | 24.434331 |
| C | 18.362490 | 11.032753 | 25.251345 |
| C | 12.619559 | 10.099967 | 23.516159 |
| C | 12.145261 | 11.235634 | 22.648003 |
| C | 12.879155 | 11.606120 | 21.486492 |
| C | 12.312293 | 12.608223 | 20.664813 |
| C | 11.093957 | 13.205533 | 20.963525 |
| C | 10.391176 | 12.842336 | 22.112222 |

| | | | |
|---|---|---|---|
| C | 10.929706 | 11.863651 | 22.941055 |
| N | 14.120455 | 11.008309 | 21.238587 |
| Ce | 15.702547 | 11.460278 | 22.841480 |
| N | 17.216522 | 12.299849 | 21.673693 |
| Rb | 19.757997 | 13.373854 | 22.897538 |
| C | 14.389849 | 10.370857 | 19.926436 |
| C | 15.627442 | 9.486635 | 20.130641 |
| C | 14.706324 | 11.368036 | 18.791492 |
| C | 13.213986 | 9.476282 | 19.503026 |
| C | 19.324465 | 9.775346 | 23.317293 |
| C | 18.564423 | 8.570110 | 25.390943 |
| C | 16.648342 | 15.307950 | 23.613973 |
| C | 14.471611 | 15.587084 | 24.854743 |
| H | 11.783380 | 9.744176 | 24.143967 |
| H | 12.934979 | 9.270629 | 22.875770 |
| H | 10.384943 | 11.553074 | 23.830851 |
| H | 9.441024 | 13.309127 | 22.354961 |
| H | 10.699639 | 13.972984 | 20.301706 |
| H | 12.861474 | 12.942644 | 19.793516 |
| H | 16.494539 | 10.089979 | 20.433267 |
| H | 15.903357 | 8.981132 | 19.199181 |
| H | 15.441983 | 8.720673 | 20.888897 |
| H | 13.816515 | 11.898496 | 18.443175 |
| H | 15.115627 | 10.834412 | 17.925568 |
| H | 15.447964 | 12.101942 | 19.119323 |
| H | 13.022558 | 8.707449 | 20.258723 |
| H | 13.437358 | 8.974300 | 18.554850 |
| H | 12.298240 | 10.060070 | 19.367664 |
| H | 12.643122 | 10.988334 | 26.078252 |
| H | 12.917338 | 12.254205 | 24.860080 |
| H | 13.996502 | 10.841614 | 27.941836 |
| H | 15.681488 | 11.885962 | 29.433624 |
| H | 17.138171 | 13.701753 | 28.524606 |
| H | 16.969337 | 14.361078 | 26.177848 |
| H | 14.998074 | 14.088436 | 21.784700 |
| H | 14.458872 | 15.736637 | 22.114497 |
| H | 13.473960 | 14.361978 | 22.652304 |
| H | 17.250379 | 15.367042 | 24.527101 |
| H | 16.566772 | 16.324208 | 23.210181 |
| H | 17.156226 | 14.682927 | 22.871230 |
| H | 13.470635 | 15.176718 | 25.021852 |
| H | 14.361024 | 16.597190 | 24.445245 |
| H | 14.966177 | 15.669076 | 25.825999 |
| H | 13.472243 | 8.702589 | 25.571423 |
| H | 15.034670 | 9.534526 | 25.761670 |
| H | 13.290771 | 6.869956 | 24.142232 |

| | | | |
|---|---|---|---|
| H | 14.396857 | 5.116733 | 22.779079 |
| H | 16.735775 | 5.493178 | 21.987876 |
| H | 17.885363 | 7.592552 | 22.485709 |
| H | 18.042909 | 11.900616 | 24.658155 |
| H | 19.387623 | 11.201011 | 25.609744 |
| H | 17.699598 | 11.003474 | 26.118884 |
| H | 19.435418 | 8.812724 | 22.812149 |
| H | 20.315134 | 10.031768 | 23.721311 |
| H | 19.019310 | 10.505473 | 22.559511 |
| H | 17.837188 | 8.546434 | 26.208631 |
| H | 19.563856 | 8.688090 | 25.825672 |
| H | 18.531460 | 7.603071 | 24.883228 |
| C | 17.903636 | 12.728992 | 20.594635 |
| C | 18.925345 | 11.944036 | 19.972609 |
| C | 19.731600 | 12.450010 | 18.957158 |
| C | 19.572503 | 13.748511 | 18.472364 |
| C | 18.553607 | 14.525722 | 19.032222 |
| C | 17.740925 | 14.043522 | 20.050110 |
| H | 19.059414 | 10.919014 | 20.305999 |
| C | 20.859448 | 11.596241 | 18.450376 |
| H | 20.200058 | 14.132621 | 17.678496 |
| C | 18.400471 | 15.955361 | 18.593216 |
| H | 16.952921 | 14.670586 | 20.453867 |
| F | 17.134619 | 16.396024 | 18.702366 |
| F | 19.163522 | 16.778609 | 19.358327 |
| F | 18.789258 | 16.145312 | 17.319505 |
| F | 21.349897 | 12.025971 | 17.275406 |
| F | 21.896915 | 11.602129 | 19.331330 |
| F | 20.501577 | 10.308825 | 18.296880 |

**1-Cs**

| | | | |
|---|---|---|---|
| C | 14.431524 | 6.926392 | 23.860361 |
| C | 15.060192 | 8.157941 | 24.075285 |
| C | 16.266482 | 8.471575 | 23.393998 |
| C | 16.761092 | 7.506265 | 22.487876 |
| C | 16.131478 | 6.281510 | 22.297102 |
| C | 14.958852 | 5.979495 | 22.988603 |
| C | 14.472374 | 9.134057 | 25.058623 |
| N | 13.861505 | 10.322266 | 24.430434 |
| C | 13.530340 | 11.330007 | 25.457738 |
| C | 14.719174 | 11.940957 | 26.151169 |
| C | 14.990735 | 11.597526 | 27.478907 |
| C | 15.984016 | 12.232315 | 28.220284 |
| C | 16.715837 | 13.249335 | 27.610233 |

| | | | |
|---|---|---|---|
| C | 16.473653 | 13.596384 | 26.286203 |
| C | 15.482695 | 12.955049 | 25.498545 |
| N | 15.231352 | 13.239420 | 24.160890 |
| C | 15.121745 | 14.644228 | 23.708451 |
| C | 14.277634 | 14.620860 | 22.424482 |
| N | 16.871503 | 9.731074 | 23.571561 |
| C | 18.200371 | 9.789361 | 24.211826 |
| C | 18.620822 | 11.266148 | 24.218594 |
| C | 12.657132 | 9.938681 | 23.662692 |
| C | 12.035336 | 11.046486 | 22.855216 |
| C | 12.600581 | 11.435092 | 21.604678 |
| C | 11.861735 | 12.372870 | 20.841326 |
| C | 10.659338 | 12.907244 | 21.285503 |
| C | 10.133564 | 12.540131 | 22.524141 |
| C | 10.830864 | 11.611169 | 23.288807 |
| N | 13.831935 | 10.907779 | 21.222212 |
| Ce | 15.575607 | 11.427610 | 22.666720 |
| N | 16.920234 | 12.318092 | 21.341877 |
| Cs | 20.103109 | 14.355764 | 22.524963 |
| C | 14.057752 | 10.439351 | 19.831382 |
| C | 15.272854 | 9.500705 | 19.898104 |
| C | 14.403151 | 11.564601 | 18.832024 |
| C | 12.856487 | 9.631407 | 19.316039 |
| C | 19.300689 | 8.991466 | 23.483968 |
| C | 18.124190 | 9.288093 | 25.666561 |
| C | 16.483587 | 15.280339 | 23.338356 |
| C | 14.396603 | 15.528338 | 24.735619 |
| H | 11.903480 | 9.526463 | 24.357284 |
| H | 12.962995 | 9.137285 | 22.982803 |
| H | 10.419516 | 11.285031 | 24.242522 |
| H | 9.196021 | 12.957648 | 22.879272 |
| H | 10.135199 | 13.627388 | 20.661297 |
| H | 12.260130 | 12.702973 | 19.890497 |
| H | 16.170509 | 10.041700 | 20.229749 |
| H | 15.501954 | 9.089619 | 18.908727 |
| H | 15.085740 | 8.668581 | 20.581985 |
| H | 13.541313 | 12.186171 | 18.575814 |
| H | 14.773577 | 11.133563 | 17.894319 |
| H | 15.186042 | 12.205236 | 19.248209 |
| H | 12.646908 | 8.790144 | 19.984925 |
| H | 13.068066 | 9.231487 | 18.317992 |
| H | 11.952625 | 10.243486 | 19.248199 |
| H | 12.862713 | 10.875229 | 26.211297 |
| H | 12.974072 | 12.124179 | 24.950590 |
| H | 14.378958 | 10.830455 | 27.950583 |
| H | 16.168480 | 11.952593 | 29.253146 |

| | | | |
|---|---|---|---|
| H | 17.487399 | 13.776023 | 28.168129 |
| H | 17.069924 | 14.384152 | 25.839283 |
| H | 14.772779 | 14.033935 | 21.639553 |
| H | 14.135108 | 15.633275 | 22.029352 |
| H | 13.294697 | 14.184336 | 22.618546 |
| H | 17.121704 | 15.429935 | 24.216464 |
| H | 16.340021 | 16.260224 | 22.866202 |
| H | 16.977991 | 14.614184 | 22.622398 |
| H | 13.409772 | 15.111599 | 24.960607 |
| H | 14.256033 | 16.538151 | 24.334079 |
| H | 14.947621 | 15.612237 | 25.676358 |
| H | 13.718591 | 8.621571 | 25.682764 |
| H | 15.260468 | 9.500375 | 25.720611 |
| H | 13.512346 | 6.708384 | 24.400814 |
| H | 14.456910 | 5.027521 | 22.841270 |
| H | 16.547815 | 5.569131 | 21.589047 |
| H | 17.639784 | 7.748128 | 21.901063 |
| H | 18.629056 | 11.635981 | 23.185140 |
| H | 19.618761 | 11.376242 | 24.662746 |
| H | 17.922355 | 11.870057 | 24.808731 |
| H | 19.119668 | 7.914891 | 23.531638 |
| H | 20.273673 | 9.177993 | 23.954981 |
| H | 19.369579 | 9.284593 | 22.431050 |
| H | 17.450286 | 9.915877 | 26.257551 |
| H | 19.111507 | 9.305324 | 26.144291 |
| H | 17.756699 | 8.256897 | 25.697338 |
| C | 17.868923 | 12.752516 | 20.517108 |
| C | 19.052600 | 11.988681 | 20.227620 |
| C | 20.114492 | 12.520603 | 19.508596 |
| C | 20.084525 | 13.823861 | 18.994893 |
| C | 18.915714 | 14.567410 | 19.205421 |
| C | 17.838195 | 14.060821 | 19.919671 |
| H | 19.108253 | 10.977853 | 20.618745 |
| C | 21.398779 | 11.748480 | 19.427997 |
| H | 20.914270 | 14.225309 | 18.426450 |
| C | 18.904593 | 16.010872 | 18.794680 |
| H | 16.950571 | 14.666974 | 20.068888 |
| F | 17.673101 | 16.492670 | 18.577792 |
| F | 19.448139 | 16.778134 | 19.793627 |
| F | 19.641741 | 16.250129 | 17.698030 |
| F | 22.119385 | 12.042419 | 18.333756 |
| F | 22.192722 | 12.060892 | 20.502608 |
| F | 21.223943 | 10.420217 | 19.462978 |

**(2-K)$_4$**

| | | | |
|---|---|---|---|
| C | 15.918138 | 8.233170 | 23.816977 |
| C | 14.534249 | 8.218580 | 24.191826 |
| C | 13.726931 | 7.122176 | 23.869694 |
| C | 14.215143 | 6.008370 | 23.193896 |
| C | 15.564919 | 5.995555 | 22.843181 |
| C | 16.388474 | 7.072233 | 23.142509 |
| C | 13.959344 | 9.300000 | 25.068067 |
| N | 13.607054 | 10.564046 | 24.378857 |
| C | 13.205113 | 11.596915 | 25.356879 |
| C | 14.304912 | 11.996089 | 26.301982 |
| C | 14.252789 | 11.582361 | 27.637338 |
| C | 15.230775 | 11.949595 | 28.555243 |
| C | 16.300382 | 12.728615 | 28.115177 |
| C | 16.373487 | 13.139274 | 26.789150 |
| C | 15.372242 | 12.815526 | 25.842006 |
| N | 15.426226 | 13.199207 | 24.492546 |
| C | 15.405900 | 14.640552 | 24.177801 |
| C | 14.103697 | 15.281483 | 24.694904 |
| N | 16.681975 | 9.348629 | 24.104096 |
| C | 18.122896 | 9.244584 | 24.434055 |
| C | 18.392614 | 8.107179 | 25.433138 |
| Ce | 15.840951 | 11.327945 | 23.112880 |
| N | 14.354873 | 11.042080 | 21.164666 |
| C | 14.405789 | 10.029902 | 20.081972 |
| C | 13.378129 | 8.901057 | 20.305322 |
| O | 17.243681 | 11.975107 | 22.060538 |
| C | 13.148246 | 11.746360 | 21.341365 |
| C | 12.288271 | 11.498809 | 22.451056 |
| C | 11.150334 | 12.290805 | 22.648603 |
| C | 10.805241 | 13.323860 | 21.783034 |
| C | 11.621573 | 13.572816 | 20.682406 |
| C | 12.762401 | 12.803017 | 20.479633 |
| C | 12.537851 | 10.342602 | 23.388986 |
| C | 15.804327 | 9.398198 | 20.091485 |
| C | 14.132985 | 10.586518 | 18.662956 |
| C | 15.438195 | 14.769564 | 22.647327 |
| C | 16.601341 | 15.455149 | 24.717875 |
| C | 18.490822 | 10.569210 | 25.126489 |
| C | 19.046079 | 9.113624 | 23.201347 |
| K | 16.304144 | 12.972067 | 19.919808 |
| H | 11.597999 | 10.098369 | 23.916034 |
| H | 12.824805 | 9.463775 | 22.810350 |
| H | 10.510225 | 12.074239 | 23.501744 |
| H | 9.914351 | 13.918747 | 21.961357 |

| | | | |
|---|---|---|---|
| H | 11.375702 | 14.370252 | 19.984974 |
| H | 13.380924 | 13.014646 | 19.609390 |
| H | 16.601393 | 10.140621 | 19.967280 |
| H | 15.902504 | 8.661236 | 19.287079 |
| H | 15.991544 | 8.873560 | 21.033804 |
| H | 13.132286 | 11.018846 | 18.582043 |
| H | 14.208895 | 9.784901 | 17.920189 |
| H | 14.856430 | 11.356865 | 18.355983 |
| H | 13.606999 | 8.347174 | 21.219883 |
| H | 13.387867 | 8.185965 | 19.474105 |
| H | 12.364233 | 9.308794 | 20.381811 |
| H | 12.335986 | 11.230796 | 25.932537 |
| H | 12.888444 | 12.470502 | 24.780755 |
| H | 13.415594 | 10.965968 | 27.960049 |
| H | 15.167456 | 11.623331 | 29.589338 |
| H | 17.095296 | 13.003845 | 28.804448 |
| H | 17.241094 | 13.697453 | 26.459206 |
| H | 16.396917 | 14.379913 | 22.285779 |
| H | 15.344406 | 15.818814 | 22.344559 |
| H | 14.604628 | 14.212767 | 22.199559 |
| H | 16.556655 | 15.575094 | 25.802850 |
| H | 16.599659 | 16.462280 | 24.283006 |
| H | 17.547826 | 14.970233 | 24.455715 |
| H | 13.232155 | 14.819774 | 24.219488 |
| H | 14.078507 | 16.357811 | 24.485177 |
| H | 14.014747 | 15.148534 | 25.777996 |
| H | 13.059775 | 8.912155 | 25.578192 |
| H | 14.695405 | 9.559931 | 25.835005 |
| H | 12.687036 | 7.137694 | 24.192648 |
| H | 13.566562 | 5.168342 | 22.964510 |
| H | 15.985511 | 5.137346 | 22.323339 |
| H | 17.426369 | 7.025388 | 22.838835 |
| H | 18.390235 | 11.420429 | 24.439943 |
| H | 19.536175 | 10.556733 | 25.455262 |
| H | 17.854246 | 10.741261 | 25.998879 |
| H | 18.945960 | 8.153867 | 22.688751 |
| H | 20.096000 | 9.208307 | 23.504079 |
| H | 18.823960 | 9.912376 | 22.486102 |
| H | 17.783994 | 8.245552 | 26.332744 |
| H | 19.447609 | 8.104381 | 25.729440 |
| H | 18.158662 | 7.123588 | 25.018108 |

**(2-Rb)$_4$**

| | | | |
|---|---|---|---|
| C | 15.914915 | 8.234859 | 23.810939 |
| C | 14.533104 | 8.216645 | 24.192779 |
| C | 13.726606 | 7.118663 | 23.874946 |
| C | 14.213860 | 6.005810 | 23.196463 |
| C | 15.561707 | 5.996294 | 22.838566 |
| C | 16.384328 | 7.075047 | 23.133744 |
| C | 13.961740 | 9.297618 | 25.071394 |
| N | 13.608220 | 10.561743 | 24.384135 |
| C | 13.208992 | 11.593053 | 25.364682 |
| C | 14.311457 | 11.994414 | 26.305470 |
| C | 14.266606 | 11.579537 | 27.640683 |
| C | 15.247329 | 11.949455 | 28.554691 |
| C | 16.312150 | 12.732605 | 28.110363 |
| C | 16.377931 | 13.144309 | 26.784266 |
| C | 15.373841 | 12.817748 | 25.840916 |
| N | 15.421698 | 13.200741 | 24.491651 |
| C | 15.389258 | 14.640412 | 24.174497 |
| C | 14.069931 | 15.265997 | 24.667135 |
| N | 16.677725 | 9.351637 | 24.095394 |
| C | 18.117688 | 9.244802 | 24.429229 |
| C | 18.383867 | 8.103187 | 25.424723 |
| Ce | 15.838686 | 11.334345 | 23.096565 |
| N | 14.348997 | 11.021277 | 21.178303 |
| C | 14.394746 | 10.019112 | 20.089578 |
| C | 13.367623 | 8.888845 | 20.312305 |
| O | 17.271554 | 11.965325 | 22.077046 |
| C | 13.153102 | 11.740772 | 21.354236 |
| C | 12.288517 | 11.498414 | 22.461234 |
| C | 11.155422 | 12.297910 | 22.656092 |
| C | 10.821630 | 13.334616 | 21.790353 |
| C | 11.646518 | 13.581919 | 20.695229 |
| C | 12.782656 | 12.804909 | 20.495518 |
| C | 12.533723 | 10.341973 | 23.399494 |
| C | 15.792798 | 9.386832 | 20.090143 |
| C | 14.113218 | 10.583918 | 18.676397 |
| C | 15.448608 | 14.765053 | 22.644973 |
| C | 16.563339 | 15.472250 | 24.734808 |
| C | 18.485999 | 10.565678 | 25.128530 |
| C | 19.044551 | 9.116703 | 23.198889 |
| Rb | 16.563309 | 13.081572 | 19.751203 |
| H | 11.594736 | 10.103037 | 23.930673 |
| H | 12.815453 | 9.461301 | 22.820956 |
| H | 10.510568 | 12.084749 | 23.506561 |
| H | 9.933938 | 13.935318 | 21.965472 |

| | | | |
|---|---|---|---|
| H | 11.410793 | 14.385182 | 20.000832 |
| H | 13.415775 | 13.016770 | 19.636653 |
| H | 16.586557 | 10.134496 | 19.979258 |
| H | 15.892811 | 8.661295 | 19.275489 |
| H | 15.979818 | 8.849858 | 21.025540 |
| H | 13.108916 | 11.009283 | 18.602871 |
| H | 14.193006 | 9.788761 | 17.926876 |
| H | 14.827680 | 11.365133 | 18.379865 |
| H | 13.599327 | 8.332443 | 21.224617 |
| H | 13.374005 | 8.176465 | 19.478547 |
| H | 12.354234 | 9.297017 | 20.393682 |
| H | 12.342913 | 11.224924 | 25.943918 |
| H | 12.888006 | 12.466404 | 24.790695 |
| H | 13.433008 | 10.959813 | 27.966423 |
| H | 15.189920 | 11.621890 | 29.588754 |
| H | 17.109395 | 13.009837 | 28.796215 |
| H | 17.242560 | 13.705024 | 26.450682 |
| H | 16.416069 | 14.380029 | 22.303285 |
| H | 15.351133 | 15.812644 | 22.336682 |
| H | 14.631446 | 14.198072 | 22.180666 |
| H | 16.501832 | 15.586078 | 25.819676 |
| H | 16.550967 | 16.481182 | 24.304030 |
| H | 17.521527 | 15.004221 | 24.484260 |
| H | 13.213327 | 14.792154 | 24.176969 |
| H | 14.034012 | 16.341869 | 24.455918 |
| H | 13.963969 | 15.132770 | 25.748757 |
| H | 13.063803 | 8.909690 | 25.584665 |
| H | 14.700992 | 9.556755 | 25.835522 |
| H | 12.688315 | 7.132007 | 24.203207 |
| H | 13.565992 | 5.164312 | 22.970305 |
| H | 15.981506 | 5.139206 | 22.316111 |
| H | 17.420723 | 7.031033 | 22.824240 |
| H | 18.386816 | 11.419459 | 24.445385 |
| H | 19.530587 | 10.549906 | 25.460039 |
| H | 17.847432 | 10.734251 | 26.000096 |
| H | 18.943170 | 8.159176 | 22.682179 |
| H | 20.093955 | 9.207435 | 23.505041 |
| H | 18.825104 | 9.919383 | 22.487338 |
| H | 17.772117 | 8.238128 | 26.322733 |
| H | 19.437842 | 8.099460 | 25.724801 |
| H | 18.151514 | 7.121062 | 25.005346 |

**(2-Cs)$_4$**

| | | | |
|---|---|---|---|
| C | 15.900723 | 8.154889 | 23.906656 |
| C | 14.544432 | 8.062806 | 24.352885 |
| C | 13.788780 | 6.917238 | 24.085565 |
| C | 14.314583 | 5.823575 | 23.405121 |
| C | 15.644393 | 5.884733 | 22.989242 |
| C | 16.412349 | 7.016691 | 23.229085 |
| C | 13.955066 | 9.135855 | 25.231117 |
| N | 13.528964 | 10.363062 | 24.525719 |
| C | 13.169290 | 11.429636 | 25.483200 |
| C | 14.305855 | 11.940865 | 26.328306 |
| C | 14.360933 | 11.587304 | 27.679338 |
| C | 15.299974 | 12.130678 | 28.550634 |
| C | 16.204367 | 13.063303 | 28.044554 |
| C | 16.179185 | 13.420715 | 26.702324 |
| C | 15.243684 | 12.873898 | 25.785451 |
| N | 15.197444 | 13.153974 | 24.427670 |
| C | 15.438652 | 14.519289 | 23.927429 |
| C | 14.702796 | 15.577429 | 24.766974 |
| N | 16.600725 | 9.329575 | 24.132758 |
| C | 18.038644 | 9.312737 | 24.477026 |
| C | 18.366528 | 8.210913 | 25.498816 |
| Ce | 15.575229 | 11.197827 | 23.090078 |
| N | 14.012688 | 10.782278 | 21.328979 |
| C | 14.436269 | 10.307796 | 19.997256 |
| C | 13.269707 | 9.683537 | 19.212837 |
| O | 17.017121 | 11.848238 | 22.065196 |
| C | 12.820529 | 11.466962 | 21.502219 |
| C | 12.045585 | 11.216535 | 22.678303 |
| C | 10.871528 | 11.936188 | 22.917441 |
| C | 10.395738 | 12.897157 | 22.030355 |
| C | 11.120628 | 13.130211 | 20.862248 |
| C | 12.298170 | 12.438322 | 20.608023 |
| C | 12.410716 | 10.083904 | 23.601058 |
| C | 15.477633 | 9.203655 | 20.250663 |
| C | 15.131638 | 11.389189 | 19.133818 |
| C | 14.852280 | 14.562025 | 22.505125 |
| C | 16.938452 | 14.896471 | 23.813143 |
| C | 18.329263 | 10.668681 | 25.144107 |
| C | 18.978557 | 9.199388 | 23.253712 |
| Cs | 18.597356 | 13.471366 | 20.486077 |
| H | 11.523676 | 9.793023 | 24.191796 |
| H | 12.712433 | 9.222188 | 22.997202 |
| H | 10.300871 | 11.707888 | 23.816210 |
| H | 9.476263 | 13.436850 | 22.236775 |

| | | | |
|---|---|---|---|
| H | 10.771329 | 13.866303 | 20.140979 |
| H | 12.843276 | 12.663220 | 19.699389 |
| H | 16.369250 | 9.610574 | 20.744481 |
| H | 15.807245 | 8.750493 | 19.308005 |
| H | 15.060166 | 8.417874 | 20.885800 |
| H | 14.443346 | 12.159428 | 18.776115 |
| H | 15.602624 | 10.935150 | 18.252074 |
| H | 15.904982 | 11.866492 | 19.745340 |
| H | 12.826065 | 8.867420 | 19.791859 |
| H | 13.623222 | 9.273819 | 18.259421 |
| H | 12.480978 | 10.409206 | 18.997194 |
| H | 12.360992 | 11.062051 | 26.140386 |
| H | 12.772912 | 12.259475 | 24.889426 |
| H | 13.621396 | 10.883994 | 28.058743 |
| H | 15.317023 | 11.844412 | 29.598024 |
| H | 16.948683 | 13.512456 | 28.698855 |
| H | 16.918631 | 14.126458 | 26.344233 |
| H | 15.381524 | 13.862519 | 21.844789 |
| H | 14.939400 | 15.566864 | 22.072595 |
| H | 13.796135 | 14.280632 | 22.513140 |
| H | 17.397626 | 15.119348 | 24.779091 |
| H | 17.066528 | 15.791037 | 23.186384 |
| H | 17.476486 | 14.051933 | 23.369004 |
| H | 13.629587 | 15.363084 | 24.780494 |
| H | 14.847906 | 16.578033 | 24.342375 |
| H | 15.055542 | 15.594342 | 25.801465 |
| H | 13.090407 | 8.722748 | 25.780592 |
| H | 14.707834 | 9.442557 | 25.964478 |
| H | 12.764722 | 6.878094 | 24.453503 |
| H | 13.708454 | 4.942168 | 23.217791 |
| H | 16.089937 | 5.044159 | 22.461254 |
| H | 17.432966 | 7.034194 | 22.867338 |
| H | 18.166103 | 11.493622 | 24.439000 |
| H | 19.373157 | 10.726123 | 25.475193 |
| H | 17.680986 | 10.821149 | 26.011263 |
| H | 18.934028 | 8.219463 | 22.772161 |
| H | 20.020944 | 9.363991 | 23.556460 |
| H | 18.701385 | 9.959350 | 22.515355 |
| H | 17.746443 | 8.332243 | 26.392886 |
| H | 19.418643 | 8.270531 | 25.800591 |
| H | 18.186959 | 7.209433 | 25.099377 |

## References


1. C. H. Booth, D. Kazhdan, E. L. Werkema, M. D. Walter, W. W. Lukens, E. D. Bauer, Y.-J. Hu, L. Maron, O. Eisenstein, M. Head-Gordon and R. A. Andersen, *J. Am. Chem. Soc.*, 2010, **132**, 17537-17549.
2. C. H. Booth, M. D. Walter, D. Kazhdan, Y.-J. Hu, W. W. Lukens, E. D. Bauer, L. Maron, O. Eisenstein and R. A. Andersen, *J. Am. Chem. Soc.*, 2009, **131**, 6480-6491.
3. R. L. Halbach, G. Nocton, C. H. Booth, L. Maron and R. A. Andersen, *Inorg. Chem.*, 2018, **57**, 7290-7298.
4. C. H. Booth, M. D. Walter, M. Daniel, W. W. Lukens and R. A. Andersen, *Phys. Rev. Lett.*, 2005, **95**, 267202.
5. K. O. Kvashnina, S. M. Butorin and P. Glatzel, *J. Anal. At. Spectrom.*, 2011, **26**, 1265-1272.
6. O. Hirsch, K. O. Kvashnina, L. Luo, M. J. Süess, P. Glatzel and D. Koziej, *Proc. Natl. Acad. Sci.*, 2015, **112**, 15803.
